# Activation of Bmp2-Smad1 Signal and Its Regulation by Coordinated Alteration of H3K27 Trimethylation in *Ras*-Induced Senescence

**DOI:** 10.1371/journal.pgen.1002359

**Published:** 2011-11-03

**Authors:** Atsushi Kaneda, Takanori Fujita, Motonobu Anai, Shogo Yamamoto, Genta Nagae, Masato Morikawa, Shingo Tsuji, Masanobu Oshima, Kohei Miyazono, Hiroyuki Aburatani

**Affiliations:** 1 Genome Science Division, Graduate School of Medicine, The University of Tokyo, Tokyo, Japan; 2 PRESTO, Japan Science and Technology Agency (JST), Tokyo, Japan; 3 Laboratory for Systems Biology and Medicine, Research Center for Advanced Science and Technology (RCAST), Graduate School of Medicine, The University of Tokyo, Tokyo, Japan; 4 Department of Molecular Pathology, Graduate School of Medicine, The University of Tokyo, Tokyo, Japan; 5 Division of Genetics, Cancer Research Institute, Kanazawa University, Kanazawa, Japan; Stanford University School of Medicine, United States of America

## Abstract

Cellular senescence involves epigenetic alteration, e.g. loss of H3K27me3 in *Ink4a*-*Arf* locus. Using mouse embryonic fibroblast (MEF), we here analyzed transcription and epigenetic alteration during *Ras*-induced senescence on genome-wide scale by chromatin immunoprecipitation (ChIP)-sequencing and microarray. *Bmp2* was the most activated secreted factor with H3K4me3 gain and H3K27me3 loss, whereas H3K4me3 loss and *de novo* formation of H3K27me3 occurred inversely in repression of nine genes, including two BMP-SMAD inhibitors *Smad6* and *Noggin*. DNA methylation alteration unlikely occurred. *Ras*-activated cells senesced with nuclear accumulation of phosphorylated SMAD1/5/8. Senescence was bypassed in *Ras*-activated cells when Bmp2/Smad1 signal was blocked by *Bmp2* knockdown, *Smad6* induction, or *Noggin* induction. Senescence was induced when recombinant BMP2 protein was added to *Bmp2*-knocked-down *Ras*-activated cells. Downstream Bmp2-Smad1 target genes were then analyzed genome-wide by ChIP-sequencing using anti-Smad1 antibody in MEF that was exposed to BMP2. Smad1 target sites were enriched nearby transcription start sites of genes, which significantly correlated to upregulation by BMP2 stimulation. While *Smad6* was one of Smad1 target genes to be upregulated by BMP2 exposure, *Smad6* repression in *Ras*-activated cells with increased enrichment of Ezh2 and gain of H3K27me3 suggested epigenetic disruption of negative feedback by Polycomb. Among Smad1 target genes that were upregulated in *Ras*-activated cells without increased repressive mark, *Parvb* was found to contribute to growth inhibition as *Parvb* knockdown lead to escape from senescence. It was revealed through genome-wide analyses in this study that Bmp2-Smad1 signal and its regulation by harmonized epigenomic alteration play an important role in *Ras*-induced senescence.

## Author Summary

To avoid becoming cancer cells, cells have a barrier system to block cellular proliferation by falling into irreversible growth arrest, so-called cellular senescence. For future strategy of cancer treatment, it is important to understand how cancer occurs, and investigation of underlying mechanism in senescence can lead to clarification of carcinogenesis mechanism. Epigenetic mechanism including DNA methylation and histone modification may be important to regulate gene expressions properly in senescence. Here, taking advantage of recent technical and methodological advance of genome-wide analyses, we examine epigenome and gene expression alteration in senescence induced by *Ras* oncogene. We identify that Bmp2-Smad1 signal is critical. We further examine downstream target genes of this critical signal on a genome-wide scale. We show dynamic and coordinated H3K27me3 alteration, e.g. activation of Bmp2 by loss of H3K27me3, repression of the signal inhibitors and the negative feedback loop by gain of H3K27me3, and selective activation of downstream target genes that may contribute to growth arrest. Our findings are helpful in understanding the importance of epigenetic regulation and a critical signal in the physiological barrier system against oncogenic transformation and the importance of disruption of BMP-SMAD signal in cancer, and they may provide an idea how cancer with *Ras* mutation occurs.

## Introduction

Cellular senescence was first described as the limited replicative capacity of primary cells in culture [1]. Activated oncogenes can induce premature form of cellular senescence, and cells fall into irreversible arrest to block cellular proliferation [2], [3]. In addition to cell death programs such as apoptosis and autophagy, oncogene-induced senescence is recognized as a potent barrier against oncogenic transformation, suppressing unscheduled proliferation of early neoplastic cells [4]–[7].

Replicative senescence and oncogene-induced senescence are known to comprise activation of tumor suppressor pathways including p16^Ink4a^-Rb and p19^Arf^ (p14^ARF^ in human)-p53 signaling cascades. Genetic and epigenetic inactivation of these genes in cancer supported their crucial roles in senescence as barriers to tumorigenesis [8], [9]. Although the roles of RB and p53 signaling pathways in senescence are undisputed, it has become clear that other factors are also involved. Expression of secreted factors, or “senescence-messaging secretome”, has been proposed as an example of such mechanisms [10], [11]. The induction of senescence required several secreted factors including members of Wnt, insulin, transforming growth factor-β, plasmin and interleukin signaling cascades [11].

Epigenetic mechanism is also suggested to play important roles in senescence. When human fibroblasts senesced, heterochromatic regions condensed to form senescence-associated heterochromatic foci, where regions with histone H3K9 trimethylation (H3K9me3) gathered [12], and were recently shown to restrain DNA damage response [13]. Expression of Jhdm1b, a demethylase specific for H3K36me2, caused cell immortalization or leukemic transformation depending on its demethylase activity on *p15* ^Ink4b^, and its knock down resulted in cellular senescence [14], [15]. *INK4A* and *ARF* region in young cells was repressed by H3K27me3 imposed by the Polycomb Group proteins, and the repressive mark was lost during oncogene-induced senescence, resulting in expression of *p16* and *p19*; the loss of repressive mark was also detected when mouse embryonic fibroblast (MEF) underwent stress-induced senescence around seven passages [16]–[19]. Jmjd3, a histone demethylase for H3K27, was found to be essential in senescence, and its knock down lead to escape from senescence sustaining repression of *p16* by H3K27me3 [20], [21].

In the previous studies, we comprehensively analyzed aberrant promoter DNA methylation in colorectal cancer and reported three distinct DNA methylation epigenotypes [22], [23]. Distinct methylation epigenotypes significantly correlated to different oncogene mutation statuses, suggesting that epigenotypes of cancer might perhaps be requisite phenotype of aberrant methylation to escape from oncogene-induced senescence by inactivation of critical factors of senescence [23], [24]. To gain insight in phenotype of critical gene inactivation in oncogene-mutation(+) cancer, we aim to clarify critical genes/signals/phenomena in oncogene-induced senescence in normal cells in this study.

Here we perform genome-wide analyses of epigenetic and gene expression changes in *Ras*-indeced senescence using mouse embryonic fibroblasts (Figure S1). We show that Bmp2/Smad1 signal is critical in *Ras*-induced senescence, and is regulated by coordinated epigenomic alteration. We further examine downstream target genes of this critical signal on genome-wide scale, and show that the epigenomic regulation of the signal involves disruption of negative feedback loop, and that activated downstream targets actually include a gene to contribute to growth arrest.

## Results

### Gene expression analysis

**Figure 1:**
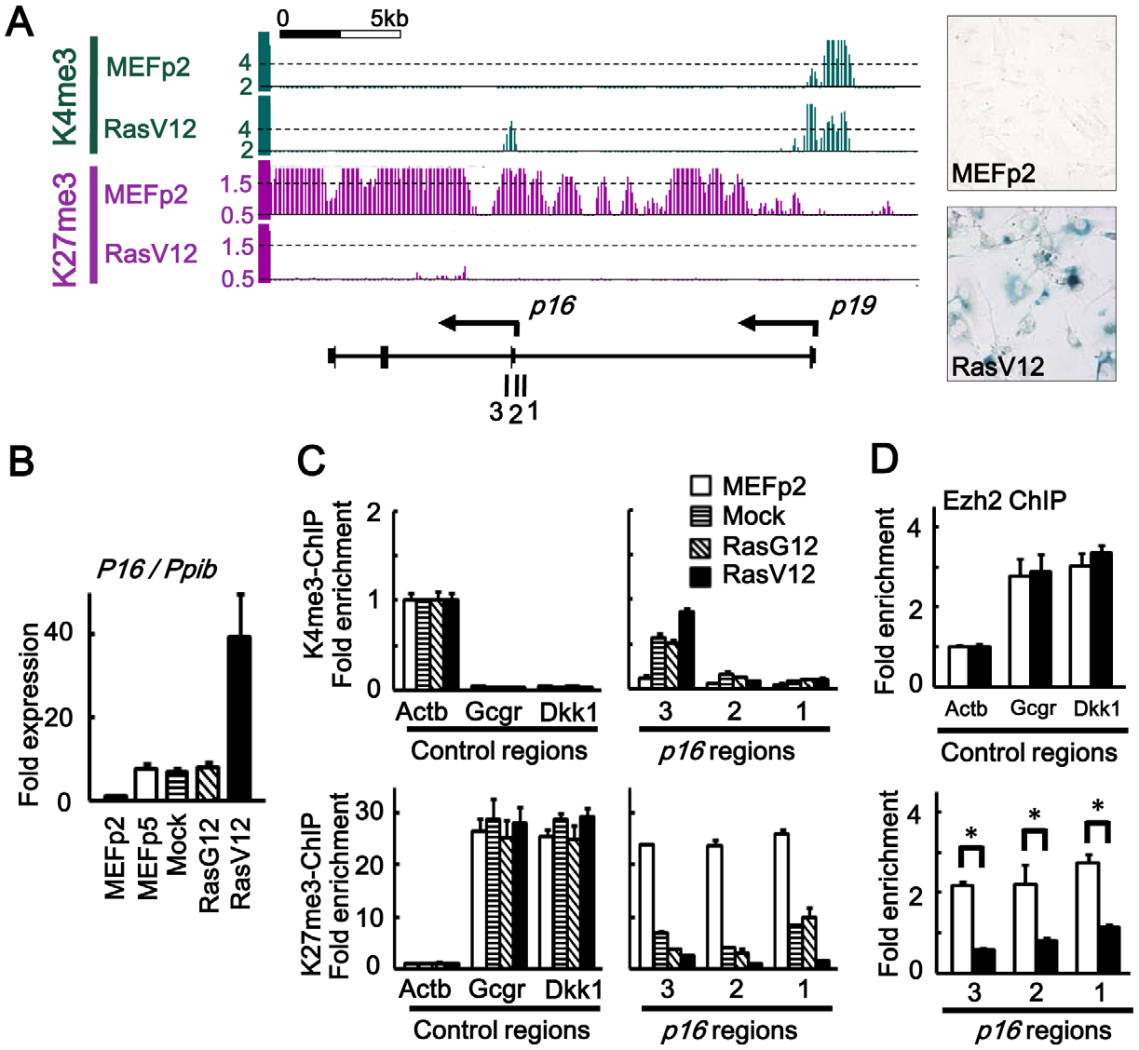
Epigenomic alteration of *Ink4a*-*Arf* locus. (*A*) H3K4me3 and H3K27me3 mapped by ChIP-sequencing. *Y-axis*, number of mapped reads per million reads, within window size of 300 bp for H3K4me3 and 500 bp for H3K27me3. *1, 2, 3*; regions for ChIP-PCR. Right panels showed SA-βgal(-) in MEFp2 and SA-βgal(+) in RasV12 cells. (*B*) Real-time RT-PCR for *p16*, normalized to *Ppib*. Fold expression levels compared to MEFp2 were shown. *p16* expression was partially increased in MEFp5, Mock and RasG12 due to stress-induced senescence during passages, but markedly increased in RasV12 cells. (*C*) Real-time ChIP-PCR for H3K4me3 and H3K27me3. In each sample of MEFp2, Mock cells, RasG12 cells and RasV12 cells, relative enrichment compared to *Actb* was shown. *Actb*, a control region for H3K4me3. *Gcgr* and *Dkk1*, control regions for H3K27me3. Gain of H3K4me3 and loss of H3K27me3 around *p16* TSS were partially detected in Mock and RasG12 cells indicating partial epigenetic alteration during passages, but markedly detected in RasV12 cells. (*D*) Real-time ChIP-PCR for Ezh2. Ezh2 enrichment was significantly decreased around *p16* TSS (*P<0.05, *t*-test).

To induce cellular senescence, mouse embryonic fibroblasts after two passages (MEFp2) was infected with retrovirus of oncogenic *Ras* (RasV12) with N-terminal FLAG tag and cultured through day 10 (Figure S2A). RasV12-infected cells (RasV12 cells) showed significant increase in number of SA-βgal(+) cells, compared to MEFp2, MEF passed three more times without infection (MEFp5), mock-infected cells (Mock cells), and wild type *Ras* (RasG12)-infected cells (Figure 1A and Figure S2B).

Global gene expression analysis was performed using expression array. In RasV12 cells on day 10, 822 genes were upreglated and 735 genes downregulated, by >5-fold compared to MEFp2 (Tables S1, S2). Gene annotation enrichment analysis suggested that genes related to secreted protein (P = 1.8×10^−19^), extracellular region (P = 1.2×10^−21^), and differentiation/development (P = 3.8×10^−10^), e.g. *Bmp2* and *Igfbp3*, were upregulated, supporting the importance of secreted factor expression in senescence. Genes related to cell cycle (P = 7.2×10^−22^) such as *Cdc6* and *Mcm5* were enriched in downregulated genes, indicating growth arrest. Also genes related to secreted protein (P = 7.9×10^−18^) and extracellular region (P = 9.2×10^−14^) such as *Bmp4* and *Tgfb2* were enriched in downregulated genes, suggesting that dynamic control of secretome by activation and repression of secreted factors occurred during senescence.

### Epigenomic alteration analysis

To analyze epigenomic gene regulation during *Ras*-induced senescence, we selected H3K4me3 as an active mark and H3K27me3 as a repressive mark, and mapped them by Chromatin immunoprecipitation (ChIP)-sequencing. As reported, H3K27me3 mark at *p16* ^Ink4a^-*p19* ^Arf^ locus in MEFp2 was markedly lost in RasV12 cells (Figure 1A). ChIP-sequencing of H3K4me3 showed concurrent gain of the active mark around *p16* transcription start site (TSS), which reflected increase of *p16* expression in RasV12 (Figure 1B). By quantitative ChIP-PCR, significant gain of H3K4me3 and loss of H3K27me3 were validated in RasV12 cells, compared to MEFp2 (Figure 1C). Gain of H3K4me3 and loss of H3K27me3 were also detected at intermediate level in Mock and RasG12 cells (Figure 1C). Expression of *p16* was also partially increased in Mock and RasG12 cells, at the similar level to MEFp5 (Figure 1B). These indicated that *p16* expression could be induced partially by gain of H3K4me3 and H3K27me3 during passages, which was in agreement with the previous report of gradual H3K27me3 loss in stress-induced senescence during 5–7 passages [16], [18], but more marked alteration occurred at this locus in *Ras*-induced senescence. Enrichment of Ezh2, a member of the Polycomb Group proteins, was also analyzed by ChIP-PCR, and it was significantly decreased around *p16* TSS in RasV12 cells compared to MEFp2 (Figure 1D).

When analyzing distribution of 36-bp reads mapped around TSS of 20,232 genes, the mapped reads were enriched within ±2 kb of TSS, mainly ±1 kb of TSS (Figure S3A), for both H3K4me3 and H3K27me3. We counted mapped reads within a window of genomic region, so that the number of mapped reads per million reads within a window is regarded as epigenetic status of the center position of the window. Within ±2 kb from TSS of each gene, the maximum number of mapped reads per million reads in a window size of 300 bp (H3K4me3) or 500 bp (H3K27me3) was regarded as the epigenetic status of each gene. A wider window was necessary for H3K27me3 because distribution of H3K27me3 was rather wide than H3K4me3 (Figure 1A and Figure S3A). The number of genes with repressive H3K27me3 mark was generally decreased in RasV12 cells (Figure S3B), in agreement of the previous reports [20], [21] that expression of Jmjd3 was increased during senescence, whereas expression of Ezh2 was decreased (Figure S4). It was expected that genes activated by losing H3K27me3 might exist other than *p16* and *p19*, because of the decrease of genes with H3K27me3 mark in RasV12 cells.

### Integrated analysis of epigenomic alteration and expression

Among 20,232 genes with epigenomic alteration analyzed, 16,793 genes were also analyzed for expression on array (Figure S5). For epigenetic status of H3K4me3, 9,164 genes in MEFp2 and 8,841 genes in RasV12 showed >4 reads per million reads around TSS, and regarded as H3K4me3(+). Similarly, 7,140 and 7,354 genes respectively with <3 reads per million reads were regarded as H3K4me3(-). Markedly higher expression levels of H3K4me3(+) genes than H3K4me3(-) genes were confirmed by comparing the mean of expression levels (Figure S5A). For H3K27me3, 2,612 and 2,370 genes with >1.5 reads per million reads around TSS were regarded as H3K27me3(+), and 13,205 and 12,841 genes with <1 were as H3K27me3(-) in this study. H3K27me3(+) genes were markedly repressed than H3K27me3(-) genes (Figure S5B).

**Figure 2:**
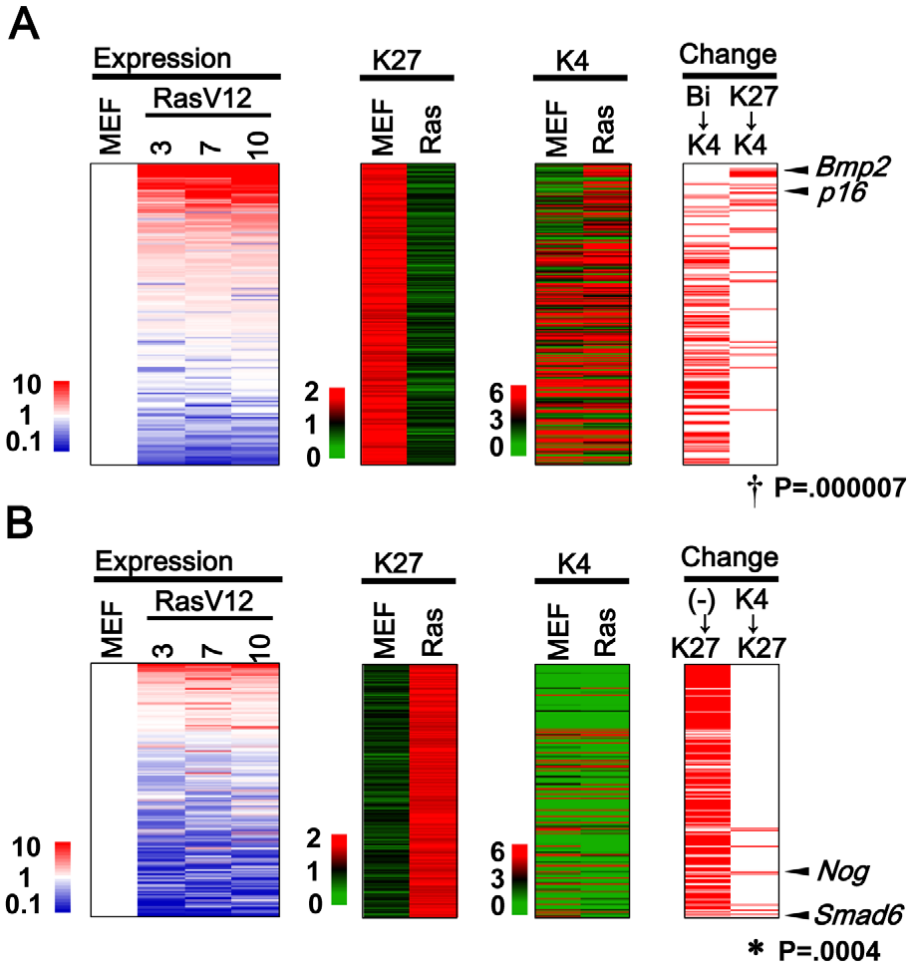
Genes with H3K27me3 alteration. (*A*) 284 Genes losing H3K27me3 mark, i.e. from >1.5 in MEFp2 to <1.0 in RasV12 cells (*K27* column), were sorted by the fold expression change between MEFp2 and mean of RasV12 cells day3, 7, and 10 (*Expression* column). Upregulated genes were sorted upward (*red*). 30 genes losing H3K27me3 and gaining H3K4me3 simultaneously showed significant enrichment upward (*Change K27→K4* column, ^†^P = 0.000007, Kolmogorov-Smirnov test), e.g. *p16*. *Bmp2* was found to be the most activated secreted factor among the 30 genes, and activated more than *p16*. Genes are listed in Table S3. 110 genes with bivalent modification in MEFp2 lost H3K27me3 and sustained H3K4me3 mark in RasV12 cells (*Bi→K4*), but did not show significant enrichment upward (P = 0.9). (*B*) 239 genes gaining H3K27me3 mark, i.e. from <1.0 in MEFp2 to <1.5 in RasV12 cells (*K27* column). 189 genes had neither modification in MEFp2 (*K27* column and *K4* column), generally showed very low expression, and did not show significant enrichment downward (P = 1, *(-)→K27*). However, 9 genes gained H3K27me3 and lost H3K4me3 simultaneously, showed significant enrichment downward (*K4→K27*, *P = 0.0004), and included *Smad6* and *Nog*. Genes were listed in Table S4.

Among 284 genes losing H3K27me3 in RasV12 cells, 30 genes losing H3K27me3 and gaining H3K4me3 simultaneously, like *p16*, showed significant enrichment in upregulated genes among the 284 genes (P = 0.000007, Kolmogorov-Smirnov test, Figure 2A). Among the 30 genes (listed in Table S3), *Bmp2*, a secreted factor for BMP/SMAD pathway, was found to be the most upregulated secreted factor and activated more than *p16* (Figure 2A). Interestingly, 110 genes modified bivalently in MEFp2 showed loss of H3K27me3 and sustained H3K4me3 mark in RasV12 cells, but did not show significant enrichment in upregulated genes (P = 0.9, Figure 2A).

Not only genes with H3K27me3 loss, but also there were as many as 239 genes showing H3K27me3 gain in RasV12 cells. Nine genes gaining H3K27me3 and losing H3K4me3 simultaneously showed significant enrichment in downregulated genes (P = 0.0004, Figure 2B. Genes are listed in Table S4). Very interestingly, two of the nine genes were *Smad6* and *Nog*, inhibitors for BMP-SMAD pathway [25]. The majority, 189 of the 239 genes, had neither modification in MEFp2 with very low expression levels. These genes acquired *de novo* H3K27me3 mark in RasV12 cells, but did not show any more downregulation (P = 1, Figure 2B).

### Upregulation of *Bmp2* in senescence

**Figure 3:**
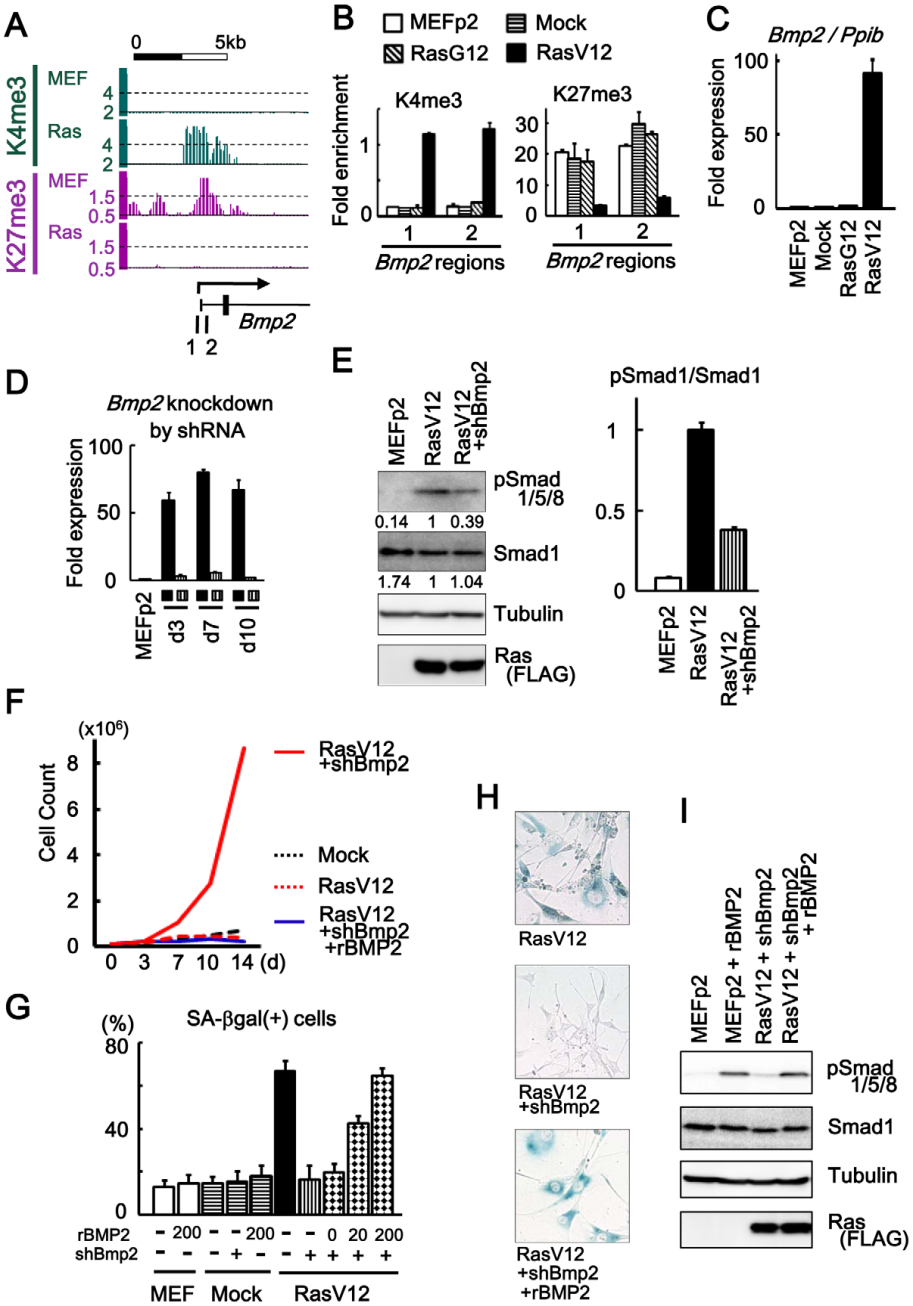
*Bmp2* upregulation in senescence. (*A*) Maps around TSS of *Bmp2* showing gain of H3K4me3 and loss of H3K27me3. *1, 2*; regions for ChIP-PCR. (*B*) Real-time ChIP-PCR showing H3K4me3 gain and H3K27me3 loss specifically in RasV12 cells. There was no alteration in Mock cells and RasG12 cells, thus no alteration during passages. (*C*) Real-time RT-PCR analysis for *Bmp2*, showing upregulation to 92-fold specifically in RasV12 cells. There was no increase in Mock cells and RasG12 cells, thus no alteration during passages. (*D*) Real-time RT-PCR showing knock-down of *Bmp2* to 0.05–0.08 fold by shRNA on days 3, 7, 10. *Closed square*, infected with RasV12 cells. *Vertical-striped*, infected with RasV12 and shBmp2. (*E*) Western blot analysis showing increased level of Smad1/5/8 phosphorylation in RasV12 cells, and decrease in *Bmp2*-knocked-down RasV12 cells. RasV12 with N-terminal FLAG was detected using anti-FLAG antibody. Numbers under bands of phosphorylated Smad1/5/8 and pan-Smad1 showed densities relative to the band for RasV12 cells (*left panel*), and the ratio of densities for phosphorylated Smad1/5/8 to pan-Smad1 was decreased to 0.38-fold in *Bmp2*-knocked-down RasV12 cells (*right panel*). (*F*) Growth curve. *Bmp2*-knocked-down RasV12 cells (*red solid*) showed continual growth faster than Mock cells (*black dotted*). Growth of *Bmp2*-knocked-down Mock cells (co-infection of shBmp2 and Mock retroviruses, data not shown) was similar to Mock cells. When cultured with rBMP2 (*blue solid*), the cells senesced like RasV12 cells (*red dotted*). (*G*) The number of SA-βgal(+) cells (%). When 0, 20, and 200 ng/mL of rBMP2 protein (R&D systems #355-BM) were added to *Bmp2*-knocked-down RasV12 cells in culture medium with 10% serum, the number of SA-βgal(+) cells was increased in dose-dependent manner. The number of SA-βgal(+) cells was not increased when Mock cells or MEF without infection was exposed to 200 ng/mL of rBMP2 protein. (*H*) SA-βgal staining. *Bmp2*-knocked-down RasV12 cells showed decreased number of SA-βgal(+) cells compared to RasV12 cells. When cultured with rBMP2 protein, the number of SA-βgal(+) cells was increased (representative result at 200 ng/mL rBMP2). (*I*) Western blot analysis showing increased level of Smad1/5/8 phosphorylation when exposed to 200 ng/mL of rBMP2.

Around TSS of *Bmp2*, a secreted factor for BMP-SMAD pathway, loss of H3K27me3and gain of H3K4me3 were validated by quantitative ChIP-PCR (Figure 3A, 3B). ChIP-PCR also showed that Ezh2 enrichment was significantly decreased around *Bmp2* in RasV12 cells (Figure S6).

ChIP-PCR showed that H3K4me3 and H3K27me3 levels in MEFp2 were sustained in Mock and RasG12, but specifically altered in RasV12 cells (Figure 3B). Quantitative RT-PCR showed very low level of *Bmp2* expression in MEFp2, Mock cells and RasG12 cells, but marked increase to 91.6-fold in RasV12 cells (Figure 3C). *Bmp2* activation thus occurred specifically in *Ras*-induced senescence, different from *p16* that partially showed increased expression and histone methylation alteration during passages (Figure 1C).

Retrovirus to express shRNA against *Bmp2* (shBmp2) was infected together with RasV12 infection, to knock down *Bmp2* to 0.04–0.08 fold on days 3, 7, and 10 (Figure 3D). *Bmp2*-knocked-down RasV12 cells escaped from senescence with decreased number of SA-βgal(+) cells compared to RasV12 cells. While Smad1/5/8 is known to serve principally as substrates for BMP receptors [26], western blotting analysis revealed phosphorylation of Smad1/5/8 in RasV12 cells (Figure 3E). Decrease of Smad1/5/8 phosphorylation level was also shown in *Bmp2*-knocked-down RasV12 cells (Figure 3E), and continual cell growth faster than Mock cells (Figure 3F). To confirm that this escape from senescence was specifically due to *Bmp2* knockdown, *Bmp2*-knocked-down RasV12 cells were cultured with recombinant BMP2 protein (rBMP2, R&D systems #355-BM) at 0, 20 and 200 ng/mL in culture medium with 10% serum. The cells showed increased number of SA-βgal(+) cells in dose-dependent manner, even to the level of RasV12 cells when rBMP2 was at 200 ng/mL (Figure 3G, 3H). The level of Smad1/5/8 phosphorylation was increased when rBMP was added (Figure 3I), and growth curve showed growth arrest similar to senescent RasV12 cells (Figure 3F). These results indicated that *Bmp2* upregulation plays an important role in *Ras*-induced senescence. There was no increase of SA-βgal(+) cells when Mock cells or MEF cells without infection were exposed to rBMP2 at 200 ng/mL, indicating that increase of BMP2 alone is not enough to induce cellular senescence (Figure 3G).

### Repression of *Smad6* in senescence

**Figure 4:**
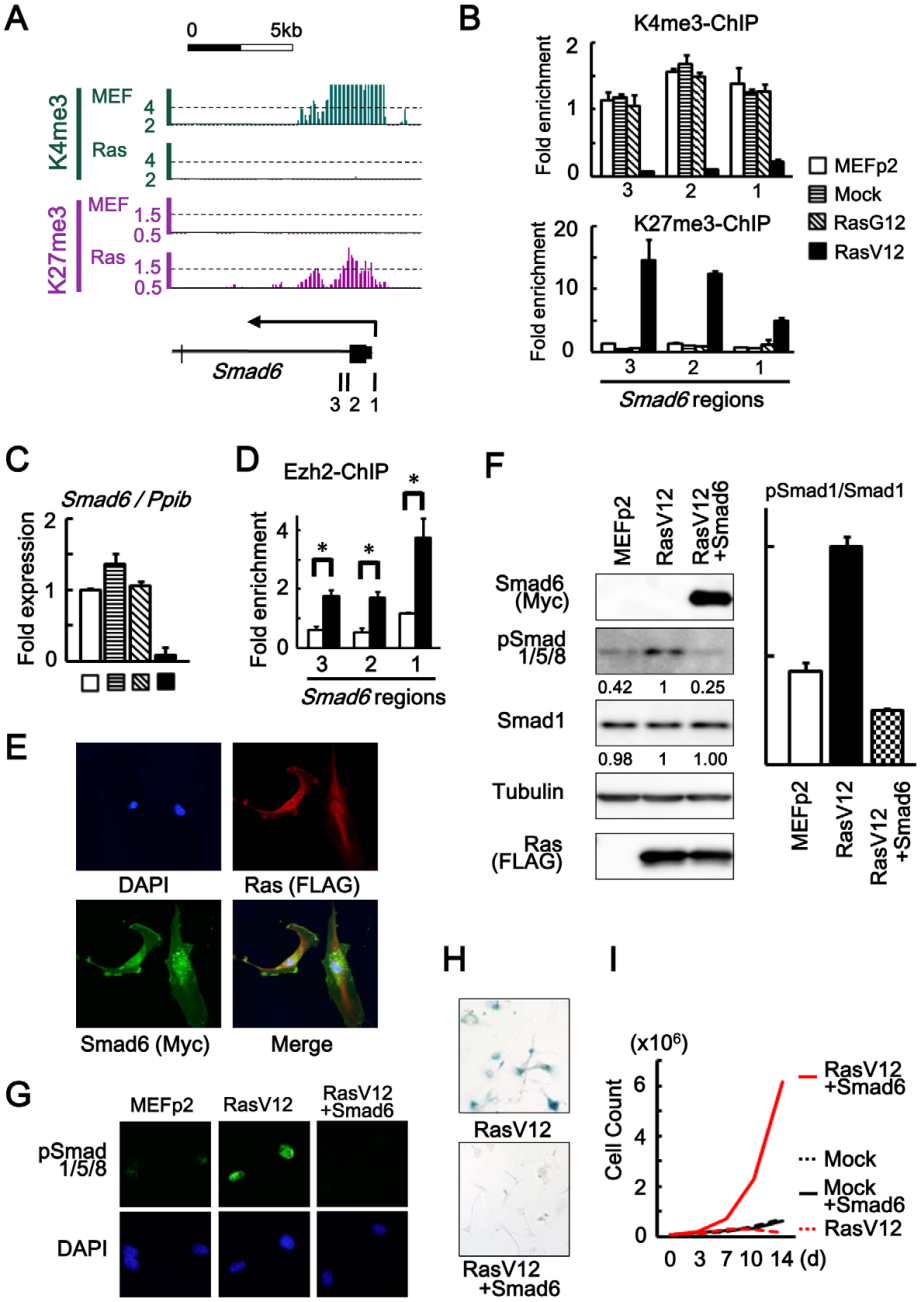
*Smad6* repression in senescence. (*A*) Loss of H3K4me3 and gain of H3K27me3 around TSS of *Smad6*, shown by ChIP-sequencing. *1, 2, 3*; regions for ChIP-PCR. (*B*) Real-time ChIP-PCR showing H3K4me3 loss and H3K27me3 gain specifically in RasV12 cells. There was no alteration in Mock cells and RasG12 cells, thus no alteration during passages. (*C*) Real-time RT-PCR analysis of *Smad6*, showing repression to 0.05-fold specifically in RasV12 cells. There was no repression in Mock cells and RasG12 cells, thus no alteration during passages. (D) Real-time ChIP-PCR for Ezh2 showing increased enrichment of Ezh2 around *Smad6* TSS in RasV12 (*P<0.05). (E) Cellular immunofuorescence for RasV12 and Smad6. RasV12 with N-terminal FLAG and Smad6 with N-terminal Myc were detected using anti-FLAG and anti-Myc antibodies. Simultaneous expression of RasV12 and Smad6 proteins in Smad6-introduced RasV12 cells was confirmed (See also Figure S7). (*F*) Western blot analysis showing increased level of Smad1/5/8 phosphorylation in RasV12, and decrease in *Smad6*-introduced RasV12 cells. Numbers under bands of phosphorylated Smad1/5/8 and pan-Smad1 showed densities relative to the band for RasV12 cells (*left panel*), and the ratio of densities for phosphorylated Smad1/5/8 to pan-Smad1 was decreased to 0.25-fold in *Smad6*-introduced RasV12 cells (*right panel*). (*G*) Cellular immunofuorescence of phosphorylated Smad1/5/8. Nuclear accumulation of phosphorylated Smad1/5/8 was detected in RasV12 cells, but not in *Smad6*-introduced RasV12 cells. (*H*) SA-β-gal staining. The number of SA-β-gal(+) cells were significantly decreased in *Smad6*-introduced RasV12 cells compared with RasV12 (See also Figure S8). (I) Growth curve. *Smad6*-introduced RasV12 cells showed continual growth faster than Mock cells or *Smad6*-introduced mock cells.

As for *Smad6*, a specific inhibitor for BMP-SMAD pathway, gain of H3K27me3 and loss of H3K4me3 in RasV12 cells were found and validated by quantitative ChIP-PCR (Figure 4A, 4B). H3K27me3 and H3K4me3 levels in MEFp2 were sustained in Mock and RasG12 cells, and altered specifically in RasV12 cells. This indicated that these alterations of histone methylation were not detected in stress-induced senescence during passages, but specifically occurred in *Ras*-induced senescence, like *Bmp2*. Markedly decreased expression of *Smad6* to 0.05-fold specifically in RasV12 cells was also validated by quantitative RT-PCR, while there was no repression of *Smad6* during passages (Figure 4C). Ezh2 enrichment was also analyzed by ChIP-PCR (Figure 4D). This histone methyltransferase for H3K27 was significantly increased around TSS of *Smad6* in RasV12 cells. It was indicated that Ezh2 was recruited to this *de novo* H3K27 trimethylation site, and that repression mechanism by *de novo* H3K27me3 was still active although *Ezh2* expression level itself was downregulated during senescence, and *Jmjd3* expression level was upregulated (Figure S4A).

Smad6 with N-terminal Myc tag was introduced to MEF by retroviral infection together with RasV12 virus, and their simultaneous expression was confirmed by cellular immunofluorescence (Figure 4E and Figure S7). Western blotting analysis and cellular immunofluorescence showed decrease of Smad1/5/8 phosphorylation in *Smad6*-introduced RasV12 cells compared to RasV12 cells (Figure 4F, 4G). *Smad6*-introduced RasV12 cells showed decreased number of SA-βgal(+) cells compared to RasV12 cells (Figure 4H and Figure S8) and showed continual cell growth faster than Mock cells or *Smad6*-introduced Mock cells (Figure 4I). These data indicated that *Smad6* repression was important in *Ras*-induced senescence.

### Repression of *Nog* in senescence

**Figure 5:**
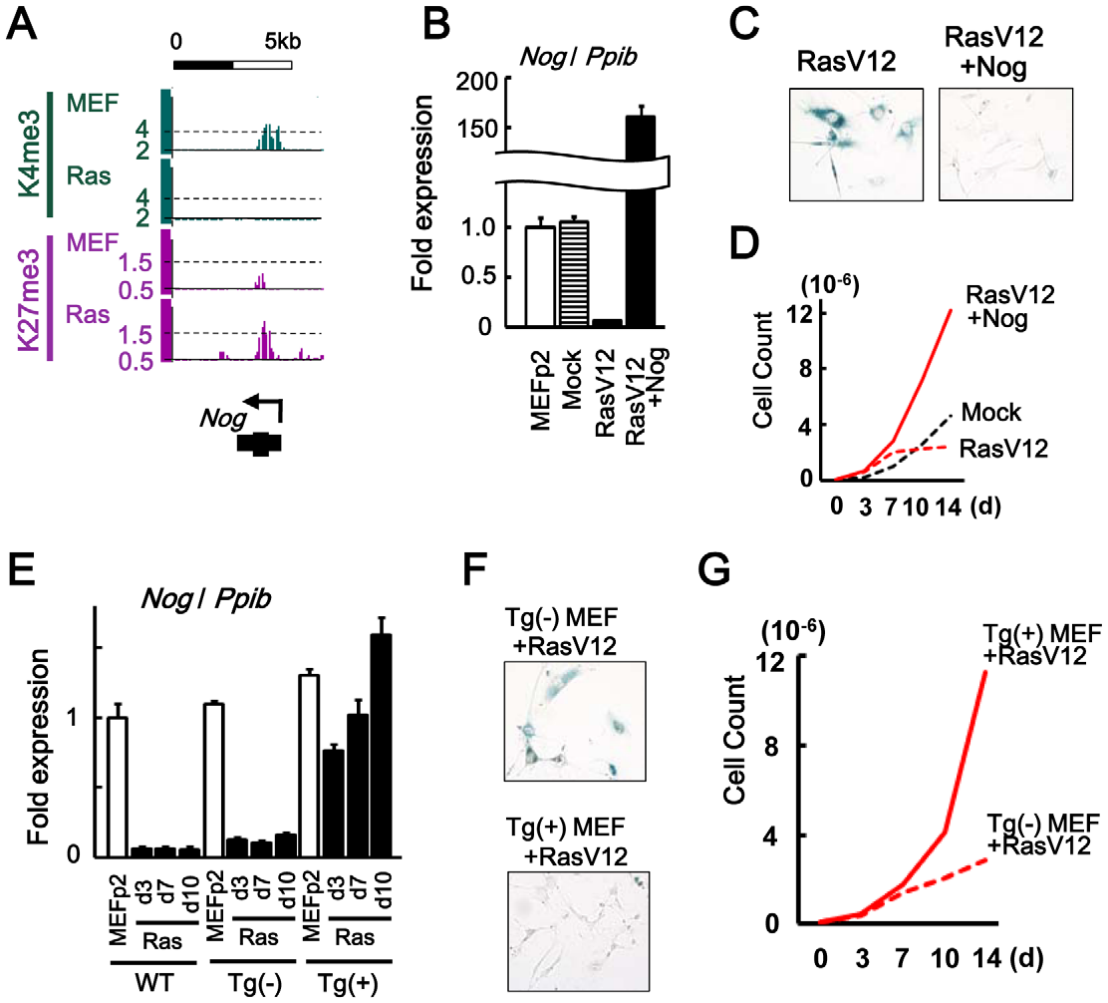
*Nog* repression in senescence. (*A*) Maps around TSS of *Nog*, showing increase of H3K4me3 and decrease of H3K27me3. (*B*) Real-time RT-PCR analysis of *Nog*, showing repression to 0.06-fold in RasV12 cells, and overexpression to 159-fold in *Nog*-introduced RasV12 cells. (C) SA-βgal staining. The number of SA-βgal(+) cells were decreased in *Nog*-introduced RasV12 cells compared with RasV12 cells. (*D*) Growth curve. *Nog*-introduced RasV12 cells showed continual growth. (*E*) Real-time RT-PCR analysis. *WT*, wild-type C57/B6 MEF. *Tg(-)*, Krt19-Nog transgene (-). *Tg(+)*, Krt19-Nog transgene (+). *Nog* was repressed to 0.09–0.15 fold by RasV12 infection in Tg(-) MEF, similar to wild type MEF. *Nog* expression in Tg(+) MEF was detected at physiological level, and was not repressed by RasV12 infection. The increase of *Nog* expression from day 3 to day 10 might be due to selection. (*F*) The number of SA-βgal(+) cells were less in Tg(+) MEF than Tg(-) MEF after RasV12 infection. (*G*) Tg(+) MEF showed faster growth compared to Tg(-) MEF, after Rasv12 infection.

*Nog*, another inhibitor for BMP-SMAD pathway, was repressed to 0.06-fold in RasV12 cells also by losing H3K4me3 and gaining H3K27me3 (Figure 5A, 5B). Introduction of *Nog* cDNA by retrovirus infection together with RasV12 resulted in its overexpression and escape from senescence (Figure 5B–5D).

To clarify whether *Nog* at the physiological expression level could inhibit cellular senescence, *Nog*-transgenic (Nog-Tg) mice under Krt19 promoter [27] was used next, since the transgene was expected not to be modified with *de novo* H3K27me3. *Krt19* was expressed in MEFp2 at much higher level compared to brain and testis, confirming that *Krt19* promoter is active in MEF (Figure S9). Nog-Tg female mouse was crossed with C57B6, to establish and pool Tg(-) and Tg(+) MEFs from embryos of the same mother. Tg(+) MEF showed *Nog* expression at similar level to wild type MEFp2 and Tg(-) MEF (Figure 5E). While Tg(-) MEF showed *Nog* repression by RasV12 infection similarly to wild type MEF, Tg(+) MEF did not show *Nog* repression by RasV12 infection and showed continual growth faster than Tg(-) MEF (Figure 5E–5G). These indicated that *Nog* repression was also important in *Ras*-induced senescence.

### No detection of DNA methylation alteration

**Figure 6:**
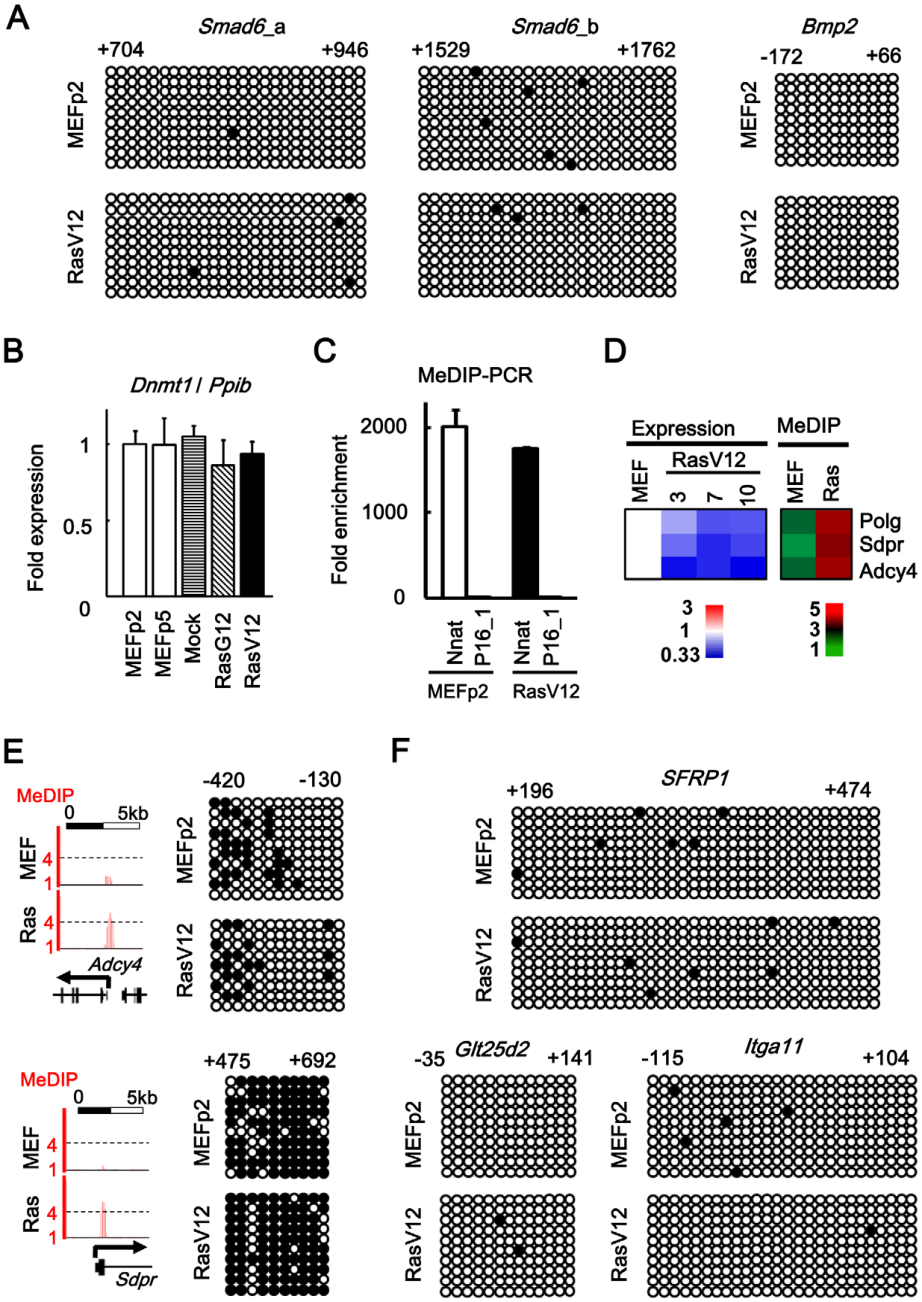
No detection of DNA methylation alteration. (*A*) Bisulfite sequencing of 5′ regions of *Smad6* and *Bmp2*. Positions were shown with TSS regarded as +1. *Open circle*, unmethylated CpG site. *Closed circle*, methylated CpG site. Nine to 10 clones were analyzed in each region, and aligned vertically; 10 – 36 CpG sites within the analyzed regions were aligned horizontally. Since H3K27me3 mark in *Smad6* was stretched towards exon 1 and intron 1 (Figure 3A), two regions (*a* and *b*) were analyzed for Smad6. There was no DNA methylation alteration in *Smad6* and *Bmp2*. (B) Real-time PCR for Dnmt1. Dnmt1 expression level was not altered in Ras-induced senescence, or during passages. (*C*) Validation of enrichment of methylated DNA in MeDIP. MeDIP-PCR was performed for 5′ regions of *Nnat* and *p16* (region 1 in Figure 1A), and fold enrichment relative to *p16* was shown. *Nnat* is an imprinted gene and a positive control for DNA methylation(+) region, and enrichment of methylated DNA in MeDIP was validated. (*D*) Analysis of MeDIP-seq. When analyzing ±1 kb of TSS of 20,232 genes, only 3 genes showed increase of MeDIP status from <2 reads per million reads in MEFp2 to >4 reads per million reads in RasV12 cells. (*E*) Validation of MeDIP-seq result. Among the three candidate methylated genes, *Adcy4* and *Sdpr* were chosen for validation by bisulfite sequencing. The methylation statuses of these genes, however, were not altered in Ras-induced senescence. (*F*) Bisulfite sequencing for 5′ regions of other genes. Among genes showing slight increase of MeDIP status, bisulfite sequencing was performed for five chosen genes: *Sfrp1* (from 0.7 reads in MEFp2 to 1.6 reads in RasV12 cells), *Glt25d2* (from 0.9 to 2.5), *Itga11* (0.6 to 2.7), *Shisa2* (0.6 to 2.0), and *Gypc* (0.8 to 1.9). *Sfrp1*, *Glt25d2*, and *Itga11* were representatively shown. These five genes were unmethylated in both MEFp2 and RasV12 cells.

It was reported that oncogenic Ras induces DNA methylation-mediated epigenetic inactivation in NIH3T3 cells [28], and that EZH2 directly controls DNA methylation [29], [30]. We therefore performed bisulfite sequencing to analyze DNA methylation statuses of 5′ regions of Smad6 and Bmp2 where increase or decrease of Ezh2 was confirmed (Figure 4D, Figure S6). There was no methylation alteration of these regions in RasV12 cells compared to MEFp2 (Figure 6A). Also, Dnmt1 expression level was not altered during *Ras*-induced senescence (Figure 6B). To gain insight whether oncogenic Ras induces DNA methylation-mediated inactivation in MEF on genome-wide scale, we performed methylated DNA immunoprecipitation (MeDIP)-seq in MEFp2 and RasV12 cells (Figure 6C, 6D). Although MeDIP is reported to be not accurate to detect DNA methylation in low-CpG regions, it is powerful screening method to detect candidate methylation regions in high-CpG regions, e.g. promoter CpG islands [22], [23], [31]. Increase of methylation was detected only in three candidate genes, and the increase was considered as a noise in genome-wide analysis because the increase was not validated by bisulfite sequencing (Figure 6D, 6E). Bisulfite sequencing was performed for five more genes which showed slight increase of methylation in MeDIP-seq, but there was no methylation alteration in RasV12 cells compared to MEFp2 (Figure 6F).

### *Ras*-induced senescence of human fibroblast IMR90

Human fibroblast IMR90 was infected with RasV12 retrovirus (RasV12-IMR90 cells). It was confirmed by SA-βgal staining on day 7 that cells fell into premature senescence (Figure S10A). Real-time RT-PCR showed that *BMP2* expression was markedly increased to 145-fold in RasV12-IMR90 cells, while *SMAD6* and *NOG* expressions were decreased to 0.32-fold and 0.15-fold, respectively (Figure S10B). *Nog* was introduced in IMR90 by retroviral infection with RasV12, and *Nog*-induced RasV12-IMR90 cells showed continual cellular growth (Figure S10C), suggesting that BMP2-SMAD1 is also an effector program in human fibroblasts.

### Downstream target genes of Bmp2-Smad1 signal analyzed by ChIP-sequencing

Since *Bmp2* upregulation, *Smad6* repression, and *Nog* repression were shown to contribute to *Ras*-induced senescence, downstream target genes of Bmp2-Smad1 signal are further analyzed on genome-wide scale.

**Figure 7:**
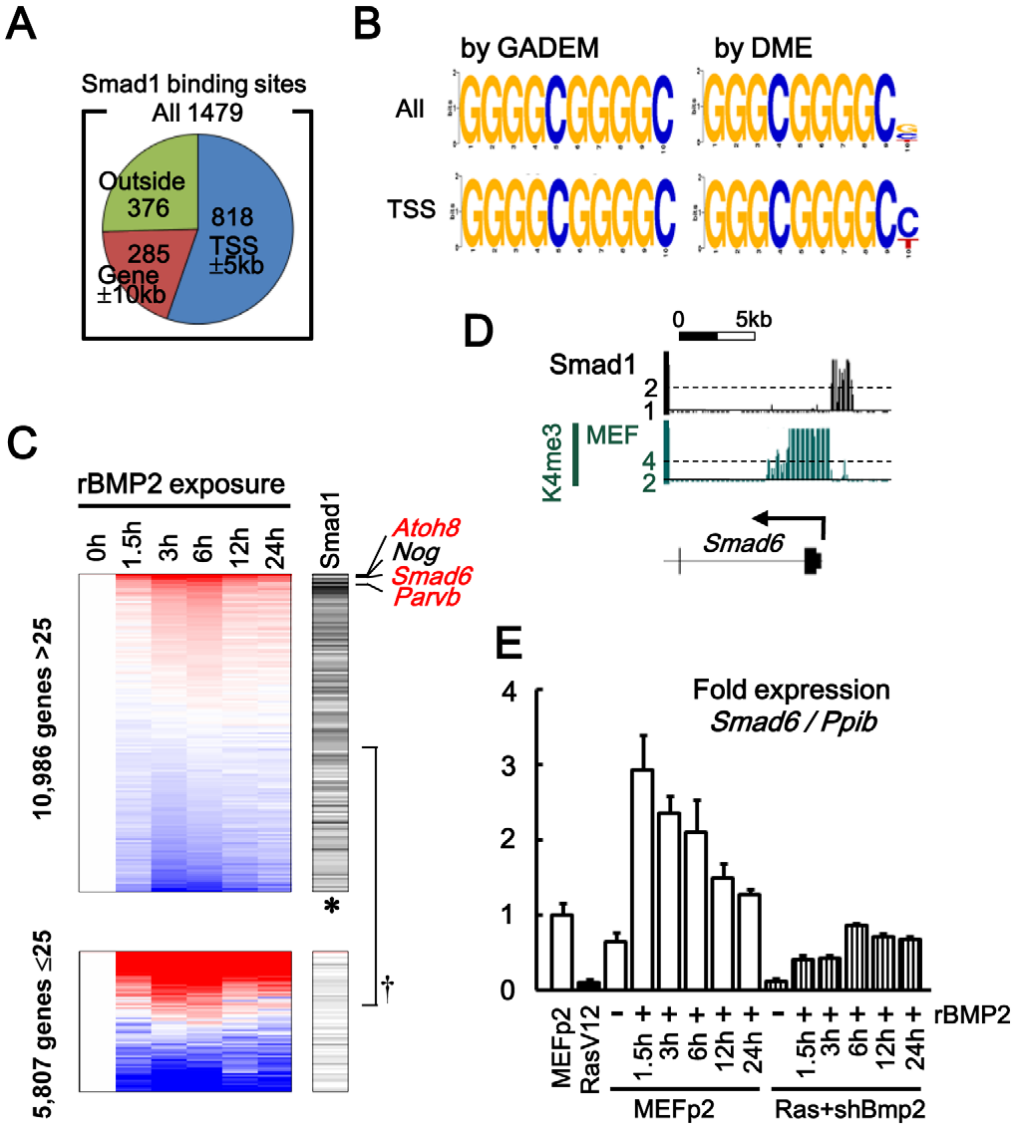
Smad1 targets in MEF, analyzed by ChIP-sequencing. (*A*) Among 1,479 Smad1 binding sites identified, 1,103 sites (75%, *blue* and *orange*) were located within 10 kb from 20,232 RefSeq genes, and 818 sites (55%, *blue*) were within 5 kb from their TSS. (*B*) GGGGCGGGGC was obtained as an enriched motif by GADEM [32], in Smad1 binding region within both whole genomic (*All*, ln(E-value) = −279.2) and TSS regions (*TSS*, ln(E-value) = −115.8). Very similar motifs were obtained by DME [33]. (See also Figures S9 and S10) (*C*) Correlation of Smad1 binding to gene upregulation. *Left*, expression levels in MEF at 0–24 hours after rBMP2 exposure. Genes were sorted by fold-expression level between 0 h and the mean of 3 h and 6 h. *Right*, 838 genes possessing Smad1 binding site in TSS region (*black bars*). *Upper panel*, genes with maximum GeneChip score during 24 h >25 (i.e. active genes). *Lower panel*, genes with maximum GeneChip score <25. Smad1 binding was significantly enriched in the upper panel of active genes (^†^P = 2×10^−57^, Fisher’s exact test), especially upward within the upper panel (thus genes upregulated by rBMP exposure, *P = 2×10^−11^, Kolmogorov-Smirnov test). Most upregulted genes included *Atoh8*, *Smad6*, *Parvb* (*red*, Smad1 target), and *Nog* (*black*, non-Smad1-target). (*D*) Smad1 binding site around Smad6 TSS. (*E*) Real-time RT-PCR showing *Smad6* upregulation in MEF by rBMP2 exposure. In *Bmp2*-knocked-down RasV12 cells, *Smad6* was suppressed lower than the level in MEFp2 even when exposed to rBMP2.

Smad1 binding sites in MEF were analyzed by exposing MEF to rBMP and ChIP-sequencing using anti-Smad1 antibody (Figure 7A and Figure S11). Smad1 mostly bound to gene regions; 1,103 (75%) out of 1,479 Smad1 binding sites were located within 10 kb from 20,232 RefSeq genes, and 818 sites (55%) were within 5 kb from their TSS. Using GADEM (http://www.niehs.nih.gov/research/resources/software/gadem/) [32], GGGGCGGGGC was extracted as highly enriched motif within Smad1 binding region in both whole genomic and TSS regions (Figure 6B, Figure S12). Using DME (http://rulai.cshl.edu/dme/) [33], it was confirmed that very similar motifs e.g. GGGCGGGGC (Figure 7B) or GGGGCGGGGM (Figure S13) were enriched. This was in good agreement with the canonical SMAD1-bound GC-rich elements [26], [34], [35] and the previous report that the sequence GGCGGGGC was enriched within Smad1/5 binding regions in ES cells and pulled down SMAD proteins [36]. Genes with Smad1 binding site at TSS regions were significantly enriched in active genes in MEF, especially in genes upregulated by rBMP exposure (Figure 7C), suggesting that Smad1 binding correlates to gene upregulation. Smad1 target genes upregulated most by rBMP exposure included *Smad6*, which was upregulated by 4.5-fold in MEF (Figure 7D, 7E). These indicated that Bmp2/Smad1 signal in MEF could be controlled by negative feedback through Smad1 regulation on *Smad6*.

### Smad1 target genes repressed or activated during senescence

**Figure 8:**
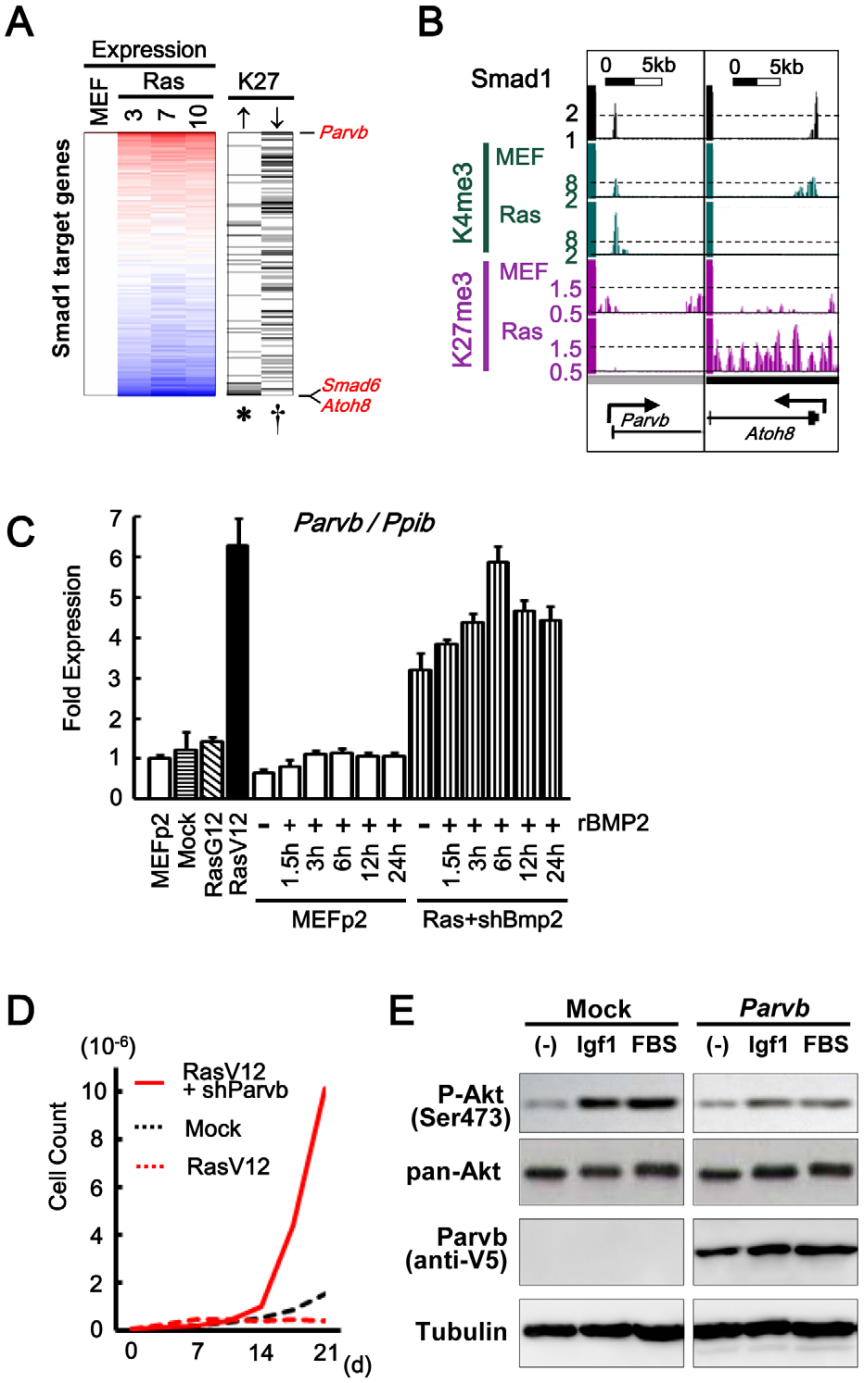
Smad1 target genes repressed and activated in senescence. (*A*) Repression of Smad1 target genes by H3K27me3. 838 Smad1 target genes in MEF were sorted by fold expression change between MEFp2 and mean of RasV12 day3, 7, and 10 (*Expression column*). 50 genes showed increase of H3K27me3 reads in RasV12 by 0.4 or more (↑*column*), and were significantly enriched downward (*P = 0.004, Kolmogorov-Smirnov test), i.e. repressed. Genes were listed in Table S5. 581 genes with no increase of H3K27me3 reads (not shown), or 156 genes showing decrease of H3K27me3 reads by 0.4 or more (↓*column*), were significantly enriched upward (P = 0.01, and ^†^P = 0.004, respectively), i.e. upregulated. The former included *Atoh8* and *Smad6*, and the latter included *Parvb*. (*B*) Maps around TSS of *Parvb* and *Atoh8*. (*C*) Real-time RT-PCR of *Parvb*. *Parvb* expression was increased in RasV12 cells, or in exposure to rBMP2 in MEF. Compared to the level in RasV12 cells, *Parvb* expression level was approximately half (0.51±0.07 fold) when *Bmp2* was knocked down (*Ras+shBmp2*), and increased to the similar level (0.94±0.06 fold) when exposed to rBMP. (*D*) Growth curve. *Parvb*-knocked-down RasV12 cells showed continual growth. (*E*) Western blot analysis of Akt phosphorylation. Parvb with C-terminal V5 was detected using anti-V5 antibody. MEF’s with *Parvb* cDNA introduction and mock infection were stimulated by 50 ng/mL Igf1 or 10% fetal bovine serum (*FBS*). Akt was phosphorylated by Igf1 or serum stimulation in Mock cells, which showed very low *Parvb* expression (See also Figure S14A). Akt phosphorylation by Igf1 or serum stimulation was decreased in *Parvb*-introduced cells.

However, *Smad6* was repressed in RasV12 cells by H3K27me3, so when *Bmp2*-knocked-down RasV12 cells was exposed to rBMP2, Smad6 level was still suppressed lower than the level in MEFp2 (Figure 7E). Smad1 target genes repressed in RasV12 cells were not limited to Smad6. H3K27me3 gain during *Ras*-induced senescence was detected in 50 Smad1 target genes, which were enriched in genes repressed in RasV12 cells, e.g. *Atoh8*. *Atoh8* was highly upregulated in BMP2 exposure, but repressed in RasV12 cells with decrease of H3K4me3 from 8.7 to 1.8 and increase of H3K27me3 mark from 1.0 to 1.8 (Figure 7C, Figure 8A and 8B. Gene list is available in Table S5). It was reported that *Atoh8* was, like *Id1*, suggested to be a direct target of BMP-SMAD signal [37].

On the contrary, Smad1 target genes without increased repressive mark were shown to keep upregulation. Among 838 Smad1 target genes, 581 with no increase of H3K27me3, or 156 showing decrease of H3K27me3, were significantly enriched in genes upregulated in RasV12 cells (P = 0.01 and P = 0.004, respectively, Figure 8A). If Bmp2/Smad1 signal is critical in senescence, the most upregulated target genes are expected to include genes with growth suppressor function. To choose such candidate genes, the most upregulated target genes were screened using promoter methylation data of our previous methylated DNA-immunoprecipitation (MeDIP)-chip analyses of human cancer cells [22], [23] (Table S6), since such genes may possibly be frequently inactivated in human cancer. The most upregulated targets then included *Parvb*, which showed promoter methylation in human cancer cell lines HCT116 and DLD1 (Table S6). When MEF senesced, *Parvb* showed increase of H3K4me3 from 8.6 to 16.8, and decrease of H3K27me3 from 1.0 to 0.6 (Figure 8B). Real-time RT-PCR validated increase of *Parvb* expression in RasV12 cells, and also when exposed to rBMP2 (Figure 8C). When *Parvb* was knocked down to 0.05-fold by shRNA, SA-βgal(+) cells were partially decreased and cells showed continual growth (Figure 8D and Figure S14). Western blot analysis showed decrease of Akt phosphorylation in exposure to a growth factor or serum when Parvb with C-terminal V5 tag was introduced in MEF (Figure 8E).

## Discussion

In this study, we examined H3K4me3 and H3K27me3 marks for genome-wide analysis of epigenomic changes, revealing that activation of Bmp2-Smad1 signal is important in *Ras*-induced senescence and it is regulated by dynamic epigenomic alteration in coordinated manner. Different from *p16*, H3K4me3 and H3K27me3 marks on *Bmp2* was not altered during passage in cell culture, but specifically altered in RasV12 cells to induce its marked upregulation, leading to Smad1/5/8 phosphorylation and cellular senescence. Decrease of *Ezh2* and increase of *Jmjd3* were detected in RasV12 cells at similar levels to MEFp5, Mock cells and RasG12 cells. This may contribute to partial increase of *p16* expression in MEFp5, Mock cells and RasG12 cells, and partial decrease of H3M27me3 mark on *p16* in stress-induced senescence during passages as reported [16], [18]. However, the alterations on *p16* were more markedly detected in RasV12 cells, and the alterations on *Bmp2* and *Smad6* were specifically detected in *Ras*-induced senescence and did not occur during passages. It is noteworthy that de novo formation of H3K27me3 occurs on *Smad6* in RasV12 cells in spite of general decrease of *Ezh2* and increase of *Jmjd3*.

The mechanism how these epigenetic regulations are programmed is largely unknown, but one possible answer might be non-coding RNA [38], [39]. PRC2 was reported to be recruited in *trans* to its target gene by virtue of its association with *HOTAIR*, a 2.2 kb non-coding RNA in the HOXC locus [40]. Oncogenic Ras inhibited expression of *ANRIL* (antisense non-coding RNA in the INK4 locus); *ANRIL* showed binding to CBX7 within PRC1 and SUZ12 in PRC2, and was important in repressing the protein-coding genes of *INK4b/ARF/INK4a* locus in *cis* to regulate senescence [41], [42]. Ezh2 recruitment was increased in *Smad6*, and decreased in *Bmp2* and *p16* (Figure 1D, Figure 4D, Figure S6). It would be interesting to analyze whether any non-coding RNAs recruit PRC to *Smad6* and *Bmp2* in *cis* or *trans*, and their expression alterations contribute to epigenetic alterations of these genes during *Ras*-induced senescence.

Gene repressions by other epigenetic mechanism than Polycomb, such as H3K9 methylation, would be interesting to be analyzed next. Human fibroblasts in senescence are reported to suppress DNA damage response by forming heterochromatic foci, where regions with methylated H3K9 gathered [12]. Amplification of SETDB1, a methyltransferase for H3K9, was recently reported to play an accelerating role in melanoma onset [13], while knockout of Suv39h1, another histone methyltransferase for H3K9, caused escape from senescence of lymphocytes [43], suggesting necessity of adequate control of H3K9 methylation. Genome-wide analyses of methylated H3K9 and other epigenomic marks as well would be helpful to obtain the whole picture of epigenomic alteration and its importance in senescence.

As for DNA methylation, it was reported that oncogenic Ras induces DNA methylation-mediated epigenetic inactivation in NIH3T3 cells and that 28 responsible genes including DNMT1 are required for the methylation [28]. DNA methylation statuses at 5′ regions of *Smad6* and *Bmp2* were not altered, however, indicating that expression changes of these genes during senescence were not due to DNA methylation. Dnmt1 level was not altered in RasV12 cells, either. Increase of methylation was detected only in three candidate genes by MeDIP-seq analysis, and the increase was considered as a noise in genome-wide analysis because the increase was not validated by bisulfite sequencing. Five more genes were chosen for bisulfite sequencing, because *Sfrp1* was reported to be methylated by oncogenic Ras in NIH3T3[28], and four other genes were chosen randomly from genes with slight increase of methylation in MeDIP-seq. There was no methylation alteration in RasV12 cells compared to MEFp2, either. Although MeDIP is not accurate to detect DNA methylation in low-CpG regions [22], [31], it was suggested that DNA methylation unlikely occurs in Ras-induced senescence, at least high-CpG regions e.g. promoter CpG islands. The discrepancy between the previous report of NIH3T3 and our MEF result may be because MEF falls into cellular arrest by oncogenic stress and there might be no time enough to induce DNA methylation alteration. In NIH3T3, cells transform by oncogenic Ras, and may have time enough to acquire DNA methylation during continuing proliferation. Or, two independent cells, NIH3T3 (ATCC #CRL-1658) and *K-ras*-transformed NIH3T3 (ATCC #CRL-6361), were compared in the previous NIH3T3 study [28], so the result might be different if one NIH3T3 clone is analyzed at time courses before and after induction of activated *Ras*.

As for BMP-SMAD signals, utilization of four BMP type 1 receptors depends on BMP ligands; BMP2 and BMP4 utilize BMPR1A and BMPR1B, BMP6 and BMP7 bind principally to ACVR1, and BMP9 is a ligand for ACVRL1 and ACVR1 [26]. We reported that Smad6 inhibited BMPR1A/BMPR1B preferentially to ACVR1/ACVRL1, and inhibited BMP2-induced Smad1/5 phosphorylation more prominently than BMP6-induced Smad1/5 phosphorylation [44]. This is in agreement with the current results that Smad6 could cause decreased phosphorylation of Smad1/5 and escape from senescence, and that coordination of *Bmp2* upregulation and *Smad6* repression was critical in *Ras*-induced senescence.

**Figure 9:**
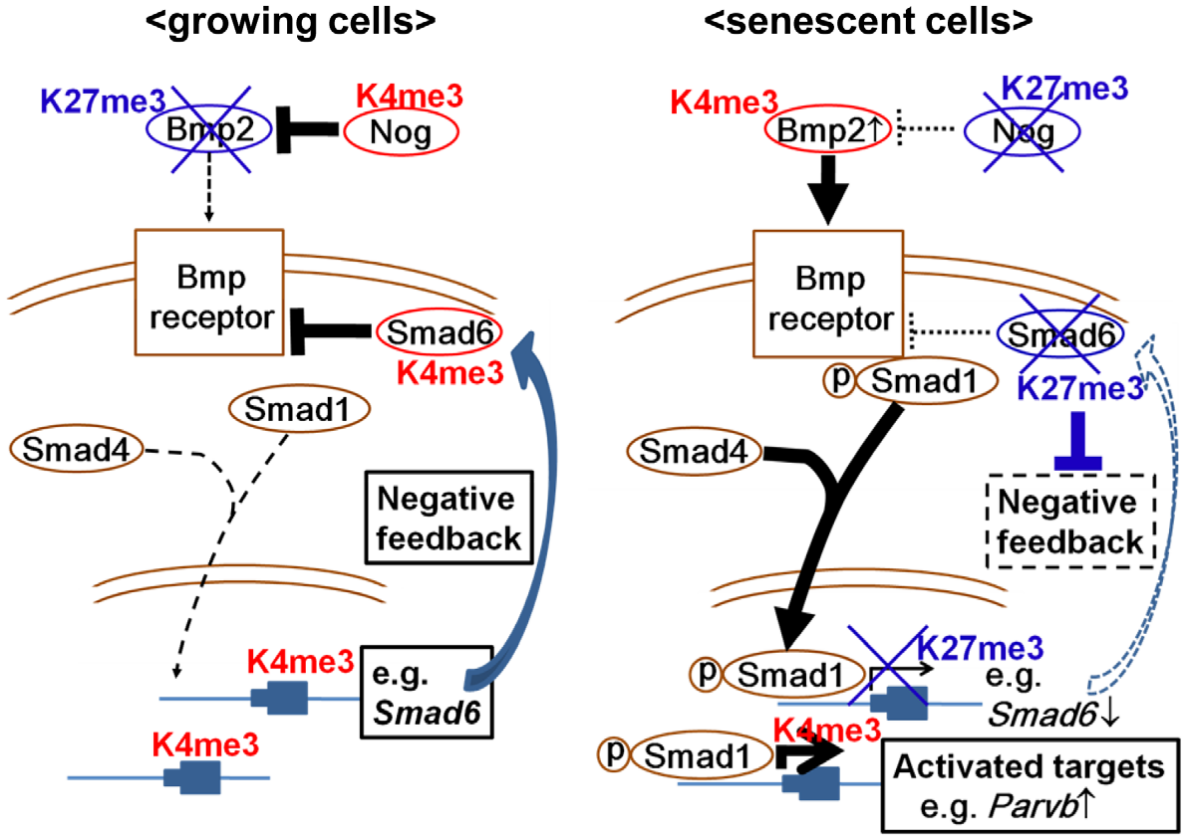
Schema of epigenetic regulation of Bmp2/Smad1 signal. Negative factors for senescence were inactivated selectively and epigenetically, and positive factors/signals for senescence were activated selectively and epigenetically.

Our genome-wide analysis showed that *Smad6* was a Smad1 target gene that could be highly upregulated by exposure to BMP2, but strongly repressed in RasV12 cells with *de novo* H3K27me3 mark. Previous reports showed that BMP-activated Smad1/5 activates *Smad6* expression through interaction with the *Smad6* promoter [45], [46]. These suggested that *Smad6* repression with *de novo* H3K27 methylation blocks negative feedback loop to sustain the effect of upregulated *Bmp2*, *i.e.* activation of Bmp2-Smad1 signal in *Ras*-induced senescence. In other words, dynamic H3K27me3 alteration is suggested to repress selectively the genes which could negatively control senescent signal, and to activate selectively genes which could positively affect senescent signal (Figure 9). In fact, another BMP-SMAD inhibitor, *Nog*, was also repressed by increased H3K27me3 mark. While ChIP-seq analysis did not show Smad1 binding site around *Nog* TSS, *Nog* was also highly upregulated by rBMP2 exposure (Figure 6C) and repressed by increased H3K27me3 mark in RasV12 cells (Figure 5A, 5B). This might suggest that *Nog* repression could also be a disruption of negative feedback loop, though *Nog* is not a direct downstream target of Bmp2-Smad1.

*Parvb*, which possessed Smad1 binding site around its TSS, was upregulated in exposure to BMP2 or in RasV12 cells, and its knock down lead to escape from senescence. While PARVA was reported to bind to integrin-linked kinase (ILK) and play a critical role in cell survival by promoting membrane recruitment of Akt and its activation by phosphorylation, PARVB was reported to compete PARVA in binding to ILK and reverse its oncogenic effect by repressing ILK kinase activity [47], [48]. As *PARVB* introduction was reported to suppress cellular growth of breast cancer cells with decreased Akt phosphorylation [49], [50], *Parvb* introduction in MEF also decreased phosphorylation of Akt in exposure to a growth factor or serum (Figure 7E). It was suggested that *Parvb* might be one of Bmp2-Smad1 target genes playing a positive role in growth inhibition, at least partly, and selectively and effectively activated through simultaneous inactivation of negative regulators. We chose *Parvb* on the assumption that candidate genes downstream of BMP-SMAD might be inactivated by DNA methylation in full-blown cancers, but other downstream genes that were not methylation target in analyzed cancer cell lines might also play a positive role in senescence.

Aberrations in BMP-SMAD signal have been frequently reported in human cancer. Juvenile polyposis syndrome, an inherited syndrome with high risk of colorectal cancer, is caused by germline mutation of *BMPR1A* or *SMAD4* [51], and importance of BMP signal is supported by its mouse model with transgenic *Nog* expression or with Bmpr1a inactivation [52], [53]. BMP2 expression was lost in microadenoma of familial adenomatous polyposis, while BMP2 was expressed in mature colonic epithelial cells, promoting apoptosis and differentiation and inhibiting proliferation [54]. Inactivation of BMPR1A, BMPR2, and SMAD4 was frequently observed in sporadic colorectal cancer, correlating to loss of Smad1/5/8 phosphorylation [55]. Colon epithelial polyps were developed even by alteration of BMP pathway in the stromal microenvironment, using mice with conditional inactivation of Bmpr2 in the stroma [56]. About prognosis, Smad6 expression was reported to be elevated in 40% of non-small cell lung cancer, and correlated to poorer outcome [57]. BMP2 upregulation was reported in senescence of other cell types, such as vascular smooth muscle cells [58]. Considering frequent RAS gene mutation in cancer, e.g. colon (∼40%) and non-small cell lung cancers (∼30%) [59], further experiments are to be performed to clarify which cell types Bmp2-Smad1 signal is critical in oncogene-induced senescence, and whether Bmp2-Smad1 signal and its target genes are disrupted in cancer with association to oncogene mutation.

## Materials and Methods

Full information of Material and Methods is also described in Supporting Materials and Methods (Text S1).

### Cells and viral infection

MEF was established from 13.5 embryonic day embryos of C57/B6 as reported [60]. After cells were passed twice (MEFp2), cells were infected with retroviruses for 48 hours. Then cells were exposed to 4 µg/mL puromycin for selection during days 0–3, and were passed on days 3, 7, and 10. Human fibroblast IMR90 (JCRB9054) was purchased from Health Science Research Resources Bank (Osaka, Japan), and 2 µg/mL puromycin were used for selection after retrovirus infection. Total RNA was collected using TRIzol (Invitrogen, Carlsbad, CA). This study was certified by Animal Ethics Committee in Tokyo University.

### MEF of Nog-trangenic (Nog-Tg) mice

Nog-Tg mice using keratin 19 gene promoter and mouse Nog cDNA were previously established [27], and were crossed with wild type C57/B6 mice five times to obtain C57/B6 background. Nog-Tg female mouse was crossed with C57B6, and Tg(-) and Tg(+) MEFs were established from 13.5 embryonic day embryos of the same mother. Each embryo was minced separately, and Tg(-) and Tg(+) MEFs were pooled after genotyping each MEF, and used for experiments.

### Retroviral vectors

Retroviral vectors for Ras was constructed by cloning cDNAs for wild type HRAS (RasG12) and mutated HRAS (RasV12) by reverse-transcription PCR products from HMEC and SK-BR3 cell RNA, respectively, with N-terminal FLAG tag into pMX vector that contains puromycin resistance gene (a kind gift from T. Kitamura). Mock pMX vector (Mock), and vectors containing RasG12 and oncogenic RasV12 were transfected into plat-E packaging cells (a kind gift from T. Kitamura) using FuGENE 6 Transfection Reagent (Roche, Germany) to prepare retroviruses. Smad6 cDNA with N-terminal 6x Myc tag, Nog cDNA with C-terminal V5 tag, and Parvb cDNA with C-terminal V5 tag were also cloned into pMX vector. To knock down Bmp2 or Parvb, double strand oligonucleotide DNA to express small hairpin RNA against *Bmp2* (shBmp2) or *Parvb* (shParvb), respectively, was cloned into RNAi-Ready pSIREN-RetroQ Vector (Clontech, CA). Viral packaging for Smad6, Nog, shBmp2 and shParvb retrovirus vectors was also done using plat-E cells. Retroviruses of RasV12 and Nog for human fibroblast were prepared using Retrovirus Packaging Kit Ampho (#6161, TaKaRa Bio Inc, Shiga, Japan).

### Expression array analysis

For genome-wide transcription analysis, GeneChip Mouse Genome 430 2.0 Array (Affymetrix) was used as described [61]. The GeneChip data were analyzed using the Affymetrix GeneChip Operating Software v1.3 by MAS5 algorithms, to obtain signal value (GeneChip score) for each probe. For global normalization, the average signal in an array was made equal to 100. Gene annotation enrichment analysis was done at DAVID Bioinformatics Resources (http://david.abcc.ncifcrf.gov/). Array data is available at GEO datasets (#GSE18125).

### ChIP, MeDIP, and sequencing

MEFp2 and infected cells at day 10 were cross-linked with 1% formaldehyde for 10 min and were prepared for ChIP. ChIP using anti-H3K4me3 (ab8580, abcam, rabbit polyclonal), H3K27me3 (07–142, Upstate, rabbit polyclonal), or Ezh2 (#39103, Active Motif, rabbit polyclonal) antibody was performed as described previously [62]. For ChIP using anti-Smad1 antibody (BioMatrix, mouse monoclonal), MEFp2 cells were starved for 16 hours and exposed to rBMP2 protein (#355-BM, R&D systems) at 25 ng/mL in serum-free medium for 1.5 hours. Cells were cross-linked with 1 mM Disuccinimidyl Glutarate (Thermo Scientific, Rockford, IL) for 20 min and 1% formalin for 10 min, and ChIP was performed similarly.

For MeDIP, genomic DNA of MEFp2 and RasV12 cells was fragmented by sonication, and immunoprecipitated by anti 5-methylcytocine monoclonal antibody (kindly supplied by Dr. K. Watanabe, Toray Research Center, Inc.), as we previously reported[22], [23], [63]. MeDIPed sample and Input sample underwent MeDIP-PCR to check enrichment of methylated regions in MeDIPed sample.

Sample preparation for ChIP- and MeDIP-sequencing was performed according to the manufacturer's instructions (Illumina), and sequencing was performed using Solexa Genome Analyzer II [61]. 36-bp single end reads were mapped to the NCBI Build #36 (UCSC mm8) reference mouse genome, using the Illumina pipeline software v1.4. The numbers of uniquely mapped reads for MEFp2 were 10,845,082 (H3K4me3), 11,519,151 (H3K27me3), 9,663,324 (DNA methylation) and 5,688,804 (Input), those for RasV12 cells were 13,246,871 (H3K4me3), 9,894,241 (H3K27me3), 11,319,506 (DNA methylation) and 6,126,206 (Input), and that for Smad1 ChIP-sequencing was 9,417,307. Window sizes of 300 bp for H3K4me3, 500 bp for H3K27me3, 500 bp for DNA methylation and 300 bp for Smad1, were used to calculate the number of mapped reads per million reads at the center of the window. Sequencing data is also available (#GSE18125).

### Immunoblot analysis

Aliquots of protein were subjected to SDS/PAGE and were transferred to nitrocellulose, and the resulting immunoblots were visualized using Amersham ECL Plus (GE Healthcare) and LAS-3000 (Fujifilm, Japan).

### Cellular immunofluorescence

Phosphorylated Smad1/5/8 was detected using antibody against phospho-Smad1/5/8 (Cell Signaling) as primary antibody, and green-fluorescent Alexa Fluor 488 dye-labeled anti-rabbit antibody (Invitrogen) as secondary antibody. RasV12 with N-terminal FLAG tag and Smad6 with N-terminal Myc tag were detected using antibody against FLAG (F7425, Sigma, rabbit polyclonal) and Myc (9E10, Santa Cruz, mouse monoclonal) as primary antibody, respectively, and Alexa Fluor 594 anti-rabbit antibody and Alexa Fluor 488 anti-mouse antibody (Invitrogen) as secondary antibody. Photographs were taken with Biozero BZ-8100 (KEYENCE, Osaka, Japan).

### Senescence-associated β-galactosidase (SA-βgal) analysis

MEFp2 and infected MEFs on day 10, and infected IMR90 on day 7 underwent SA-βgal staining as previously described [64].

### Growth curve

Infected MEFs were counted on days 3, 7, 10, 14, 17, 21 using Countess automated cell counter (Invitrogen) and seeded at density of 1×10^5^ cells/6-cm dish for every passage. Infected IMR90 were counted on days 4, 8, 12 and 16 similarly, and seeded at density of 2.5×10^5^ cells/6-cm dish. Mean number of three dishes was calculated and used to draw growth curve.

### Quantitative real-time RT-PCR, ChIP-PCR, and MeDIP-PCR

Real-time PCR was performed using iCycler Thermal Cycler (Bio-Rad Laboratories) as previously described [65]. The experiment was triplicated and mean and standard error were calculated and shown. Primer information is in Tables S7 and S8.

### Bisulfite treatment and bisulfite sequencing

DNA methylation status was analyzed by bisulfite sequencing as previously described [65]. Briefly, 500 ng of genomic DNA of MEFp2 and RasV12 cells underwent bisulfite treatment, and were finally suspended in 20 µL of distilled water. For bisulfite sequencing, 1 µl was used as a template for PCR with primers common for methylated and unmethylated DNA sequences. The primers and PCR conditions are available at Table S9. PCR products were cloned into pGEM-T Easy vector (Promega), and 9–10 clones each were cycle-sequenced using T7 and Sp6 primers.

## Supporting Information

Figure S1 Schema of experiments. (*A*) Candidate factors to induce senescence are to be identified from genes upregulated, with loss of repressive epigenetic mark and with gain of active mark. (*B*) Candidate inhibitors to senescence are to be identified from genes downregulated, with gain of repressive epigenetic mark and with loss of active mark.
(TIF)

Figure S2 Induction of activated *Ras* by retrovirus infection. (*A*) Western blot analysis of Ras protein. Expression of Ras protein with N-terminal FLAG tag was confirmed by western blot analysis using anti-FLAG antibody. (*B*) Count of senescence-associated β-galactosidase (SA-βgal)-positive cells. MEF cells at passage 2 (MEFp2, *open box*) rarely showed SA-βgal(+) cells. MEF cells at passage 5 without virus infection (MEFp5, *open box*) showed slight increase in number of SA-βgal(+) cells, and two control cells infected with mock vector (Mock cells, *horizontal-striped box*) and wild type Ras (RasG12 cells, *crosshatched box*) showed similar level of SA-βgal staining, indicating stress-induced senescence during passages. The stress response is a consequence of the high oxygen levels that inflict oxidative damage to the cells resulting in senescence [16]. RasV12-infected cells (RasV12 cells, *closed box*) showed marked increase in number of SA-βgal(+) cells. Representatives were mean and standard error in three repeated experiments.
(TIF)

Figure S3 Distribution of epigenetic marks. (*A*) Distribution of H3K4me3 and H3K27me3 marks around TSS was shown by the number of mapped Solexa reads per million reads within a window size of 300 bp and 500 bp, respectively. The distribution was similar between MEFp2 and RasV12. The peak of H3K4me3 mark was detected at +247 bp for MEFp2, and at +312 bp for RasV12, and distribution was rather narrow. The peak of H3K27me3 mark was detected at +698 bp for MEFp2, and at +420 bp for RasV12, and distribution was rather wide than H3K4me3. (*B*) Epigenetic statuses of H3K4me3 and H3K27me3 for each gene were decided by the maximum number of mapped reads per million reads in a window size of 300 bp and 500 bp, respectively, within 2 kb ± TSS of each gene, and distribution of the epigenetic status of 20,232 genes was shown by heat map. The number of H3K27me3(+) genes was generally decreased.
(TIF)

Figure S4 Expression analysis of *Ezh2* and *Jmjd3*. Real-time RT-PCR was performed, and normalized to *Ppib*, and relative expression levels compared to MEFp2 was shown. *Ezh2* expression level was decreased in RasV12-induced senescence, but similar downregulation was observed during three passages without viral infection, or with mock and RasG12 infection. Similarly, *Jmjd3* expression level was increased in RasV12-induced senescence, but similar upregulation was observed during three passages without viral infection, or with mock and RasG12 infection.
(TIF)

Figure S5 Relation between expression and histone marks (+/−). (*A*) For epigenetic status of H3K4me3, genes with >4 reads per million reads within a window size of 300 bp were regarded as H3K4me3(+), and genes with <3 reads were as H3K4me3(-). Mean and standard error of expression signal (GeneChip score) were shown. Markedly high expression was confirmed in H3K4me3(+) genes. (*B*) For epigenetic status of H3K27me3, genes with >1.5 reads per million reads within a window size of 500 bp were regarded as H3K27me3(+), and genes with <1 read were as H2K27me3(-). Markedly low expression was confirmed in H3K27me3(+) genes.
(TIF)

Figure S6 Decreased enrichment of Ezh2 around TSS of Bmp2. Quantitative ChIP-PCR was performed for ∼100 bp upstream (region *1*) and ∼300 bp downstream (region *2*) of *Bmp2* TSS (See Figure 3A), and shown by relative fold enrichment compared to *Actb* (Figure 1D). Decreased enrichment of Ezh2 around *Bmp2* TSS was shown (*P<0.05).
(TIF)

Figure S7 Cellular immunofuorescence for RasV12 and Smad6. RasV12 with N-terminal FLAG and Smad6 with N-terminal Myc were detected using anti-FLAG and anti-Myc antibodies. Simultaneous expression of RasV12 and Smad6 proteins in Smad6-introduced RasV12 cells was confirmed (See also Figure 4E).
(TIF)

Figure S8 Decreased SA-βgal(+) cells by Smad6 overexpression. Retrovirus of *Smad6* cDNA was infected with Mock or RasV12 retrovirus. *Smad6-*induced RasV12 cells showed decreased number of SA-βgal(+) cells compared to RasV12 cells (*black box*). Smad6 induction did not affect on Mock cells (*horizontally striped box*).
(TIF)

Figure S9 Expression of Krt19 in MEF. (*A*) RT-PCR showed that MEFp2 showed expression of *Krt19* in MEFp2 and not in brain and testis. (*B*) Real-time RT-PCR showed that *Krt19* expression level in MEFp2 was much higher than brain and testis.
(TIF)

Figure S10 *Ras*-induced senescence in human fibroblast IMR90. (*A*) IMR90 was infected with RasV12 retrovirus. In western blotting, expression of Ras protein with N-terminal FLAG tag on day 4 was detected using anti-FLAG antibody (*left*). SA-βgal staining on day 7 showed that RasV12-IMR90 cells fell into senescence (*right*). (*B*) Real-time RT-PCR showed that *BMP2* expression was markedly increased to 145-fold in RasV12-IMR90 cells, while *SMAD6* and *NOG* expressions were decreased to 0.32-fold and 0.15-fold, respectively. (*C*) *Nog*-induced RasV12-IMR90 cells showed continual cellular growth.
(TIF)

Figure S11 ChIP using anti-Smad1 antibody. (*A*) There was no Smad1 binding site detected around *Actb*, while a Smad1 binding site was detected at 1 kb upstream of Id1 TSS. o*pen squares*, regions for ChIP-PCR. (*B*) Smad1 binding at 1 kb upstream of Id1 TSS was validated by ChIP-PCR.
(TIF)

Figure S12 The motifs within Smad1 binding regions by GADEM. GADEM version1.3 (http://www.niehs.nih.gov/research/resources/software/gadem/index.cfm) was used to search for the motifs with default parameters except -posWt (Weight profile for positions on the sequence)  = 1, -pv (P-value cutoff)  = 0.00001, -em (Number of EM steps)  = 20, and -fullScan  = 1. The enriched sequences were drawn by STAMP (http://www.benoslab.pitt.edu/stamp/). Ln(E-value) and fold-enrichment to sites in background sequence were shown. 20 and 10 motifs were obtained in whole genomic region (*All*) and TSS regions (*TSS*), respectively. GGGGCGGGGC was commonly detected in both analyses.
(TIF)

Figure S13 The motifs within Smad1 binding regions by DME. To confirm the GADEM result (Figure S9), the motifs were searched for by another software DME2 (http://rulai.cshl.edu/dme/), using ZOOPS model with default parameters except -w (minimum desired motif width)  = 10 and -n (number of motifs to produce)  = 10. The results were drawn by STAMP, including the tree view at the bottom. It was confirmed that DNA sequences very similar to GGGGCGGGGC were enriched, such as GGGGCGGGGM and GGGCGGGGC.
(TIF)

Figure S14 *Parvb* in *Ras*-induced senescence. (*A*) Real-time RT-PCR showed that *Parvb* was knocked down by shRNA to 0.05-fold. (*B*) *Parvb*-knocked down RasV12 cells showed partially decreased number of SA-βgal(+) cells compared to RasV12 cells, though higher than Mock level.
(TIF)

Table S1 Significant terms with P<10^−10^ were listed. When there were less than five terms with P<10^−10^, top five terms with P<10^−5^ were listed for each category. * The term included *Bmp2*.
(DOC)

Table S2 Significant terms with P<10^−10^ were listed. When there were less than five terms with P<10^−10^, top five terms with P<10^−5^ were listed for each category. ^§^ The term included *Smad6*.
(DOC)

Table S3 H3K4me3 and H3K27me3 levels were shown by the maximum number of the mapped Solexa reads per million reads within a window size of 300 bp and 500 bp, respectively, for 2 kb around TSS. Expression levels were shown by GeneChip score. Secreted factors *Bmp2* and *Igfbp3* were shown to be highly upregulated with H3K27me3 loss and H3K4me3 gain.
(DOC)

Table S4 H3K4me3 and H3K27me3 levels were shown by the maximum number of the mapped Solexa reads per million reads within a window size of 300 bp and 500 bp, respectively, for 2 kb around TSS. Expression levels were shown by GeneChip score. Two Bmp2/Smad1 signal inhibitors, Smad6 and Nog, were included in the nine genes with H3K27me3 gain and H3K4me3 loss.
(DOC)

Table S5 Smad1 target genes were generally upregulated by Bmp2 stimulation in MEF (Figure 6), but genes with H3K27me3 increase e.g. *Smad6* and *Atoh8* were repressed in RasV12 cells. Smad1 target genes without H3K27me3 increase were correlated to upregulation (Figure 7 and Table S6).
(DOC)

Table S6 Smad1 target genes without H3K27me3 increase were correlated to upregulation (Figure 7). Among Smad1 target genes upregulated in RasV12 cells, top ranking 30 genes showing >3-fold upregulation were listed. None of these genes showed H3K27me3 increase >0.4. These genes were regarded as selectively upregulated Smad1 target genes, and expected to include genes with growth suppressor function. Such genes may perhaps be frequently inactivated in human cancer e.g. by promoter methylation. Among these genes, *Parvb* was chosen to be examined as *Parvb* expression was highly induced by BMP2 stimulation in MEF (Figure 6) and our previous methylated DNA immunopreccipitation (MeDIP)-chip analysis of human cancer cell lines [22], [23] showed PARVB promoter methylation in HCT116 and DLD1 (*the most right columns above*).
(DOC)

Table S7 *Ppib* and *PPIA* were used for normalization.
(DOC)

Table S8 Location of TSS was regarded as +1.
(DOC)

Table S9 Location of TSS was regarded as +1.
(DOC)

Text S1 Supporting Materials and Methods, and references for them, are available in Text S1.
(DOC)
