# Supplementary figures and images for: Activation of Bmp2-Smad1 Signal and Its Regulation by Coordinated Alteration of H3K27 Trimethylation in Ras-Induced Senescence
Source: PLoS Genet. 2011 Nov 3;7(11):e1002359. doi: 10.1371/journal.pgen.1002359 (PMC3207904; doi:10.1371/journal.pgen.1002359)

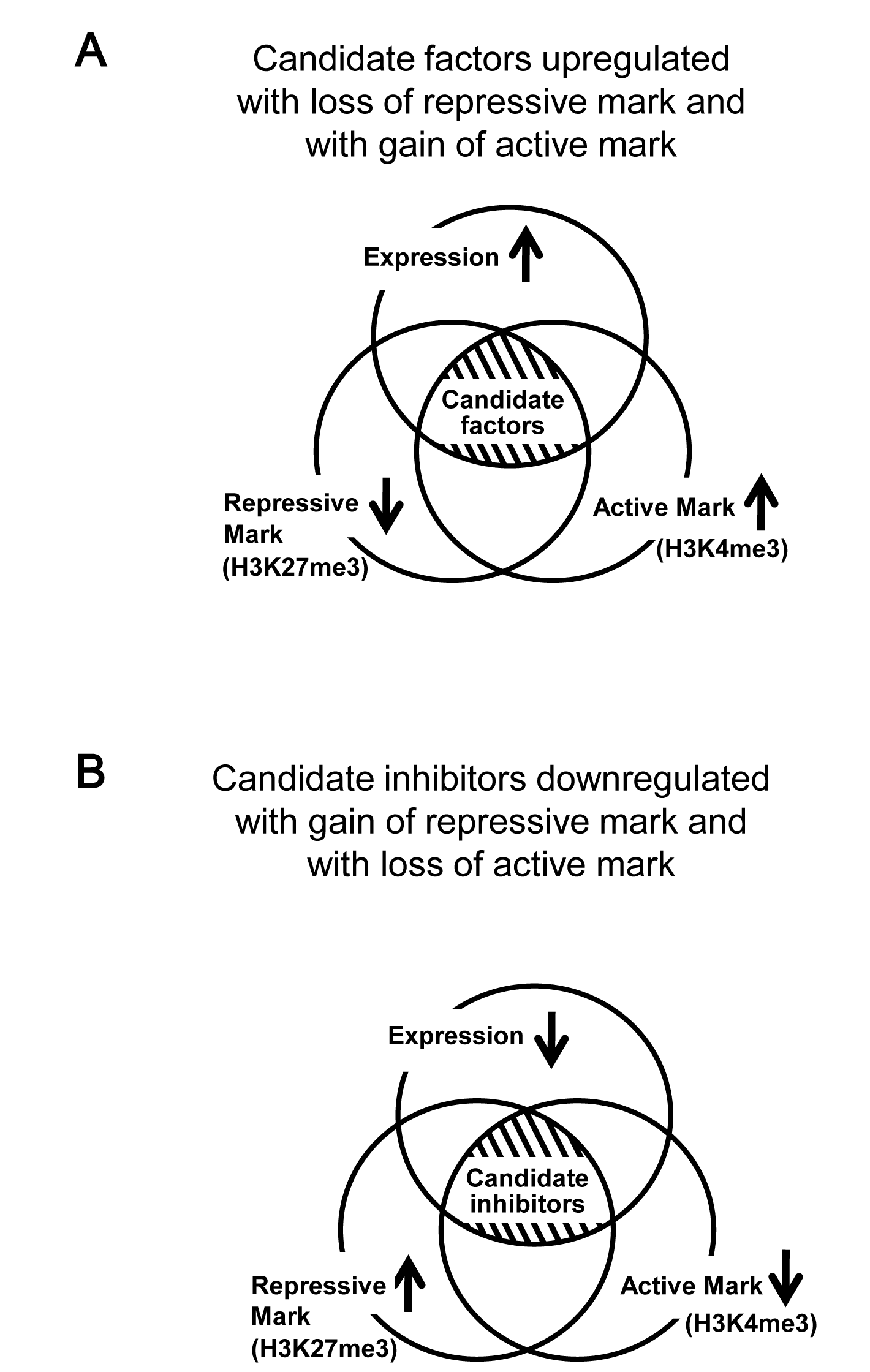

Supplement: Figure S1 — Schema of experiments. (A) Candidate factors to induce senescence are to be identified from genes upregulated, with loss of repressive epigenetic mark and with gain of active mark. (B) Candidate inhibitors to senescence are to be identified from genes downregulated, with gain of repressive epigenetic mark and with loss of active mark. (TIF) [file pgen.1002359.s001.tif]

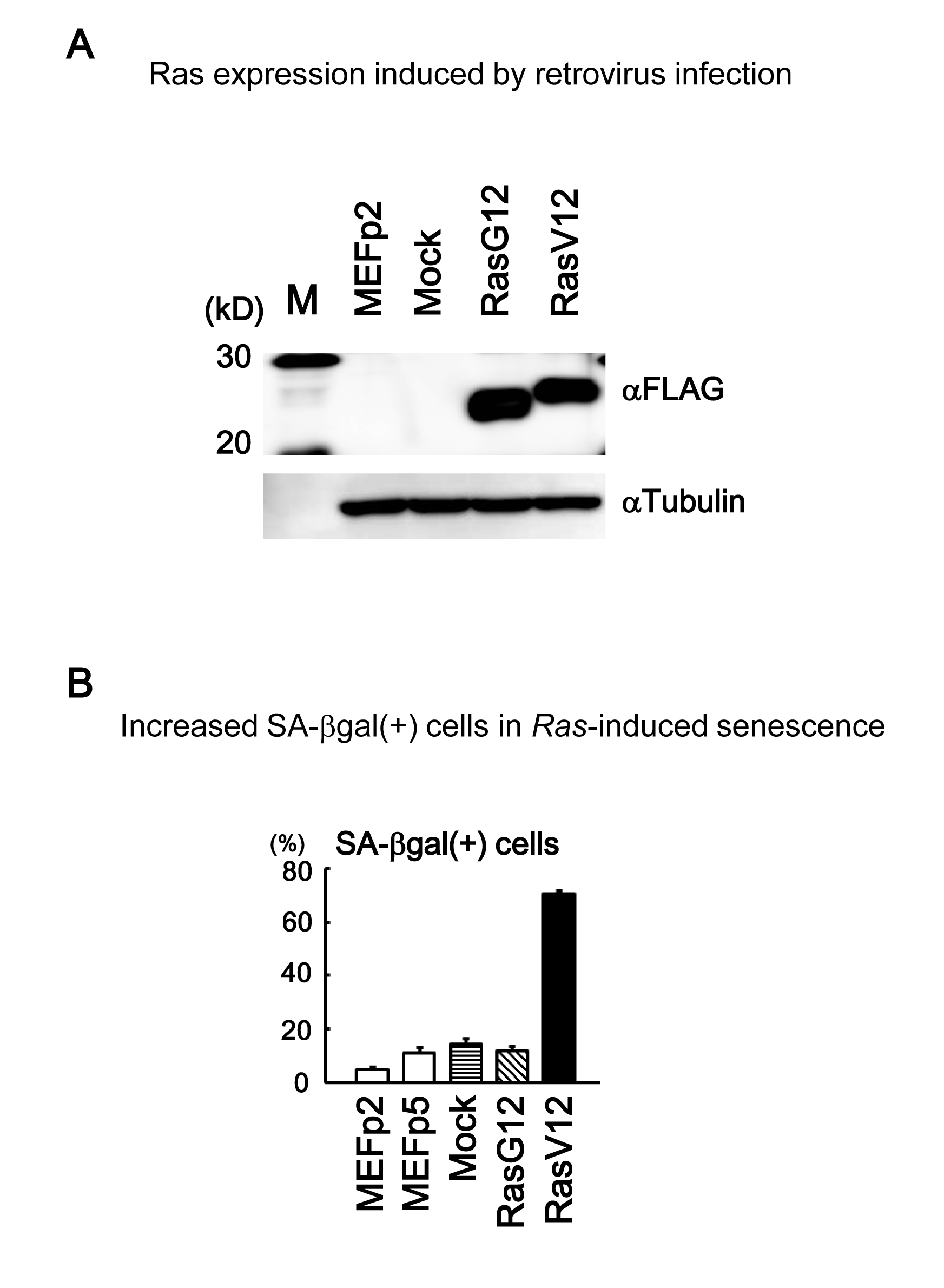

Supplement: Figure S2 — Induction of activated Ras by retrovirus infection. (A) Western blot analysis of Ras protein. Expression of Ras protein with N-terminal FLAG tag was confirmed by western blot analysis using anti-FLAG antibody. (B) Count of senescence-associated β-galactosidase (SA-βgal)-positive cells. MEF cells at passage 2 (MEFp2, open box) rarely showed SA-βgal(+) cells. MEF cells at passage 5 without virus infection (MEFp5, open box) showed slight increase in number of SA-βgal(+) cells, and two control cells infected with mock vector (Mock cells, horizontal-striped box) and wild type Ras (RasG12 cells, crosshatched box) showed similar level of SA-βgal staining, indicating stress-induced senescence during passages. The stress response is a consequence of the high oxygen levels that inflict oxidative damage to the cells resulting in senescence [16]. RasV12-infected cells (RasV12 cells, closed box) showed marked increase in number of SA-βgal(+) cells. Representatives were mean and standard error in three repeated experiments. (TIF) [file pgen.1002359.s002.tif]

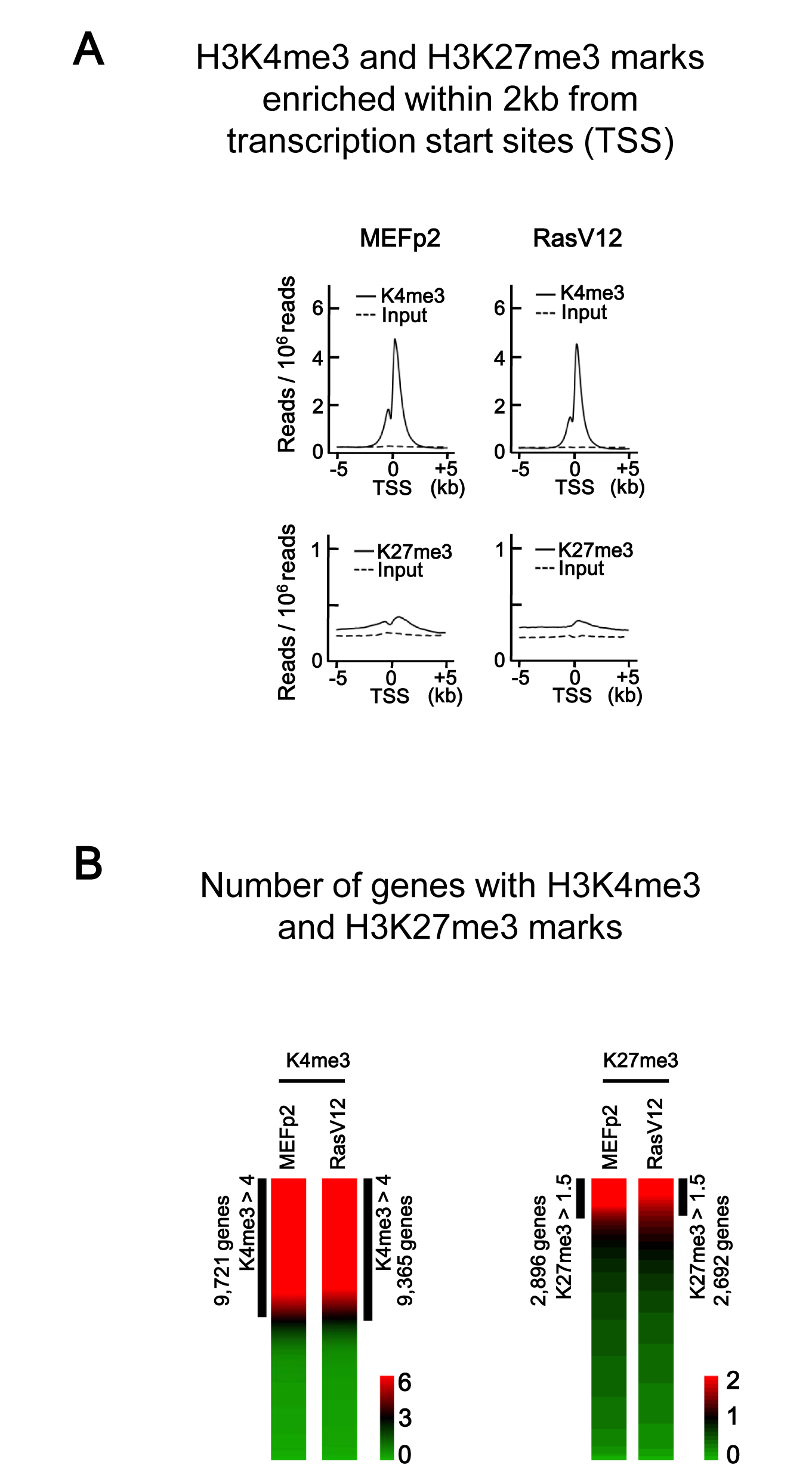

Supplement: Figure S3 — Distribution of epigenetic marks. (A) Distribution of H3K4me3 and H3K27me3 marks around TSS was shown by the number of mapped Solexa reads per million reads within a window size of 300 bp and 500 bp, respectively. The distribution was similar between MEFp2 and RasV12. The peak of H3K4me3 mark was detected at +247 bp for MEFp2, and at +312 bp for RasV12, and distribution was rather narrow. The peak of H3K27me3 mark was detected at +698 bp for MEFp2, and at +420 bp for RasV12, and distribution was rather wide than H3K4me3. (B) Epigenetic statuses of H3K4me3 and H3K27me3 for each gene were decided by the maximum number of mapped reads per million reads in a window size of 300 bp and 500 bp, respectively, within 2 kb ± TSS of each gene, and distribution of the epigenetic status of 20,232 genes was shown by heat map. The number of H3K27me3(+) genes was generally decreased. (TIF) [file pgen.1002359.s003.tif]

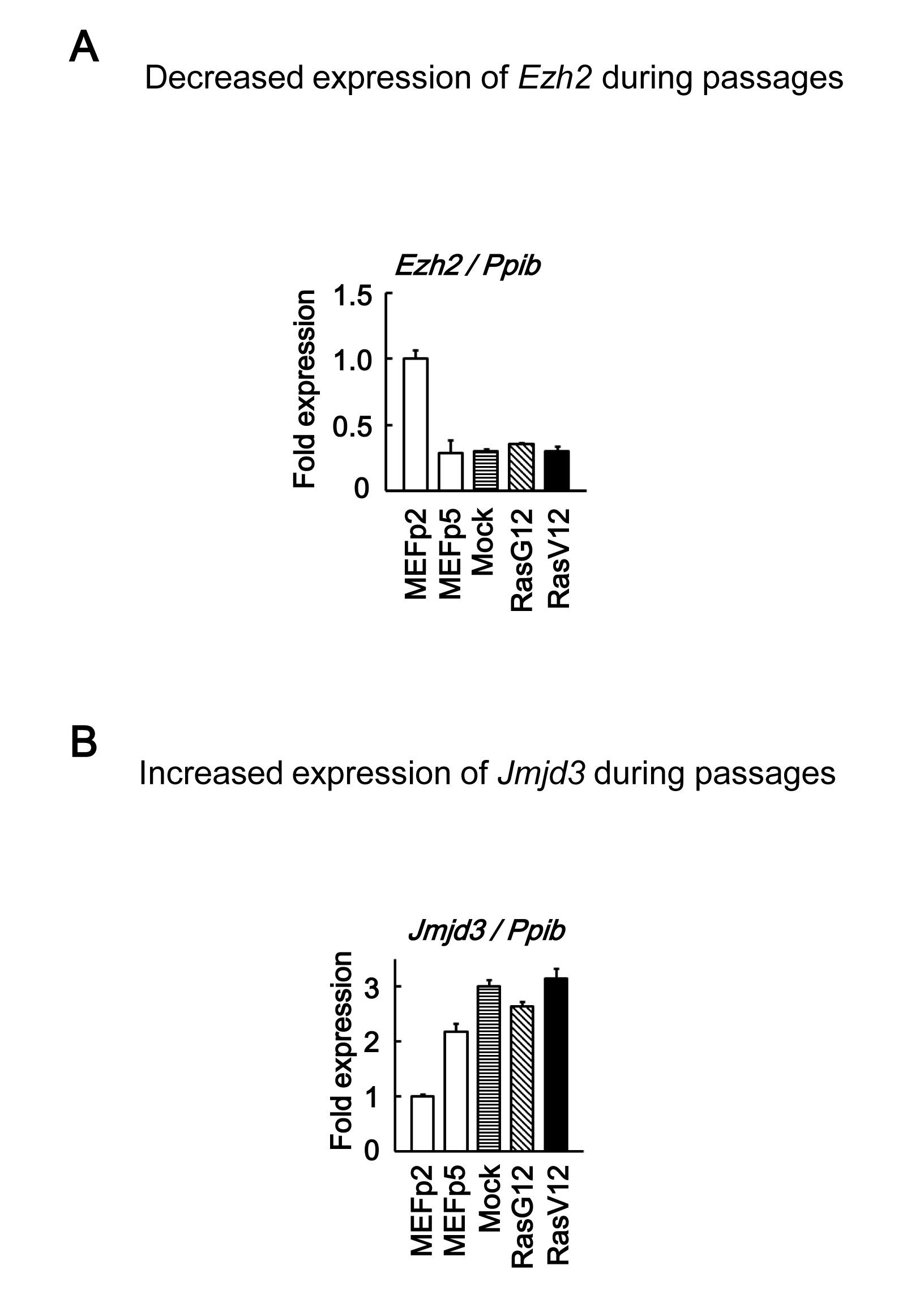

Supplement: Figure S4 — Expression analysis of Ezh2 and Jmjd3. Real-time RT-PCR was performed, and normalized to Ppib, and relative expression levels compared to MEFp2 was shown. Ezh2 expression level was decreased in RasV12-induced senescence, but similar downregulation was observed during three passages without viral infection, or with mock and RasG12 infection. Similarly, Jmjd3 expression level was increased in RasV12-induced senescence, but similar upregulation was observed during three passages without viral infection, or with mock and RasG12 infection. (TIF) [file pgen.1002359.s004.tif]

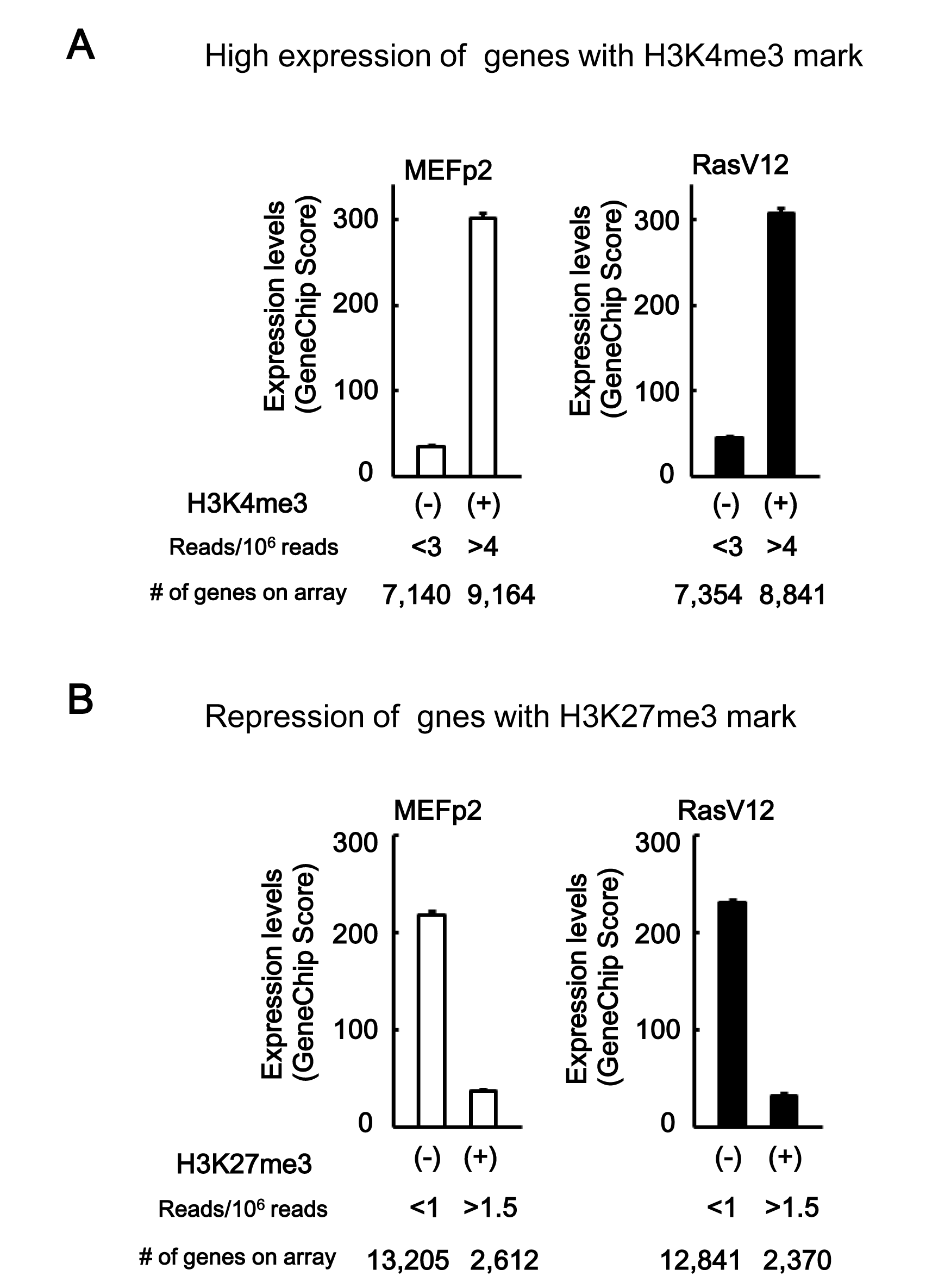

Supplement: Figure S5 — Relation between expression and histone marks (+/−). (A) For epigenetic status of H3K4me3, genes with >4 reads per million reads within a window size of 300 bp were regarded as H3K4me3(+), and genes with <3 reads were as H3K4me3(-). Mean and standard error of expression signal (GeneChip score) were shown. Markedly high expression was confirmed in H3K4me3(+) genes. (B) For epigenetic status of H3K27me3, genes with >1.5 reads per million reads within a window size of 500 bp were regarded as H3K27me3(+), and genes with <1 read were as H2K27me3(-). Markedly low expression was confirmed in H3K27me3(+) genes. (TIF) [file pgen.1002359.s005.tif]

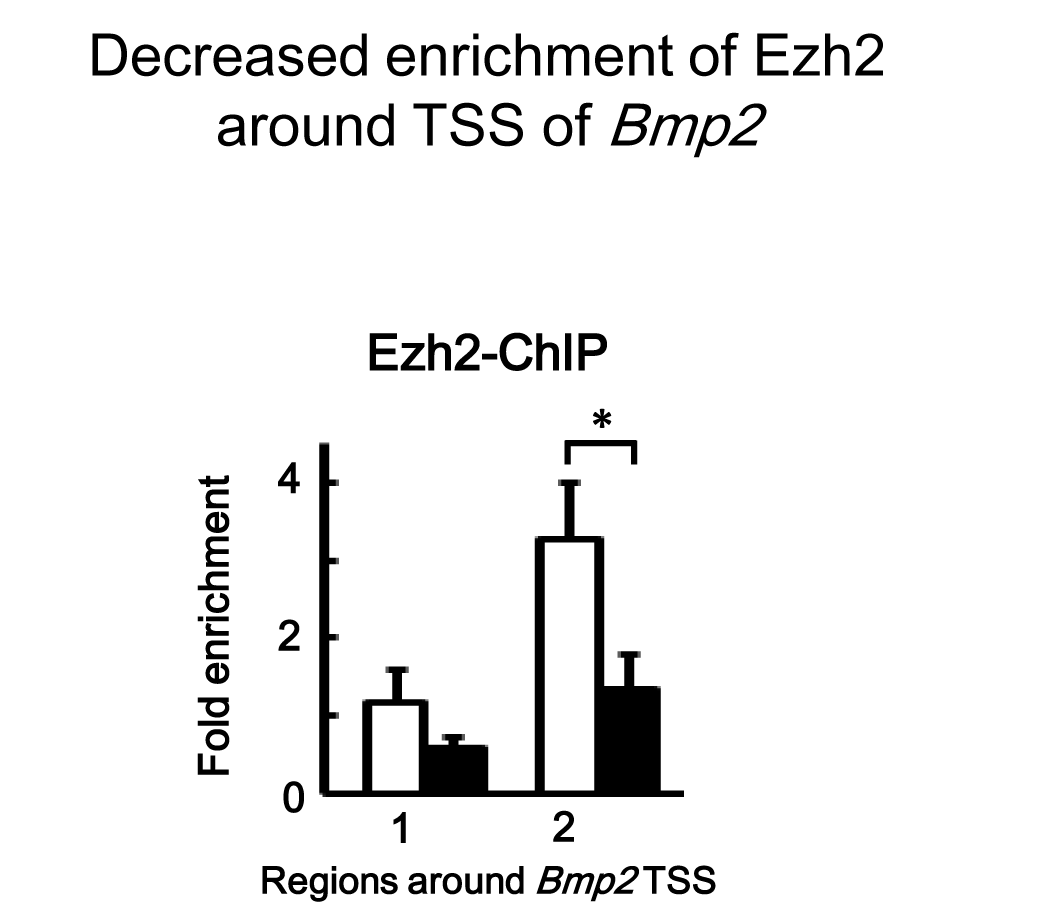

Supplement: Figure S6 — Decreased enrichment of Ezh2 around TSS of Bmp2. Quantitative ChIP-PCR was performed for ∼100 bp upstream (region 1) and ∼300 bp downstream (region 2) of Bmp2 TSS (See Figure 3A), and shown by relative fold enrichment compared to Actb (Figure 1D). Decreased enrichment of Ezh2 around Bmp2 TSS was shown (*P<0.05). (TIF) [file pgen.1002359.s006.tif]

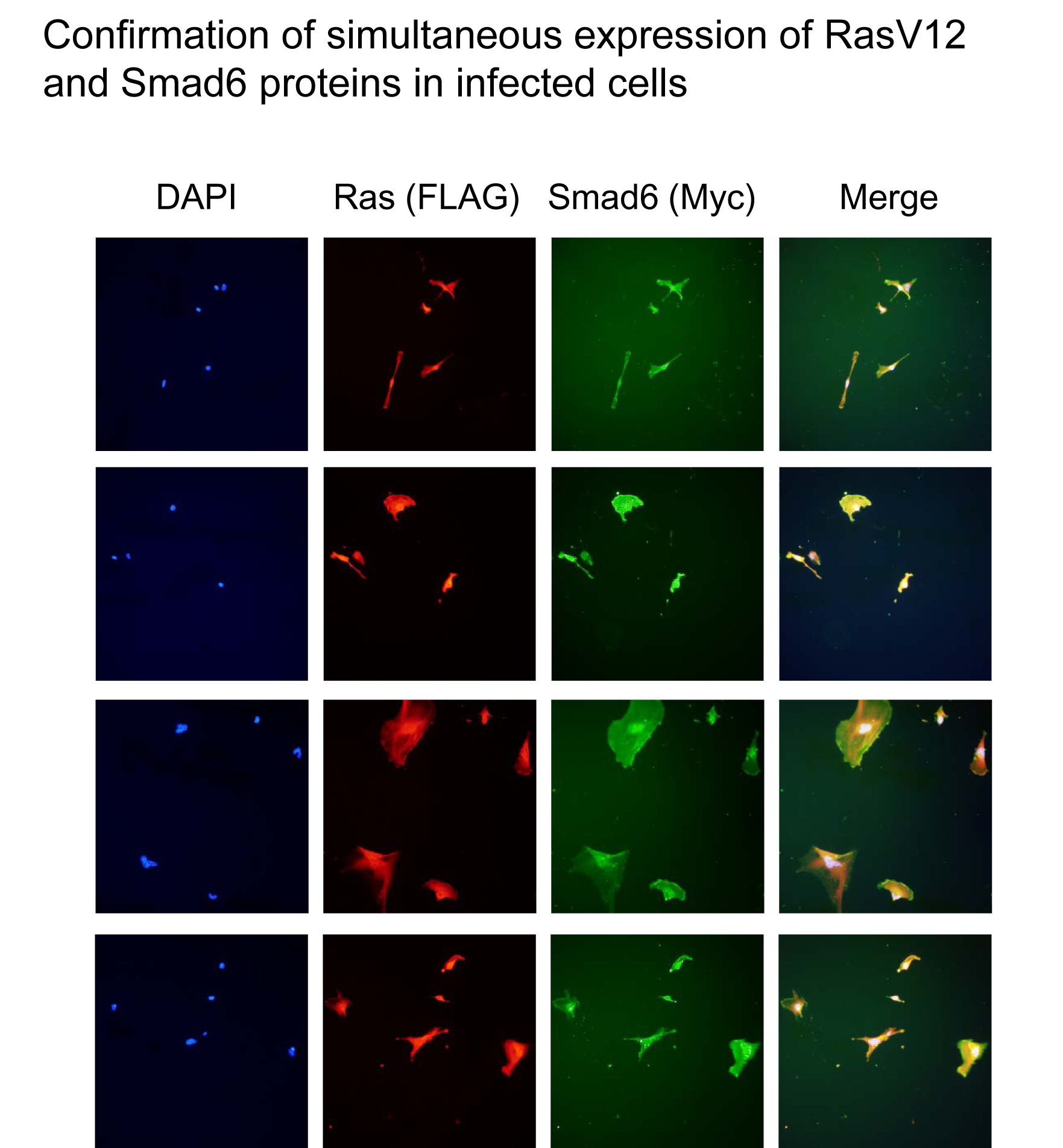

Supplement: Figure S7 — Cellular immunofuorescence for RasV12 and Smad6. RasV12 with N-terminal FLAG and Smad6 with N-terminal Myc were detected using anti-FLAG and anti-Myc antibodies. Simultaneous expression of RasV12 and Smad6 proteins in Smad6-introduced RasV12 cells was confirmed (See also Figure 4E). (TIF) [file pgen.1002359.s007.tif]

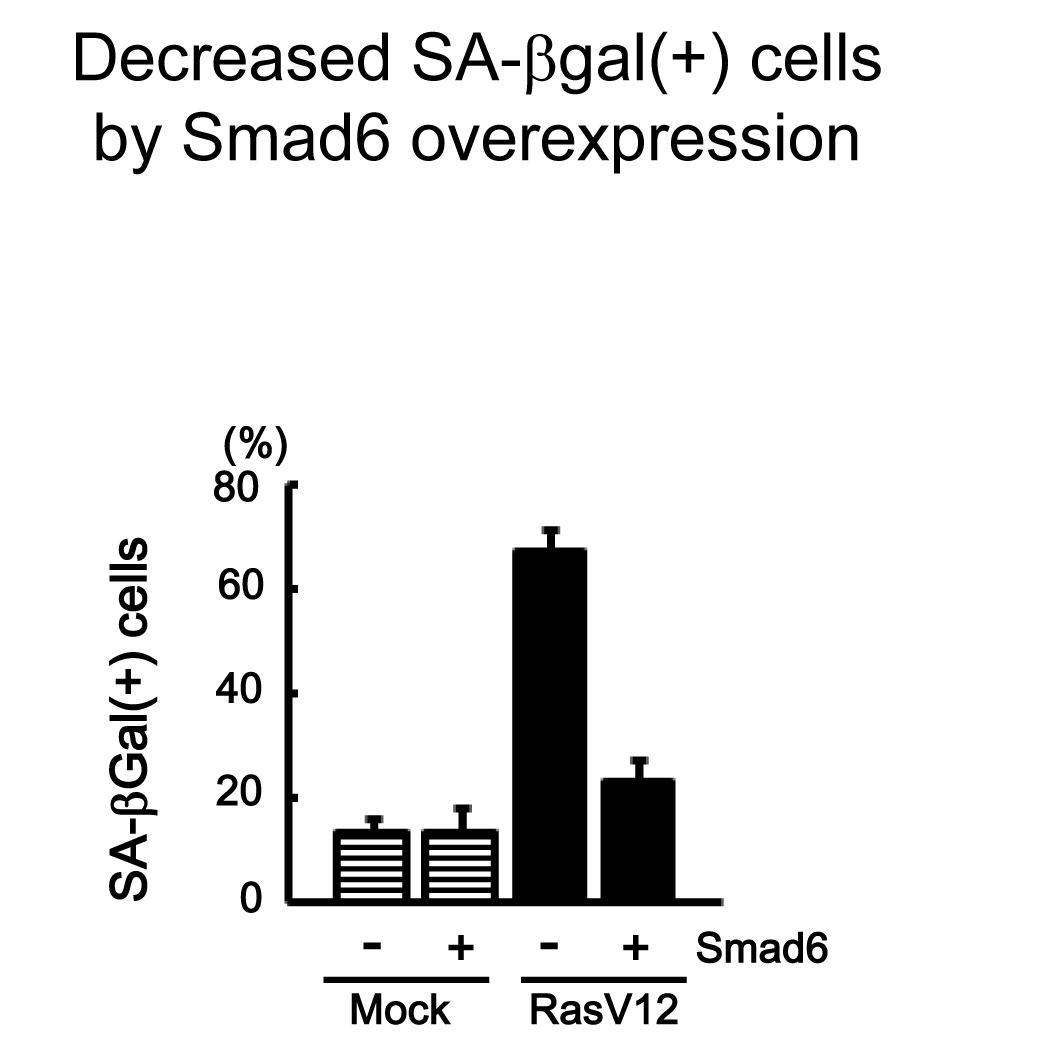

Supplement: Figure S8 — Decreased SA-βgal(+) cells by Smad6 overexpression. Retrovirus of Smad6 cDNA was infected with Mock or RasV12 retrovirus. Smad6-induced RasV12 cells showed decreased number of SA-βgal(+) cells compared to RasV12 cells (black box). Smad6 induction did not affect on Mock cells (horizontally striped box). (TIF) [file pgen.1002359.s008.tif]

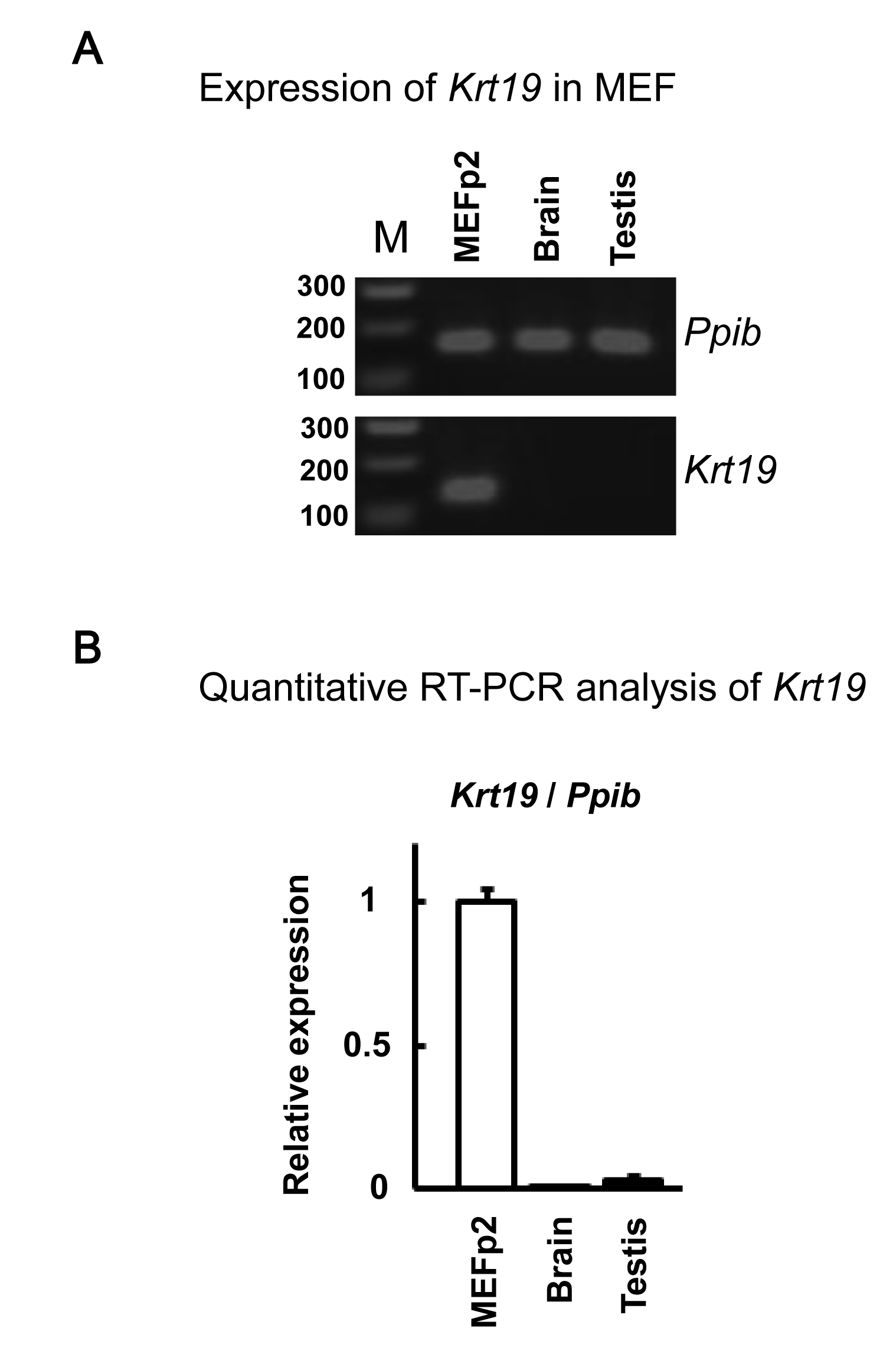

Supplement: Figure S9 — Expression of Krt19 in MEF. (A) RT-PCR showed that MEFp2 showed expression of Krt19 in MEFp2 and not in brain and testis. (B) Real-time RT-PCR showed that Krt19 expression level in MEFp2 was much higher than brain and testis. (TIF) [file pgen.1002359.s009.tif]

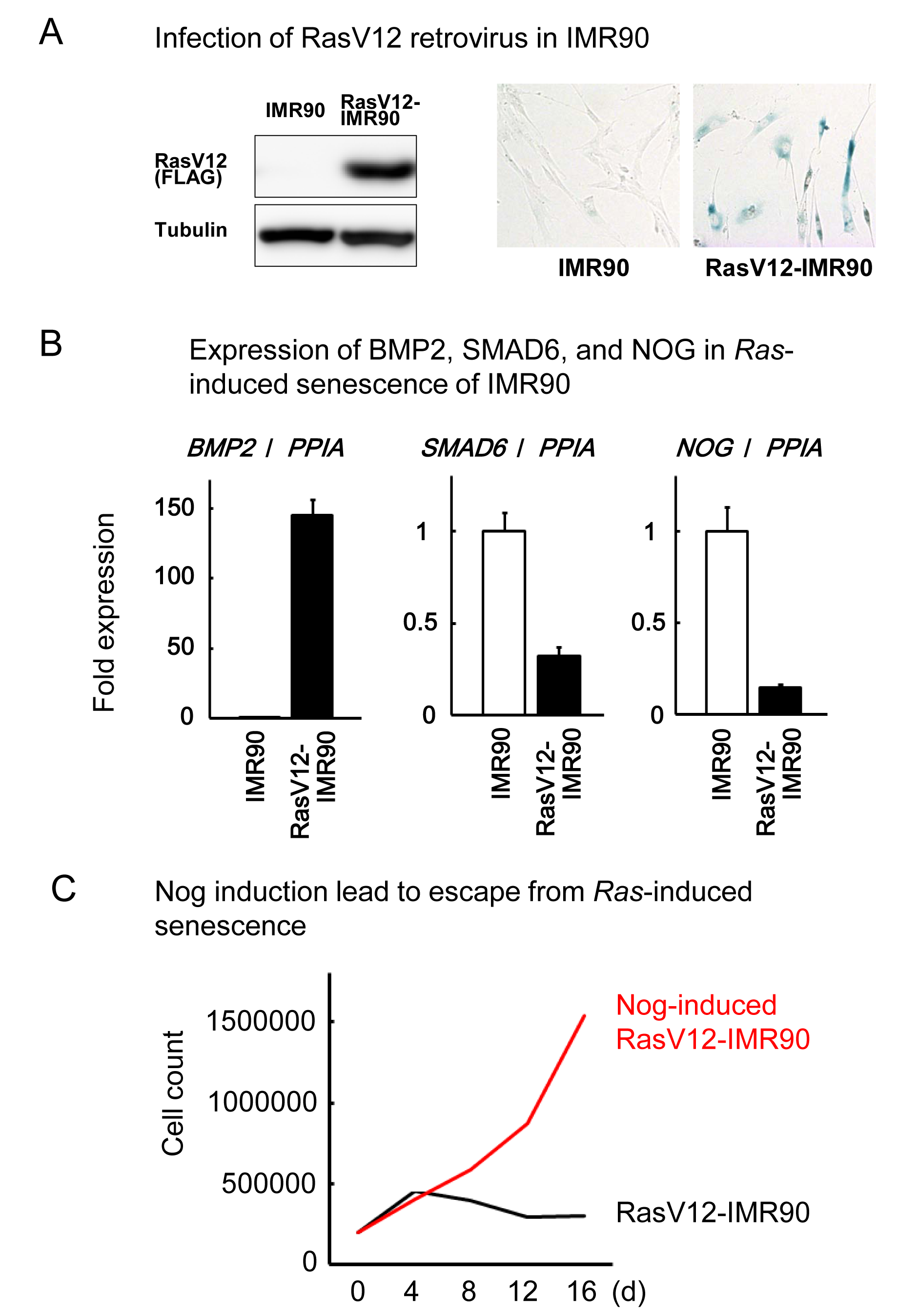

Supplement: Figure S10 — Ras-induced senescence in human fibroblast IMR90. (A) IMR90 was infected with RasV12 retrovirus. In western blotting, expression of Ras protein with N-terminal FLAG tag on day 4 was detected using anti-FLAG antibody (left). SA-βgal staining on day 7 showed that RasV12-IMR90 cells fell into senescence (right). (B) Real-time RT-PCR showed that BMP2 expression was markedly increased to 145-fold in RasV12-IMR90 cells, while SMAD6 and NOG expressions were decreased to 0.32-fold and 0.15-fold, respectively. (C) Nog-induced RasV12-IMR90 cells showed continual cellular growth. (TIF) [file pgen.1002359.s010.tif]

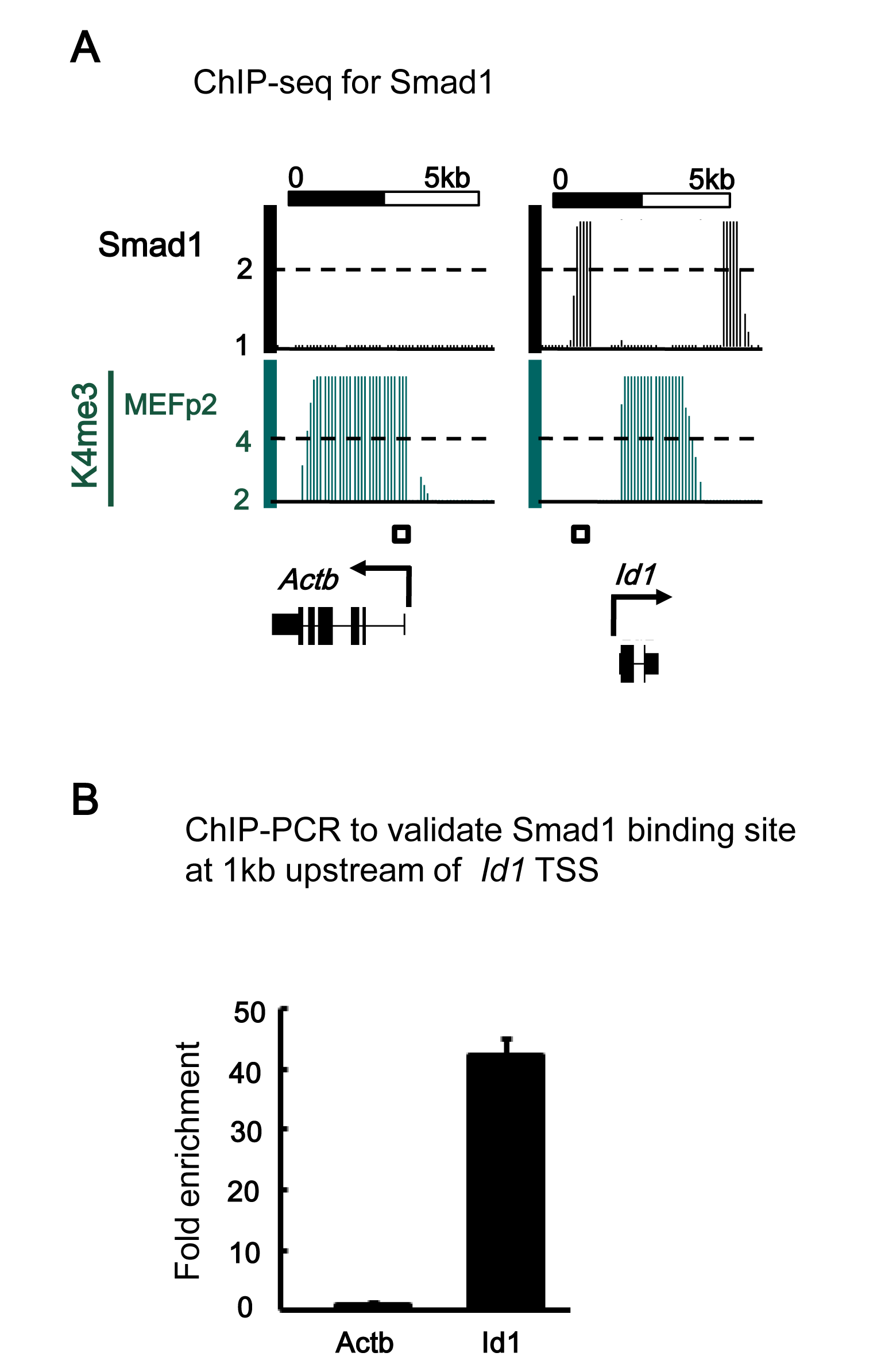

Supplement: Figure S11 — ChIP using anti-Smad1 antibody. (A) There was no Smad1 binding site detected around Actb, while a Smad1 binding site was detected at 1 kb upstream of Id1 TSS. open squares, regions for ChIP-PCR. (B) Smad1 binding at 1 kb upstream of Id1 TSS was validated by ChIP-PCR. (TIF) [file pgen.1002359.s011.tif]

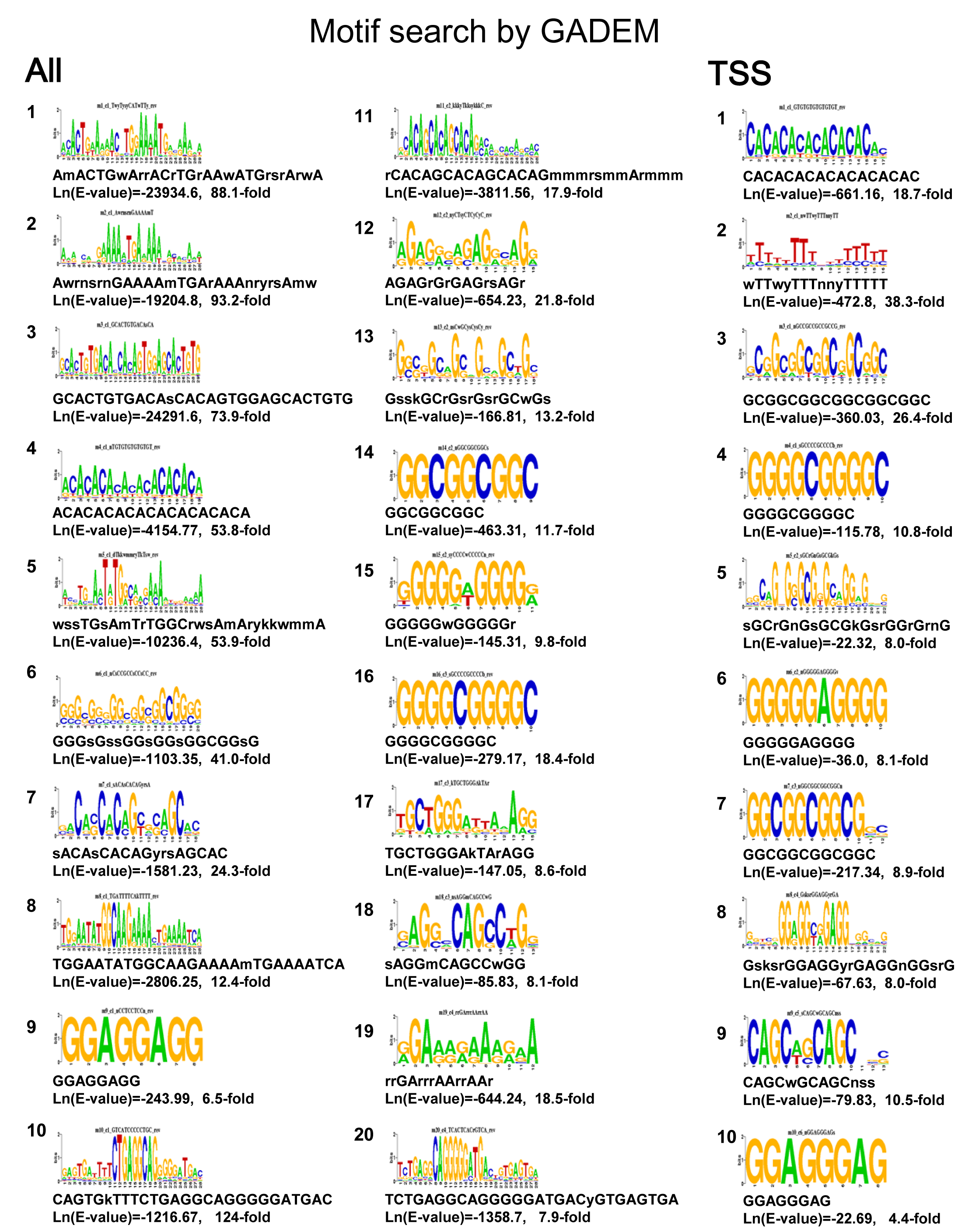

Supplement: Figure S12 — The motifs within Smad1 binding regions by GADEM. GADEM version1.3 (http://www.niehs.nih.gov/research/resources/software/gadem/index.cfm) was used to search for the motifs with default parameters except -posWt (Weight profile for positions on the sequence) = 1, -pv (P-value cutoff) = 0.00001, -em (Number of EM steps) = 20, and -fullScan = 1. The enriched sequences were drawn by STAMP (http://www.benoslab.pitt.edu/stamp/). Ln(E-value) and fold-enrichment to sites in background sequence were shown. 20 and 10 motifs were obtained in whole genomic region (All) and TSS regions (TSS), respectively. GGGGCGGGGC was commonly detected in both analyses. (TIF) [file pgen.1002359.s012.tif]

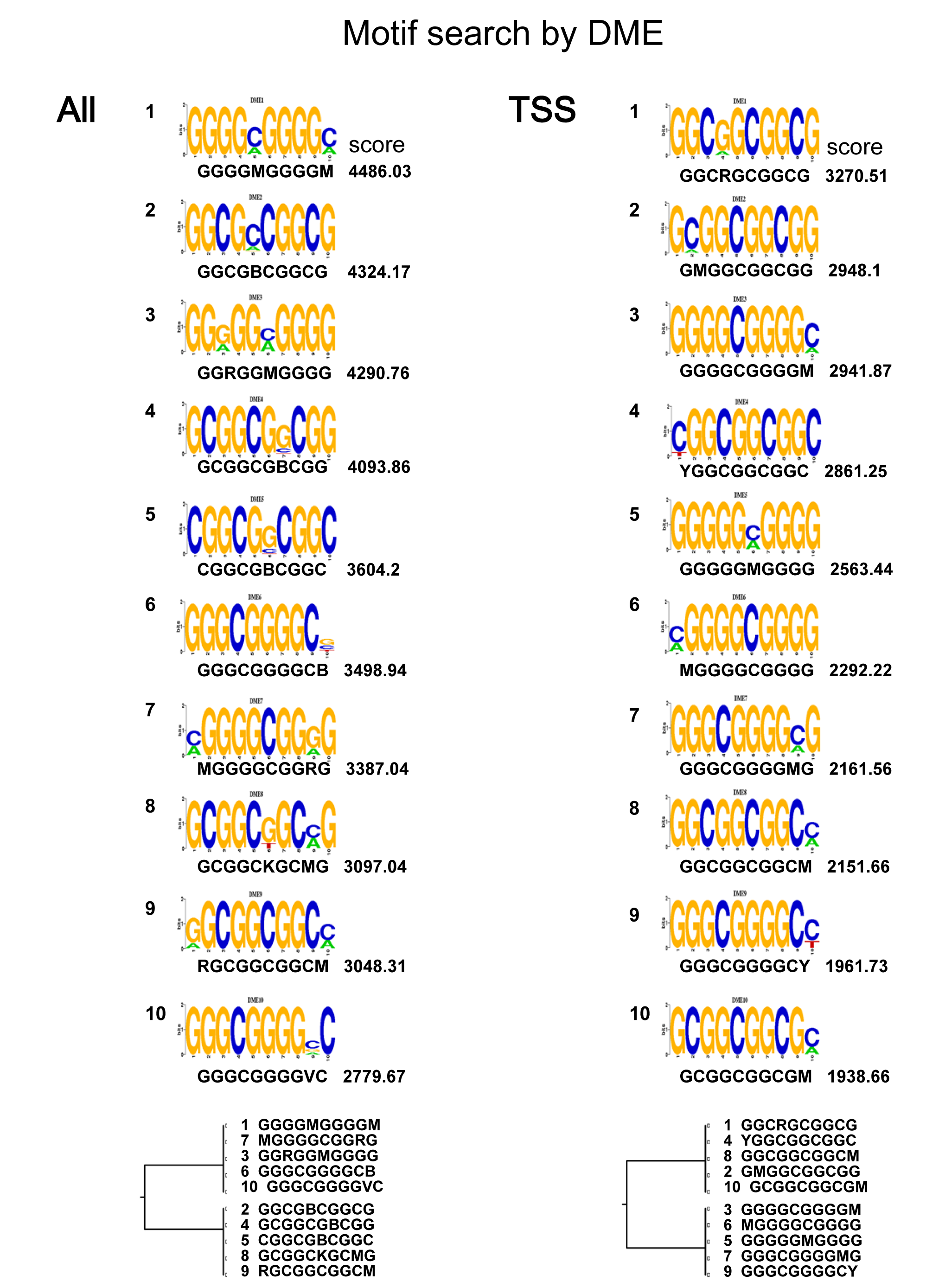

Supplement: Figure S13 — The motifs within Smad1 binding regions by DME. To confirm the GADEM result (Figure S9), the motifs were searched for by another software DME2 (http://rulai.cshl.edu/dme/), using ZOOPS model with default parameters except -w (minimum desired motif width) = 10 and -n (number of motifs to produce) = 10. The results were drawn by STAMP, including the tree view at the bottom. It was confirmed that DNA sequences very similar to GGGGCGGGGC were enriched, such as GGGGCGGGGM and GGGCGGGGC. (TIF) [file pgen.1002359.s013.tif]

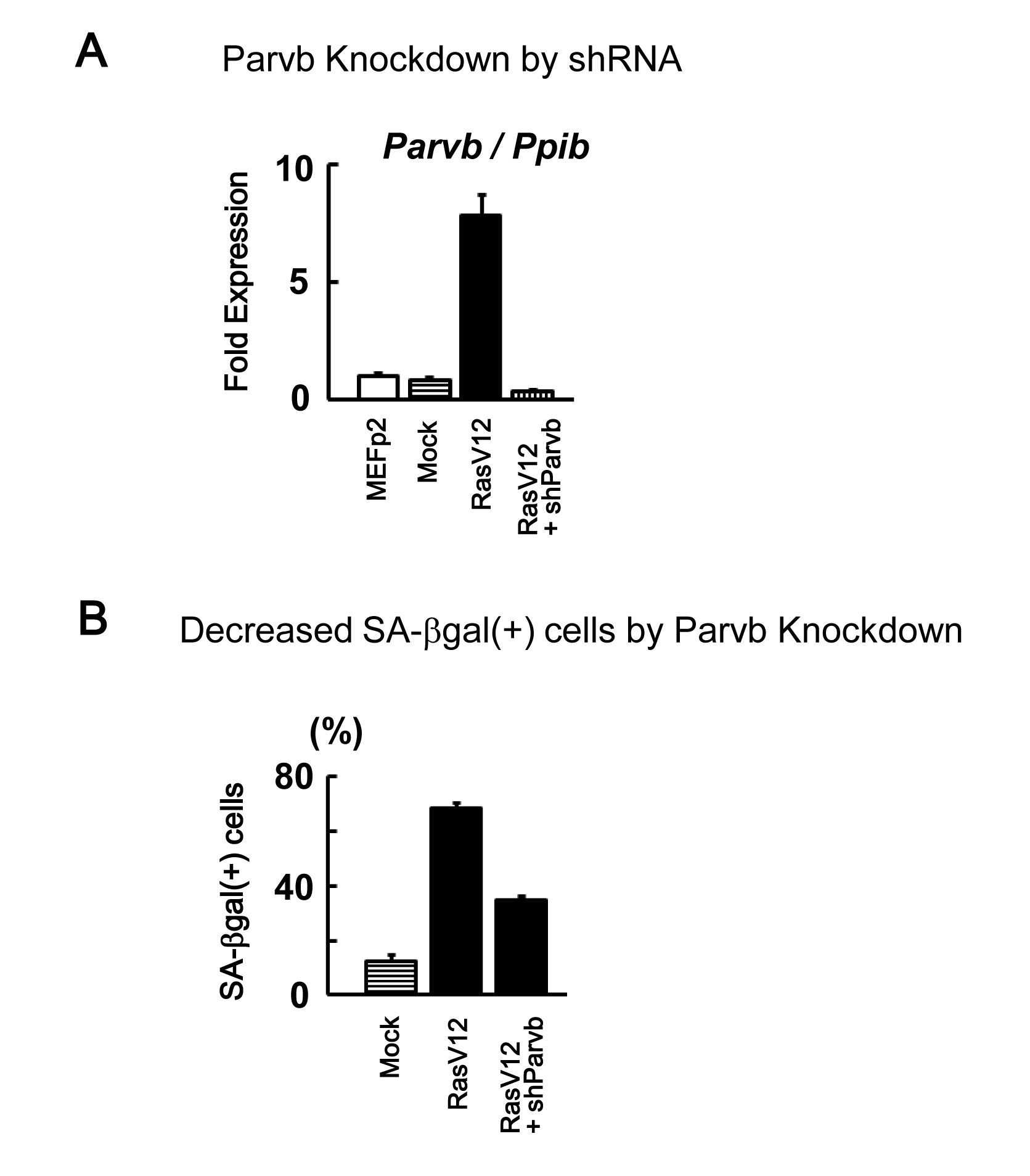

Supplement: Figure S14 — Parvb in Ras-induced senescence. (A) Real-time RT-PCR showed that Parvb was knocked down by shRNA to 0.05-fold. (B) Parvb-knocked down RasV12 cells showed partially decreased number of SA-βgal(+) cells compared to RasV12 cells, though higher than Mock level. (TIF) [file pgen.1002359.s014.tif]
