# Supplementary material for: Activation of Bmp2-Smad1 Signal and Its Regulation by Coordinated Alteration of H3K27 Trimethylation in Ras-Induced Senescence
Source: PLoS Genet. 2011 Nov 3;7(11):e1002359. doi: 10.1371/journal.pgen.1002359 (PMC3207904; doi:10.1371/journal.pgen.1002359)
Supplement: Table S1 — Significant terms with P<10−10 were listed. When there were less than five terms with P<10−10, top five terms with P<10−5 were listed for each category. * The term included Bmp2. (DOC) [file pgen.1002359.s015.doc]

Supporting Table S1. Gene annotation enrichment analysis for 822 genes upregulated in RasV12 cells at day 10 by >5-fold, compared to MEFp2

| *Category* | Term | P-value |
| --- | --- | --- |
| *Gene Ontology_biological process* | | |
|  | cell differentiation* | 3.8X10^-10^ |
|  | cellular developmental process* | 3.8X10^-10^ |
|  | system development* | 1.1X10^-6^ |
|  | developmental process* | 1.1X10^-6^ |
|  | anatomical structure development* | 1.2X10^-6^ |
| *Gene Ontology_cellular compornent* | | |
|  | extracellular region* | 1.2X10^-21^ |
|  | extracellular region part* | 2.4X10^-18^ |
|  | extracellular space* | 6.3X10^-18^ |
| *Gene Ontology_molecular function* | | |
|  | hormone activity | 1.6X10^-7^ |
|  | peptidase activity | 5.3X10^-7^ |
|  | serine-type endopeptidase activity | 6.1X10^-7^ |
|  | receptor binding* | 7.5X10^-7^ |
|  | endopeptidase activity | 8.1X10^-7^ |
| *Swiss Prot PIR database_keywords* | | |
|  | Signal* | 9.9X10^-33^ |
|  | Glycoprotein* | 2.1X10^-26^ |
|  | Secreted* | 1.8X10^-19^ |
| *Uniprot Sequence Feature* | | |
|  | signal peptide* | 4.8X10^-18^ |
|  | glycosylation site:N-linked (GlcNAc...)* | 3.0X10^-14^ |
|  | disulfide bond* | 1.3X10^-12^ |
|  | topological domain:Extracellular | 5.3X10^-8^ |
|  | topological domain:Cytoplasmic | 1.6X10^-7^ |
