# Supplementary material for: Activation of Bmp2-Smad1 Signal and Its Regulation by Coordinated Alteration of H3K27 Trimethylation in Ras-Induced Senescence
Source: PLoS Genet. 2011 Nov 3;7(11):e1002359. doi: 10.1371/journal.pgen.1002359 (PMC3207904; doi:10.1371/journal.pgen.1002359)
Supplement: Table S2 — Significant terms with P<10−10 were listed. When there were less than five terms with P<10−10, top five terms with P<10−5 were listed for each category. § The term included Smad6. (DOC) [file pgen.1002359.s016.doc]

Supporting Table S2. Gene annotation enrichment analysis for 735 genes downregulated in RasV12 cells at day 10 by >5-fold, compared to MEFp2

| *Category* | Term | P-value |
| --- | --- | --- |
| *Gene Ontology_biological process* | | |
|  | M phase | 1.4X10^-22^ |
|  | cell cycle | 7.2X10^-22^ |
|  | cell cycle phase | 2.0X10^-21^ |
|  | cell cycle process | 5.9X10^-21^ |
|  | mitosis | 5.2X10^-20^ |
|  | M phase of mitotic cell cycle | 6.4X10^-20^ |
|  | cell division | 2.2X10^-18^ |
|  | mitotic cell cycle | 3.2X10^-18^ |
| *Gene Ontology_cellular compornent* | | |
|  | chromosome, pericentric region | 7.1X10^-18^ |
|  | extracellular region part | 2.9X10^-14^ |
|  | extracellular region | 9.2X10^-14^ |
|  | extracellular space | 5.6X10^-12^ |
|  | chromosome | 7.2X10^-12^ |
| *Gene Ontology_molecular function* | | |
|  | protein binding^§^ | 1.4X10^-8^ |
|  | binding^§^ | 2.8X10^-6^ |
|  | chemokine activity | 6.4X10^-6^ |
| *Swiss Prot PIR database_keywords* | | |
|  | mitosis | 2.7X10^-21^ |
|  | cell division | 6.2X10^-18^ |
|  | Secreted | 7.9X10^-18^ |
|  | cell cycle | 1.8X10^-17^ |
|  | signal | 4.3X10^-16^ |
|  | centromere | 1.2X10^-13^ |
| *Uniprot Sequence Feature* | | |
|  | signal peptide | 1.0X10^-11^ |
|  | disulfide bond | 2.5X10^-6^ |
