# Supplementary material for: Activation of Bmp2-Smad1 Signal and Its Regulation by Coordinated Alteration of H3K27 Trimethylation in Ras-Induced Senescence
Source: PLoS Genet. 2011 Nov 3;7(11):e1002359. doi: 10.1371/journal.pgen.1002359 (PMC3207904; doi:10.1371/journal.pgen.1002359)
Supplement: Table S3 — H3K4me3 and H3K27me3 levels were shown by the maximum number of the mapped Solexa reads per million reads within a window size of 300 bp and 500 bp, respectively, for 2 kb around TSS. Expression levels were shown by GeneChip score. Secreted factors Bmp2 and Igfbp3 were shown to be highly upregulated with H3K27me3 loss and H3K4me3 gain. (DOC) [file pgen.1002359.s017.doc]

Supporting Table S3. 30 genes with H3K4me3 gain and H3K27me3 loss

| Gene names | NM# | Chr | H3K4me3 | |  | H3K27me3 | |  | Expression (GeneChip score) | | | |
| --- | --- | --- | --- | --- | --- | --- | --- | --- | --- | --- | --- | --- |
|  |  |  | MEFp2 | RasV12 |  | MEFp2 | RasV12 |  | MEFp2 | RasV12 | RasV12 | RasV12 |
|  |  |  |  |  |  |  |  |  |  | Day3 | Day7 | Day10 |
| Nefm | NM_008691 | 14 | 1.2 | 7.9 |  | 4.6 | 0.8 |  | 17.8 | 468.1 | 1219.1 | 1308.2 |
| Sh2d5 | NM_001099631 | 4 | 1.5 | 8.0 |  | 1.6 | 0.5 |  | 5.8 | 365.8 | 300.3 | 221.1 |
| Bmp2 | NM_007553 | 2 | 1.8 | 7.4 |  | 2.1 | 0.5 |  | 26.1 | 716.9 | 1596.1 | 687.2 |
| Igfbp3 | NM_008343 | 11 | 1.6 | 5.7 |  | 2.3 | 1.0 |  | 53.9 | 2370 | 2162.3 | 2429.4 |
| Chd7 | NM_001081417 | 4 | 2.9 | 4.4 |  | 2.0 | 1.0 |  | 0.7 | 22.7 | 24.1 | 16.4 |
| C030014K22Rik | NM_175461 | 1 | 1.8 | 13.4 |  | 2.7 | 0.5 |  | 2.2 | 41.3 | 71.5 | 61.8 |
| Upp1 | NM_009477 | 11 | 2.4 | 7.5 |  | 4.0 | 0.5 |  | 26.2 | 814.5 | 675.3 | 515.3 |
| Foxg1 | NM_008241 | 12 | 1.7 | 5.5 |  | 3.1 | 0.8 |  | 41.1 | 650.5 | 452.1 | 787.2 |
| Kcnh2 | NM_013569 | 5 | 1.1 | 5.5 |  | 2.3 | 0.5 |  | 12.8 | 224.8 | 205.8 | 127.4 |
| p16/Cdkn2a | NM_001040654 | 4 | 0.9 | 5.1 |  | 2.3 | 0.5 |  | 18.1 | 99.2 | 247.4 | 197.7 |
| Syngr1 | NM_009303 | 15 | 2.6 | 9.1 |  | 3.0 | 0.9 |  | 13.4 | 78.2 | 162.5 | 182.1 |
| Abca1 | NM_013454 | 4 | 2.8 | 6.0 |  | 1.5 | 0.9 |  | 78.3 | 299.2 | 761.1 | 737.3 |
| Gpr149 | NM_177346 | 3 | 3.0 | 8.8 |  | 1.5 | 0.6 |  | 30.6 | 165.9 | 184.9 | 276.9 |
| Slc25a23 | NM_025877 | 17 | 2.3 | 7.9 |  | 2.9 | 0.8 |  | 4.2 | 6.7 | 15.1 | 37.7 |
| Kcnh1 | NM_001038607 | 1 | 0.8 | 4.4 |  | 2.3 | 0.8 |  | 10.5 | 61 | 93.7 | 44 |
| Ngef | NM_019867 | 1 | 2.6 | 4.5 |  | 1.8 | 0.9 |  | 22 | 167.6 | 55.8 | 63.8 |
| Gramd1b | NM_172768 | 9 | 2.6 | 7.5 |  | 1.9 | 0.9 |  | 22.2 | 80.3 | 103.5 | 115.5 |
| Igsf11 | NM_170599 | 16 | 2.4 | 5.2 |  | 1.5 | 0.6 |  | 15.4 | 55.4 | 75 | 69 |
| Egr3 | NM_018781 | 14 | 2.7 | 7.0 |  | 2.6 | 0.6 |  | 78.3 | 376.6 | 274.9 | 301.7 |
| OTTMUSG00000015750 | NM_001085521 | 2 | 2.1 | 7.0 |  | 3.1 | 1.0 |  | 46.7 | 173.3 | 180.2 | 153.4 |
| Nav1 | NM_173437 | 1 | 1.8 | 5.0 |  | 1.6 | 0.4 |  | 139.8 | 436.4 | 538 | 476.2 |
| Zfp296 | NM_022409 | 7 | 2.3 | 5.7 |  | 1.9 | 0.6 |  | 11.2 | 23.1 | 33.2 | 28.4 |
| Olfm1 | NM_001038612 | 2 | 3.0 | 4.5 |  | 1.9 | 0.9 |  | 62.6 | 108.3 | 164.2 | 124.5 |
| Sdk1 | NM_177879 | 5 | 2.3 | 4.2 |  | 2.5 | 0.9 |  | 9.8 | 16.7 | 20.4 | 25.6 |
| 2410025L10Rik | NM_028596 | 5 | 1.8 | 4.5 |  | 2.4 | 1.0 |  | 26.2 | 43.5 | 53.2 | 55.3 |
| Hoxa4 | NM_008265 | 6 | 2.6 | 5.1 |  | 2.1 | 1.0 |  | 10.3 | 2.8 | 16 | 8.2 |
| Nr2f1 | NM_010151 | 13 | 3.0 | 5.6 |  | 1.5 | 0.6 |  | 85.5 | 112.1 | 74.6 | 129.8 |
| Lor | NM_008508 | 3 | 2.5 | 4.6 |  | 1.6 | 0.7 |  | 39 | 38.9 | 56.3 | 45.4 |
| Cadm4 | NM_153112 | 7 | 2.3 | 6.0 |  | 3.0 | 0.8 |  | 27.5 | 35 | 36 | 36.4 |
| Cacna1a | NM_007578 | 8 | 1.7 | 5.2 |  | 2.0 | 1.0 |  | 27.8 | 16.2 | 17.7 | 28.5 |
