# Supplementary material for: Activation of Bmp2-Smad1 Signal and Its Regulation by Coordinated Alteration of H3K27 Trimethylation in Ras-Induced Senescence
Source: PLoS Genet. 2011 Nov 3;7(11):e1002359. doi: 10.1371/journal.pgen.1002359 (PMC3207904; doi:10.1371/journal.pgen.1002359)
Supplement: Table S4 — H3K4me3 and H3K27me3 levels were shown by the maximum number of the mapped Solexa reads per million reads within a window size of 300 bp and 500 bp, respectively, for 2 kb around TSS. Expression levels were shown by GeneChip score. Two Bmp2/Smad1 signal inhibitors, Smad6 and Nog, were included in the nine genes with H3K27me3 gain and H3K4me3 loss. (DOC) [file pgen.1002359.s018.doc]

Supporting Table S4. 9 genes with H3K27me3 gain and H3K4me3 loss

| Gene names | NM# | Chr | H3K4me3 | |  | H3K27me3 | |  | Expression (GeneChip score) | | | |
| --- | --- | --- | --- | --- | --- | --- | --- | --- | --- | --- | --- | --- |
|  |  |  | MEFp2 | RasV12 |  | MEFp2 | RasV12 |  | MEFp2 | Mock | RasG12 | RasV12 |
|  |  |  |  |  |  |  |  |  |  | Day3 | Day7 | Day10 |
| Ednra | NM_010332 | 8 | 4.1 | 1.4 |  | 0.9 | 1.7 |  | 40.7 | 15.7 | 25 | 14 |
| E2f2 | NM_177733 | 4 | 5.7 | 1.8 |  | 0.8 | 1.9 |  | 2.5 | 7.7 | 3.2 | 0.8 |
| Palm2 | NM_172868 | 4 | 9.9 | 0.9 |  | 0.4 | 1.9 |  | 56.3 | 17.7 | 12.7 | 21.3 |
| Slit2 | NM_178804 | 5 | 10.1 | 2.4 |  | 0.8 | 1.5 |  | 218.6 | 67.3 | 34.9 | 51 |
| Nog | NM_008711 | 11 | 4.0 | 1.1 |  | 1.0 | 1.5 |  | 12.4 | 1.9 | 5.5 | 2.6 |
| Pde3a | NM_018779 | 6 | 5.6 | 2.4 |  | 1.0 | 1.5 |  | 18.8 | 1.3 | 5.7 | 1.9 |
| Adamts5 | NM_011782 | 16 | 4.1 | 2.8 |  | 1.0 | 3.1 |  | 3.7 | 0.3 | 0.2 | 0.2 |
| Smad6 | NM_008542 | 9 | 9.7 | 2.2 |  | 0.4 | 2.0 |  | 302.5 | 38.5 | 9.3 | 15.8 |
| Atoh8 | NM_153778 | 6 | 8.7 | 1.8 |  | 1.0 | 1.8 |  | 85.2 | 2.1 | 0.7 | 1.6 |
