# Supplementary material for: Activation of Bmp2-Smad1 Signal and Its Regulation by Coordinated Alteration of H3K27 Trimethylation in Ras-Induced Senescence
Source: PLoS Genet. 2011 Nov 3;7(11):e1002359. doi: 10.1371/journal.pgen.1002359 (PMC3207904; doi:10.1371/journal.pgen.1002359)
Supplement: Table S5 — Smad1 target genes were generally upregulated by Bmp2 stimulation in MEF (Figure 6), but genes with H3K27me3 increase e.g. Smad6 and Atoh8 were repressed in RasV12 cells. Smad1 target genes without H3K27me3 increase were correlated to upregulation (Figure 7 and Table S6). (DOC) [file pgen.1002359.s019.doc]

Supporting Table S5. 50 Smad1 target genes with H3K27me3 increase

| Gene names | NM# | Chr | H3K4me3 | |  | H3K27me3 | |  | Expression (GeneChip score) | | | |
| --- | --- | --- | --- | --- | --- | --- | --- | --- | --- | --- | --- | --- |
|  |  |  | MEFp2 | RasV12 |  | MEFp2 | RasV12 |  | MEFp2 | RasV12 | RasV12 | RasV12 |
|  |  |  |  |  |  |  |  |  |  | Day3 | Day7 | Day10 |
| Npnt | NM_033525 | 3 | 4.7 | 4.3 |  | 1.3 | 1.7 |  | 184 | 467.6 | 250.4 | 188.2 |
| 1600016N20Rik | NM_028050 | 7 | 5.7 | 8.0 |  | 0.7 | 1.5 |  | 11.7 | 10.7 | 16.7 | 20.5 |
| Mpv17l | NM_033564 | 16 | 3.1 | 2.3 |  | 1.2 | 1.9 |  | 24.9 | 24.7 | 41.2 | 36.5 |
| Tmem181 | NM_001033178 | 17 | 0.9 | 1.1 |  | 1.3 | 1.9 |  | 120.6 | 130 | 188.5 | 160 |
| Bbs2 | NM_026116 | 8 | 5.4 | 4.5 |  | 0.6 | 1.1 |  | 14.8 | 15.1 | 21.3 | 15.1 |
| Tmem2 | NM_001033759 | 19 | 10.7 | 10.8 |  | 0.3 | 0.8 |  | 214.5 | 260.7 | 304.8 | 262.2 |
| Grin2d | NM_008172 | 7 | 4.3 | 5.7 |  | 0.8 | 1.3 |  | 10 | 1.5 | 8.4 | 14.1 |
| Zfp768 | NM_146202 | 7 | 10.6 | 8.9 |  | 0.4 | 1.0 |  | 32.4 | 45.5 | 31.3 | 31.5 |
| 1110007C09Rik | NM_026738 | 13 | 5.5 | 10.0 |  | 0.4 | 1.1 |  | 245.6 | 317.3 | 247 | 245.5 |
| Agpat2 | NM_026212 | 2 | 6.1 | 8.1 |  | 0.7 | 1.2 |  | 177.4 | 200.6 | 169.2 | 227.7 |
| Baiap2 | NM_001037755 | 11 | 8.3 | 9.8 |  | 0.9 | 1.8 |  | 102.2 | 130.9 | 97.3 | 104.2 |
| Baiap2 | NM_001037754 | 11 | 8.1 | 9.6 |  | 1.0 | 1.7 |  | 102.2 | 130.9 | 97.3 | 104.2 |
| Sfi1 | NM_030207 | 11 | 10.3 | 14.1 |  | 6.3 | 11.0 |  | 16.6 | 21.1 | 18.4 | 19.2 |
| Eef2 | NM_007907 | 10 | 20.8 | 18.5 |  | 1.1 | 2.1 |  | 3489.9 | 4110.3 | 4277.1 | 4246.5 |
| Tbca | NM_009321 | 13 | 9.4 | 16.2 |  | 0.9 | 1.3 |  | 1125.8 | 1107.1 | 1278.4 | 1376.5 |
| Hsp90aa1 | NM_010480 | 12 | 12.0 | 15.4 |  | 0.4 | 1.1 |  | 2515.9 | 2575.3 | 2931.9 | 2340.5 |
| Ppfia3 | NM_029741 | 7 | 3.1 | 1.7 |  | 1.4 | 2.0 |  | 11.3 | 7.1 | 1 | 13.1 |
| Lfng | NM_008494 | 5 | 7.7 | 2.1 |  | 0.7 | 1.2 |  | 15 | 17.3 | 15.1 | 12.5 |
| BC067047 | NM_177782 | 2 | 3.0 | 7.0 |  | 1.4 | 2.3 |  | 13.3 | 11.7 | 15.2 | 13.9 |
| B230208H17Rik | NM_001024616 | 2 | 10.7 | 12.8 |  | 0.5 | 1.0 |  | 147.3 | 165.3 | 104 | 119.1 |
| Pacs2 | NM_001081170 | 12 | 12.0 | 11.1 |  | 0.3 | 0.9 |  | 129.5 | 103.2 | 102 | 141.1 |
| Freq | NM_019681 | 2 | 9.7 | 10.0 |  | 0.7 | 1.1 |  | 214.8 | 230.9 | 180.9 | 203.5 |
| Ddr1 | NM_172962 | 17 | 1.2 | 0.5 |  | 0.6 | 1.0 |  | 94.3 | 47.6 | 81.4 | 98.7 |
| EG245263 | NM_001034869 | 10 | 0.6 | 0.5 |  | 0.4 | 1.3 |  | 2.6 | 4.9 | 5.4 | 6.3 |
| Shroom1 | NM_027917 | 11 | 5.4 | 6.0 |  | 0.9 | 1.5 |  | 3.7 | 1.2 | 4.6 | 7.9 |
| Trpm8 | NM_134252 | 1 | 0.9 | 1.0 |  | 1.6 | 3.9 |  | 0.7 | 5.2 | 1.1 | 1 |
| C920005C14Rik | NM_177391 | 15 | 1.6 | 1.3 |  | 0.7 | 1.6 |  | 6.8 | 4.1 | 2.4 | 4.5 |
| Adamts5 | NM_011782 | 16 | 4.1 | 2.8 |  | 1.0 | 3.1 |  | 3.7 | 0.3 | 0.2 | 0.2 |
| X99384 | NM_013753 | 10 | 3.7 | 5.8 |  | 1.3 | 1.7 |  | 39.1 | 37.5 | 26.3 | 35.8 |
| Fzd1 | NM_021457 | 5 | 8.5 | 6.6 |  | 0.8 | 1.7 |  | 319.4 | 121.5 | 231.9 | 267.4 |
| Syngr2 | NM_009304 | 11 | 10.5 | 10.2 |  | 0.5 | 1.0 |  | 457.7 | 351 | 284.7 | 372 |
| Hip1 | NM_146001 | 5 | 11.8 | 7.2 |  | 0.3 | 1.0 |  | 123.7 | 74.7 | 97.9 | 83.5 |
| Me3 | NM_181407 | 7 | 5.9 | 3.1 |  | 0.7 | 1.2 |  | 13.1 | 7.4 | 4.7 | 4.8 |
| H1fx | NM_198622 | 6 | 9.6 | 3.0 |  | 0.4 | 0.9 |  | 71.8 | 50.4 | 35.7 | 35.7 |
| Gcat | NM_013847 | 15 | 3.3 | 2.9 |  | 0.4 | 0.9 |  | 85.9 | 55 | 23.4 | 28.7 |
| Zfp568 | NM_001033355 | 7 | 8.5 | 8.0 |  | 0.2 | 0.6 |  | 197.6 | 94.8 | 76 | 85.5 |
| Palmd | NM_023245 | 3 | 0.6 | 0.4 |  | 0.8 | 1.2 |  | 22 | 7.9 | 0.8 | 5 |
| Dok5 | NM_029761 | 2 | 7.1 | 3.7 |  | 0.3 | 1.0 |  | 23.4 | 3 | 9.8 | 5.7 |
| Lin9 | NM_175186 | 1 | 8.6 | 8.7 |  | 0.6 | 1.0 |  | 63.5 | 24.1 | 13.9 | 12.4 |
| Palm2 | NM_172868 | 4 | 9.9 | 0.9 |  | 0.4 | 1.9 |  | 56.3 | 17.7 | 12.7 | 21.3 |
| Grin3a | NM_001033351 | 4 | 2.7 | 0.7 |  | 3.2 | 3.6 |  | 28.4 | 1.7 | 0.7 | 4.3 |
| Mfap4 | NM_029568 | 11 | 0.6 | 0.6 |  | 0.6 | 1.5 |  | 29.3 | 0.7 | 1.1 | 0.7 |
| Slc27a3 | NM_011988 | 3 | 4.0 | 1.2 |  | 0.6 | 1.0 |  | 45.4 | 12.3 | 6.1 | 4.5 |
| Tmem119 | NM_146162 | 5 | 3.0 | 1.3 |  | 1.0 | 1.5 |  | 126.7 | 29.6 | 25.8 | 25.3 |
| Runx1t1 | NM_001111026 | 4 | 8.0 | 3.5 |  | 0.6 | 1.0 |  | 140.6 | 29.1 | 26.1 | 29 |
| Adam12 | NM_007400 | 7 | 7.2 | 2.0 |  | 0.7 | 1.1 |  | 372.7 | 53.7 | 39.8 | 27.3 |
| Smad6 | NM_008542 | 9 | 9.7 | 2.2 |  | 0.4 | 2.0 |  | 302.5 | 38.5 | 9.3 | 15.8 |
| Atoh8 | NM_153778 | 6 | 8.7 | 1.8 |  | 1.0 | 1.8 |  | 85.2 | 2.1 | 0.7 | 1.6 |
| Lox | NM_010728 | 18 | 16.5 | 2.1 |  | 0.7 | 1.1 |  | 3954.1 | 159.9 | 188.9 | 304.5 |
| C1qtnf3 | NM_030888 | 15 | 0.7 | 0.3 |  | 0.8 | 1.3 |  | 343.7 | 5 | 4.9 | 1.5 |
