# Supplementary material for: Activation of Bmp2-Smad1 Signal and Its Regulation by Coordinated Alteration of H3K27 Trimethylation in Ras-Induced Senescence
Source: PLoS Genet. 2011 Nov 3;7(11):e1002359. doi: 10.1371/journal.pgen.1002359 (PMC3207904; doi:10.1371/journal.pgen.1002359)
Supplement: Table S6 — Smad1 target genes without H3K27me3 increase were correlated to upregulation (Figure 7). Among Smad1 target genes upregulated in RasV12 cells, top ranking 30 genes showing >3-fold upregulation were listed. None of these genes showed H3K27me3 increase >0.4. These genes were regarded as selectively upregulated Smad1 target genes, and expected to include genes with growth suppressor function. Such genes may perhaps be frequently inactivated in human cancer e.g. by promoter methylation. Among these genes, Parvb was chosen to be examined as Parvb expression was highly induced by BMP2 stimulation in MEF (Figure 6) and our previous methylated DNA immunopreccipitation (MeDIP)-chip analysis of human cancer cell lines [22], [23] showed PARVB promoter methylation in HCT116 and DLD1 (the most right columns above). (DOC) [file pgen.1002359.s020.doc]

Supporting Table S6. Upregulated Smad1 target genes and its promoter methylation in human cancer cells

| Gene names | NM# | Chr | H3K4me3 | |  | H3K27me3 | |  | Expression (GeneChip score) | | | |  | DNA methylation [2,3] | | |
| --- | --- | --- | --- | --- | --- | --- | --- | --- | --- | --- | --- | --- | --- | --- | --- | --- |
|  |  |  | MEF | Ras |  | MEF | Ras |  | MEF | RasV12 | RasV12 | RasV12 |  | HCT | DLD | SW |
|  |  |  | p2 | V12 |  | p2 | V12 |  | p2 | Day3 | Day7 | Day10 |  | 116 | 1 | 480 |
| Sh3bgrl2 | NM_172507 | 9 | 8.8 | 17.3 |  | 0.8 | 0.2 |  | 108 | 1203.8 | 872.1 | 999.1 |  | - | - | - |
| Ugt1a1 | NM_201645 | 1 | 1.4 | 1.3 |  | 2.0 | 1.9 |  | 38 | 343.4 | 423 | 368.1 |  | - | - | - |
| Sema6d | NM_172537 | 2 | 5.3 | 9.0 |  | 1.0 | 1.0 |  | 72.4 | 716.9 | 647.4 | 419.8 |  | - | - | - |
| Atp8a1 | NM_001038999 | 5 | 4.1 | 8.5 |  | 1.0 | 0.8 |  | 5.4 | 28.6 | 94.2 | 54 |  | - | - | - |
| Osbpl6 | NM_145525 | 2 | 7.4 | 13.7 |  | 1.6 | 1.0 |  | 19.9 | 150.8 | 115.8 | 108.8 |  | - | - | - |
| BC031353 | NM_001113283 | 9 | 6.0 | 7.9 |  | 0.5 | 0.2 |  | 37.7 | 120.9 | 276.8 | 145.3 |  | + | + | + |
| A830059I20Rik | NM_021427 | 7 | 3.2 | 7.2 |  | 1.4 | 0.8 |  | 18 | 120.3 | 78.1 | 107.7 |  | NA | NA | NA |
| Bcl2l11 | NM_009754 | 2 | 9.3 | 15.2 |  | 1.4 | 0.5 |  | 70.4 | 177.7 | 405.3 | 274.3 |  | + | + | + |
| Syt7 | NM_018801 | 19 | 1.8 | 3.5 |  | 2.3 | 1.8 |  | 6.8 | 34.8 | 52.7 | 45.8 |  | - | - | - |
| Grasp | NM_019518 | 15 | 11.0 | 12.8 |  | 0.6 | 0.5 |  | 230.4 | 557 | 1162 | 575.8 |  | + | + | - |
| Parvb | NM_133167 | 15 | 8.6 | 16.8 |  | 1.0 | 0.6 |  | 82 | 412.5 | 361 | 356.7 |  | + | + | - |
| Ccdc3 | NM_028804 | 2 | 1.2 | 3.5 |  | 4.1 | 2.0 |  | 29 | 47.3 | 109.1 | 137.8 |  | - | - | - |
| Pcdh7 | NM_018764 | 5 | 10.3 | 13.1 |  | 0.3 | 0.4 |  | 102.9 | 366.8 | 483.6 | 346.7 |  | + | + | + |
| Mef2a | NM_001033713 | 7 | 9.9 | 11.9 |  | 0.9 | 0.4 |  | 33.6 | 75.1 | 146.9 | 79.7 |  | - | - | - |
| Ermp1 | NM_001081213 | 19 | 9.5 | 12.4 |  | 0.6 | 0.8 |  | 64.5 | 201.4 | 271.1 | 189.8 |  | + | - | - |
| Ankrd9 | NM_175207 | 12 | 4.9 | 6.1 |  | 0.8 | 1.0 |  | 8.3 | 42 | 32.8 | 22.7 |  | - | - | - |
| Odz4 | NM_011858 | 7 | 1.7 | 2.6 |  | 2.7 | 1.1 |  | 20.2 | 26.4 | 52.6 | 84.5 |  | NA | NA | NA |
| Cdk2ap2 | NM_026373 | 19 | 5.9 | 4.5 |  | 0.3 | 0.4 |  | 108.8 | 219.3 | 425.1 | 388.6 |  | + | - | + |
| Adcy8 | NM_009623 | 15 | 1.7 | 2.8 |  | 4.8 | 1.9 |  | 1.6 | 37.9 | 27.4 | 13.6 |  | + | + | + |
| Crebl2 | NM_177687 | 6 | 4.8 | 5.8 |  | 0.9 | 0.6 |  | 13.1 | 15.1 | 49.6 | 45.1 |  | - | - | - |
| C230081A13Rik | NM_172924 | 9 | 15.0 | 15.8 |  | 0.3 | 0.3 |  | 125.8 | 355.2 | 411.5 | 458.1 |  | - | - | - |
| Dusp5 | NM_001085390 | 19 | 12.0 | 13.1 |  | 0.5 | 0.4 |  | 247.6 | 832.2 | 791.8 | 728.9 |  | + | + | + |
| 1300014I06Rik | NM_025831 | 13 | 14.0 | 14.0 |  | 0.5 | 0.3 |  | 321 | 677 | 1076.7 | 839.7 |  | - | - | - |
| Dagla | NM_198114 | 19 | 6.2 | 14.5 |  | 1.1 | 1.3 |  | 34.5 | 115.7 | 110.5 | 81.5 |  | - | - | - |
| Dusp6 | NM_026268 | 10 | 19.2 | 20.7 |  | 0.3 | 0.3 |  | 529.5 | 1566.6 | 1747.3 | 1645 |  | - | - | - |
| Hmg20a | NM_025812 | 9 | 15.0 | 15.9 |  | 0.3 | 0.3 |  | 119.7 | 230.3 | 394.6 | 307 |  | - | - | - |
| Tmem65 | NM_175212 | 15 | 15.1 | 17.1 |  | 0.5 | 0.3 |  | 345 | 791.1 | 1099.6 | 833.1 |  | - | - | - |
| Mmd | NM_026178 | 11 | 7.6 | 9.8 |  | 0.8 | 0.7 |  | 653.3 | 1710.7 | 2079.3 | 1513.5 |  | - | - | - |
| Kif21a | NM_001109040 | 15 | 5.9 | 11.3 |  | 0.9 | 0.5 |  | 30 | 44.2 | 93.5 | 67.6 |  | + | - | - |
| Wdr22 | NM_177267 | 12 | 10.6 | 9.7 |  | 0.3 | 0.3 |  | 25.1 | 38 | 58.6 | 75.7 |  | - | - | - |
