# Supplementary material for: Activation of Bmp2-Smad1 Signal and Its Regulation by Coordinated Alteration of H3K27 Trimethylation in Ras-Induced Senescence
Source: PLoS Genet. 2011 Nov 3;7(11):e1002359. doi: 10.1371/journal.pgen.1002359 (PMC3207904; doi:10.1371/journal.pgen.1002359)
Supplement: Table S7 — Ppib and PPIA were used for normalization. (DOC) [file pgen.1002359.s021.doc]

Supporting Table S7. RT-PCR Primers

| Genes | Primer sequences | Products | Anneal |
| --- | --- | --- | --- |
| Mouse |  |  |  |
| *p16*^Ink4a^ | GAGAGCCATCTGGAGCAGCAT and GAAAGAGTTCGGGGCGTTG | 127 bp | 58°C |
| *Bmp2* | TATCATGCCTTTTACTGCCA and ATTCACAGAGTTCACCAGAGTC | 102 bp | 58°C |
| *Smad6* | ATCCCCAAGCCAGACAGTCC and TCCTTGAGCCTCTTGAGCAGC | 133 bp | 63°C |
| *Nog* | TGAGGAGGAAGTTACAGATGTGGC and CAGACTTGGATGGCTTACACAC | 171 bp | 58°C |
| *Parvb* | ACGCCCTGAAGATGTGGTGAA and ATCCTTGTACTTGGTGAACAGC | 79 bp | 58°C |
| *Ezh2* | CATTTCATACGCTCTTCTGTCG and TGTGTTGGGTGTTGCATGGA | 77 bp | 58°C |
| *Jmjd3* | CAGCTCTGGAACTTTCATGCC and CCATAGTTCCGTTTGTGCTCA | 107 bp | 61°C |
| *Krt19* | TTGAGACAGAACACGCCTTGCGT and CTCCTCCTTCAGGCTCTCAATC | 125 bp | 58°C |
| *Dnmt1* | AGACGAGGATGAGAGGGAGGAG and GGGCACTTTGGTGAGTTGATCTT | 144 bp | 58°C |
| *Ppib* | ATGTGGTACGGAAGGTGGAGA and AGCTGCTTAGAGGGATGAGG | 155 bp | 58°C |
| Human |  |  |  |
| *BMP2* | CCTTTGTACGTGGACTTCAGTG and GCATGATTAGTGGAGTTCAGATG | 131 bp | 58°C |
| *SMAD6* | GTCCGATTCCACATTGTCTTAC and GACATGCTGGCGTCTGAGAA | 87 bp | 58°C |
| *NOG* | GCCAGCACTATCTCCACATCCG and GTCTCGTTCAGATCCTTTTCCTT | 112 bp | 58°C |
| *PPIA* | ACAGTGCTTGCTGGCAGTTAGA and CAAATCCGCCACCTCTAGGATAG | 130 bp | 58°C |
