# Supplementary material for: Activation of Bmp2-Smad1 Signal and Its Regulation by Coordinated Alteration of H3K27 Trimethylation in Ras-Induced Senescence
Source: PLoS Genet. 2011 Nov 3;7(11):e1002359. doi: 10.1371/journal.pgen.1002359 (PMC3207904; doi:10.1371/journal.pgen.1002359)
Supplement: Table S8 — Location of TSS was regarded as +1. (DOC) [file pgen.1002359.s022.doc]

Supporting Table S8. ChIP-PCR and MeDIP-PCR primers

| Regions | Primer sequences | Location | Anneal |
| --- | --- | --- | --- |
| *Actb* | TGAGGTACTAGCCACGAGAGAG and ACACCCGCCACCAGGTAAGCA | +60 ~ +128 | 55°C |
| *Dkk1* | AGAGCCATCATTGTAAACACGG and ACCTTTGCCTGTTTGCGTCCT | +91 ~ +179 | 55°C |
| *Gcgr* | TGCTGTCATGTCTGGTGAGTG and GGAGCTGTCAGCACTTGTGTA | +132 ~ +214 | 55°C |
| *p16*_1 | TCCGATCCTTTAGCGCTGTT and CCCGGACTACAGAAGAGATG | -252 ~ -184 | 55°C |
| *p16* _2 | AGGGGTGTTCAATTCATGCTAT and ACACTCTGCTCCTGACCTGG | -130 ~ -50 | 58°C |
| *p16*_3 | GGAGCCACCCATTAAACTAACT and CAAAAATAAGACACTGAAAACTCG | +341 ~ +411 | 55°C |
| *Bmp2*_1 | CTTGGCTGGAGACTTCTTGAACT and TGGAGGCGGCAAGACTGGAT | -155 ~ -59 | 58°C |
| *Bmp2*_2 | ACTGGTGGAGTGGAGTGGAC and CTGGGGTTTGGAATGCCTAA | +235 ~ +321 | 61°C |
| *Smad6*_1 | CGCTTTGTGCTCGTGTACCA and CGATGCTAGAGACACCCTGC | -111 ~ -40 | 61°C |
| *Smad6*_2 | GTGAAACGGGATAGTAAGCCAT and CTAAAAGCTATGTACCGACTGAGG | +1389 ~ +1478 | 58°C |
| *Smad6*_3 | GCTGTCAGTAGGGAAATCACGC and GGCTAAAACTACAGAAAGGGACAA | +1543 ~ +1622 | 55°C |
| *Id1* | CGGGTTTTATGAATGGGTGAC and GCGTCTGAACAAGCGGCTC | -1100 ~ -1035 | 55°C |
| *Nnat* | GAATTTGTAGGCTTGGGTGTGTC and TGGAGGGAAGTCGTGGCTCT | -300 ~ -218 | 55°C |
