# Supplementary material for: Activation of Bmp2-Smad1 Signal and Its Regulation by Coordinated Alteration of H3K27 Trimethylation in Ras-Induced Senescence
Source: PLoS Genet. 2011 Nov 3;7(11):e1002359. doi: 10.1371/journal.pgen.1002359 (PMC3207904; doi:10.1371/journal.pgen.1002359)
Supplement: Table S9 — Location of TSS was regarded as +1. (DOC) [file pgen.1002359.s023.doc]

Supporting Table S9. Bisulfite sequencing primers

| Regions | Primer sequences | Location | Anneal |
| --- | --- | --- | --- |
| *Smad6*_a | GTTGGTGGTTTTTTTAAGGTTTTAAAG and AACCCCAACCCCACATAAAACTCT | +704 ~ +946 | 58°C |
| *Smad6*_b | GATTGTTGTTTTTGGAGTAGGAGTTTAA and TCAACTCCACTACCTACTACAAATCTAACC | +1529 ~ +1762 | 58°C |
| *Bmp2* | GAGGTTGTGTGTTAGTATTTGGT and CCTAAAACAACAACTAAACTTCTAT | -172 ~ +66 | 60°C |
| *Adcy4* | TGGTTATATTAGAGGGATTAAGAGTGA and TACTTCCCATACTCAAATTACATTCT | -420 ~ -130 | 58°C |
| *Sdpr* | TGAGGGAGAATTAGTATAATATGGAATAG and ATTCTCCAATCTCTTCACCTAAAC | +475 ~ +692 | 58°C |
| *Sfrp1* | GAG GTT TCG GGA AGT TTG TAG and ATA ACA CAA CCT CAA ATC CAC C | +196 ~ +474 | 60°C |
| *Glt25d2* | GTAGTTTGGATTAGGGGAATTT and CACCGACACTAATTTCTAAAACTC | -35 ~ +141 | 57°C |
| *Itga11* | GGGTTTAGATTGGTTTGGAGTT and AAACCCCTAAAAAAATCCATAAC | -115 ~ +104 | 60°C |
| *Shisa2* | GGTGAAGGAAAGTGAAGGGTT and CTCCCCGACTCCTACTACAAC | -116 ~ +182 | 60°C |
| *Gypc* | GTTGAAGTGTGTTTATTTTGGAG and TCTAACAAAAATTAAAACATCCC | +34 ~ +282 | 57°C |
