# Supplementary material for: Activation of Bmp2-Smad1 Signal and Its Regulation by Coordinated Alteration of H3K27 Trimethylation in Ras-Induced Senescence
Source: PLoS Genet. 2011 Nov 3;7(11):e1002359. doi: 10.1371/journal.pgen.1002359 (PMC3207904; doi:10.1371/journal.pgen.1002359)
Supplement: Text S1 — Supporting Materials and Methods, and references for them, are available in Text S1. (DOC) [file pgen.1002359.s024.doc]

**Supporting Text S1**

**Supporting Materials and Methods**

**Cells and infection.** Mouse embryonic fibroblast (MEF) was established from 13.5 embryonic day embryos of C57/B6. Embryos were minced with head and red organs taken away, and digested with trypsin/EDTA for 15 minutes at 37°C twice. The cells were seeded onto 10-cm cell culture dishes and cultured in Dulbecco’s modified Eagle’s medium (DMEM) containing 100 units/ml penicillin and 100 g/ml streptomycin sulfate, supplemented with 10% (vol/vol) fetal bovine serum at 37 °C in 5% CO_2_. After cells were passed twice (MEFp2), cells were infected with retroviruses for 48 hours. Then cells were seeded at density of 1×10^5^ cells / 6-cm dish (day 0), and were exposed to 4 μg/mL puromycin during days 0-3. Cells were passed on days 3, 7, and 10. Human fibroblast IMR90 (JCRB9054) was purchased from Health Science Research Resources Bank (Osaka, Japan), and cultured in modified Eagle’s medium (MEM) containing 100 units/ml penicillin and 100 g/ml streptomycin sulfate, supplemented with 10% (vol/vol) fetal bovine serum and non-essential amino acid at 37 °C in 5% CO_2_. After retrovirus infection, 2 μg/mL puromycin were used for selection, and cells were passed on days 4, 8, and 12. Total RNA was collected from MEFp2, from virus-infected MEFs at days 3, 7, and 10, and virus-infected IMR90 at day 4 using TRIzol (Invitrogen, Carlsbad, CA). This study was certified by Animal Ethics Committee in Tokyo University.

**MEF of Nog-trangenic (Nog-Tg) mice.** Nog-Tg mice using keratin 19 gene promoter, mouse Nog cDNA and SV40 p(A) cassette, were previously established [1], and were crossed with wild type C57/B6 mice five times to obtain C57/B6 background. Nog-Tg female mouse was crossed with C57B6, and Tg(-) and Tg(+) MEFs were established from 13.5 embryonic day embryos of the same mother, as above. Each embryo was minced separately, and Tg(-) and Tg(+) MEFs were pooled after genotyping each MEF, and used for experiments. For genotyping PCR, primers, annealing temperature and PCR length are: GTACGCGTGGAATGACCTAGG (Nog-Forward) and GCAAAGGGTCGCTACAGACGT (IRES-Reverse), 55 °C, and 495 bp.

**Retroviral vectors.** To induce senescence in MEF, we constructed retroviral vectors for Ras by cloning cDNAs for wild type HRAS (RasG12) and mutated HRAS (RasV12) by reverse-transcription PCR products from HMEC and SK-BR3 cell RNA, respectively, with N-terminal FLAG tag into a CMV promoter driven expression vector pMX that contains puromycin resistance gene (a kind gift of T. Kitamura, Division of Cellular Therapy, The Institute of Medical Science, The University of Tokyo, Tokyo, Japan) and check the sequences. HMEC was obtained from Cambrex, and SK-BR-3 was obtained from American Tissue Culture Collection. Mock pMX vector (mock), and vectors containing RasG12 and activated RasV12 were transfected into plat-E packaging cells (a kind gift of T. Kitamura) using FuGENE 6 Transfection Reagent (Roche, Mannheim, Germany) to prepare retroviruses. *Smad6* cDNA with N-termainal 6x Myc tag (K. Miyazono), *Nog* cDNA (M. Oshima) with C-terminal V5 tag, and *Parvb* cDNA with C-terminal V5 tag were also cloned into pMX vector, and retroviruses were prepared using plat-E cells as above. Retroviruses of RasV12 and Nog for human fibroblast were prepared using Retrovirus Packaging Kit Ampho (#6161, TaKaRa Bio Inc, Shiga, Japan).

**shRNA retrovirus.** To knock down *Bmp2* or *Parvb*, double strand oligonucleotide DNA to express small hairpin RNA (shRNA) against *Bmp2* or *Parvb* was cloned into RNAi-Ready pSIREN-RetroQ Vector (Clontech, CA). Oligonucleotide sequences are:

GATCCG GGACACCAGGTTAGTGAAT TTCAAGAGA ATTCACTAACCTGGTGTCC CTTTTTTCTCGAGG for *Bmp2*, and

GATCCG TTCTACCTGACTCCTGACA TTCAAGAGA TGTCAGGAGTCAGGTAGAA CTTTTTTCTCGAGG for *Parvb*,

with the 19-mer target sense and antisense sequences underlined. Viral packaging for shRNA retrovirus vectors against *Bmp2* and *Parvb* (shBmp2 and shParvb, respectively) were also done using plat-E cells and FuGENE 6. To MEFp2, RasV12 and shRNA vectors were simultaneously infected for 48h. To expose *Bmp2*-knocked-down RasV12 cells to recombinant BMP protein (rBMP2, R&D systems #355-BM), shBmp2 and RasV12 were infected to MEFp2 for 48h, then cells were cultured in medium with 10% serum and 0, 20 or 200 ng/mL rBMP from day 0.

**Expression array analysis.** For genome-wide transcription analysis, GeneChip Mouse Genome 430 2.0 Array (Affymetrix) was used. Data were collected and analyzed by an Affymetrix GeneChip Scanner 3000 (Affymetrix). The GeneChip data were analyzed using the Affymetrix GeneChip Operating Software v1.3 by MAS5 algorithms, to obtain signal value (GeneChip score) for each probe. For global normalization, the average signal in an array was made equal to 100. Expression array data is available at GEO datasets (#GSE18125).

**Gene annotation enrichment analysis.** Gene annotation enrichment analysis was done for Gene Ontology (biological process, cellular component, and molecular function) and for Functional Categories (Swiss Prot PIR database keywords, and Uniprot Sequence Feature) using the Functional Annotation tool at DAVID Bioinformatics Resources (http://david.abcc.ncifcrf.gov/).

**Chromatin immunoprecipitation (ChIP).** MEF cells without viral infection and those with mock, RasG12 or RasV12 infection at day 10 were cross-linked with 1% formaldehyde for 10 min at room temperature and were prepared for ChIP. ChIP using anti-H3K4me3 (ab8580, abcam, rabbit polyclonal), H3K27me3 (07-142, Upstate, rabbit polyclonal), or Ezh2 (#39103, Active Motif, rabbit polyclonal) antibody was performed. Briefly, cells were crosslinked with 1% formalin for 10 min, and crosslinked cell lysates underwent fragmentation by sonication and incubated with antibodies bound to protein A-sepharose beads (50% slurry) overnight at 4 ºC. The beads were washed several times and eluted with elution buffer (1%SDS, 0.1 M NaHCO3). The eluates were treated with 1.5 μg of pronase 2 hr at 42 ºC then incubated at 65 ºC over night to reverse the crosslinks. The ChIP’ed DNA was purified by phenol/chroloform treatment and precipitated with LiCl and 70% ethanol.

For ChIP using anti-Smad1 antibody (BioMatrix, mouse monoclonal), MEFp2 cells were starved for 16 hours and exposed to rBMP2 protein at 25ng/mL in serum-free medium for 1.5 hours. Cells were fixed with 1mM Disuccinimidyl Glutarate (Thermo Scientific, Rockford, IL) for 20 min and 1% formalin for 10 min, and crosslinked cell lysates underwent fragmentation by sonication and incubated with antibody bound to protein G-sepharose bease (50% slurry) overnight at 4 ºC. Purification of ChIP’ed DNA was done as above. RNA samples at 0h, 1h, 3h, 6h, 12h and 24h were analyzed on expression array using GeneChip Mouse Genome 430 2.0 Array (Affymetrix) as above.

**ChIP-sequencing.** Sample preparation for ChIP-sequencing was performed according to the manufacturer's instructions (Illumina), and sequencing was performed using Solexa Genome Analyzer II. Briefly, size fractionated DNA was extracted and a single adenosine was added using Klenow exo– (3' and 5' exo minus; Illumina). Illumina adaptors were then added and DNA was subjected to 20 cycles of PCR according to manufacturer's instructions. We then purified DNA and performed cluster generation and 36 cycles of sequencing on the Illumina cluster station and 1G Analyzer following the manufacturer's instructions. 36-bp single end reads were mapped to the NCBI Build #36 (UCSC mm8) reference mouse genome, using the Illumina pipeline software version 1.4. The numbers of uniquely mapped reads for MEFp2 were 10,845,082 (H3K4me3), 11,519,151 (H3K27me3) and 5,688,804 (Input), those for RasV12 were 13,246,871 (H3K4me3), 9,894,241 (H3K27me3) and 6,126,206 (Input), and that for Smad1 ChIP-sequencing was 9,417,307.

To show distribution of immunoprecipitated DNA fragments in a certain region, we set a window in genomic DNA, within which we count a number of Solexa reads uniquely mapped. To detect distribution accurately, window size was set so that Solexa read count in a window of positive regions should be > 10-20 per total reads (> 1-2 per million reads). Window size of 300 bp was used in ChIP-seq of H3K4me3, because H3K4me3 was distributed in narrow DNA regions and 300 bp were wide enough for a window. H3K27me3 is rather broad modification than H3K4me3 (Figure 1A and Supporting Figure 3A), so a wider window, 500 bp, was necessary to detect read count. The number of mapped reads per million reads within a window was regarded as the modification status for the center position of the window. Within ±2 kb from TSS of each gene, the maximum number of mapped reads per million reads in a window size of 300bp (H3K4me3) or 500bp (H3K27me3) was regarded as the epigenetic status of each gene. When the number was >4 and <3 for H3K4me3, the gene was regarded as H3K4me3(+) and (-), respectively. When the number was >1.5 and <1 for H3K27me3, the gene was regarded as H3K27me3(+) and (-), respectively. We confirmed active state of H3K4me3(+) or H3K27me3(-) genes and repressive state of H3K4me3(-) or H3K27me3(+) genes, by comparing average expression levels (Supporting Figure S5). For Smad1, a window of 300 bp was used as Smad1 distribution was narrow. When the number of mapped reads per million reads was >2 within a window of 300 bp, the region was regarded as Smad1 binding(+), and the center position of the window was regarded as Smad1 binding site. ChIP-Solexa data is available at GEO datasets (#GSE18125).

**Methylated DNA immunoprecipitation (MeDIP)-sequencing.** MeDIP was performed as we previously reported MeDIP-chip [2-4]. Briefly, 6 μg each of genomic DNA of MEFp2 and RasV12 cells was fragmented by sonication, and immunoprecipitated by anti 5-methylcytocine monoclonal antibody (kindly supplied by Dr. K. Watanabe, Toray Research Center, Inc.). MeDIPed sample was amplified and sequenced using Solexa Genome Analyzer II as ChIP-sequencing was done. The numbers of uniquely mapped reads for MEFp2 and RasV12 cells were 9,663,324 and 11,319,506, respectively. Window size of 500 bp was used to calculate the number of mapped reads per million reads for the position at the center of the window. MeDIP-seq data is available at GEO datasets (#GSE18125).

**Motif Analysis.** The motifs were searched for using GADEM [5] or DME [6], and drawn by STAMP (http://www.benoslab.pitt.edu/stamp/). The Smad1 binding sites in the whole genome regions or in TSS regions, and their flanking sequences (within ±200bp from the binding sites) were analyzed. For GADEM, motifs were obtained by GADEM version1.3 (http://www.niehs.nih.gov/research/resources/software/gadem/index.cfm) with default parameters except -posWt (Weight profile for positions on the sequence) = 1, -pv (P-value cutoff) = 0.00001, -em (Number of EM steps) = 20, and -fullScan = 1. For DME, motifs were obtained by DME2 (http://rulai.cshl.edu/dme/) using ZOOPS model with default parameters except -w (minimum desired motif width) = 10 and -n (number of motifs to produce) =10.

**Immunoblot analysis.** Aliquots of protein were subjected to SDS/PAGE followed by immunoblot analysis using antibodies against FLAG (F7425, Sigma, rabbit polyclonal, 1:500 dilution, for Ras), Smad1 (#9743, Cell Signaling, rabbit polyclonal, 1:1,000 dilution), phospho-Smad1/5/8 (#9511, Cell Signaling, rabbit polyclonal, 1:1,000 dilution), Myc (9E10, mouse monoclonal, 1:500 dilution, for Smad6), V5 (R960-25, invitrogen, mouse monoclonal, 1:5,000 dilution), pan-Akt (C67E7, Cell Signaling, rabbit monoclonal, 1:1,000 dilution), phospho-Akt (D9E, Cell Signaling, rabbit monoclonal, 1:2,000 dilution) and α-Tubulin (DM1A, Sigma, mouse monoclonal, 1:5,000 dilution) as primary antibodies, and against rabbit IgG (sc-2004, Santa Cruz, 1:5,000 dilution) and mouse IgG (sc-2005, Santa Cruz, 1:5,000 dilution) as secondary antibodies. Proteins were transferred to nitrocellulose and the resulting immunoblots were visualized using Amersham ECL Plus (GE Healthcare) and LAS-3000 (Fujifilm, Japan). Density of bands was analyzed by densitometry in LAS-3000.

**Cellular immunofluorescence.** Cells were washed with PBS three times and fixed with 4% paraformaldehyde for 5 min. After washed with PBS three times, cells were permeabilized with 0.2% Triton X-100 in PBS for 10min. Phosphorylated Smad1/5/8 was detected using antibody against phospho-Smad1/5/8 (#9511, Cell Signaling, 1:50 dilution) as primary antibody, and green-fluorescent Alexa Fluor 488 dye-labeled anti-rabbit antibody (A11034, Invitrogen, 1:200 dilution) as secondary antibody. RasV12 with N-terminal FLAG tag and Smad6 with N-terminal Myc tag were detected using antibody against FLAG (F7425, Sigma, rabbit polyclonal) and Myc (9E10, Santa Cruz, mouse monoclonal) as primary antibody, respectively, and Alexa Fluor 594 anti-rabbit antibody and Alexa Fluor 488 anti-mouse antibody (Invitrogen) as secondary antibody. Photographs were taken with Biozero BZ-8100 (KEYENCE, Osaka, Japan).

**Senescence-associated β-galactosidase (SA-βgal) analysis.** MEFp2 and infected MEFs on day 10, and RasV12-IMR90 on day 7 underwent SA-βgal staining. Cells were washed twice with PBS and immersed in fixation buffer (2% (w/v) formaldehyde, 0.2% (w/v) glutaraldehyde in PBS) for 5 min. After two additional PBS washes, the cells were allowed to stain overnight in staining solution (40 mM citric acid/sodium phosphate, pH 6.0, 150 mM NaCl, 2.0 mM MgCl_2_, 5 mM K_4_[Fe(CN)_6_], 5 mM K_3_[Fe(CN)_6_], 1 mg/mL X-gal).

**Growth curve.** Infected MEFs were counted on days 3, 7, 10, 14, 17, 21 using Countess automated cell counter (Invitrogen) and seeded at density of 1×10^5^ cells / 6-cm dish for every passage. Infected IMR90 were counted on days 4, 8, 12 and 16 similarly, and seeded at density of 2.5×10^5^ cells / 6-cm dish. Mean number of three dishes was calculated and used to draw growth curve.

**Quantitative real-time RT-PCR.** Total RNA was treated with DNase I (Invitrogen), and cDNA was synthesized from 1 μg of total RNA using a Superscript III kit (Invitrogen). Real-time RT-PCR was performed using SYBR Green and iCycler Thermal Cycler (Bio-Rad Laboratories). For mouse cells, the number of molecules of *p16*, *p19*, *Bmp2*, *Smad6*, *Nog*, *Parvb*, *Ezh2*, and *Jmjd3* in a sample was measured by comparing with standard samples that contained 10^1^ to 10^6^ copies of the genes. The quantity of mRNA of each gene was normalized to that of *Ppib*, and relative expression levels compared to that in MEFp2 were shown. For human cells, the number of molecules of *BMP2*, *SMAD6* and *NOG* in a sample was measured by comparing with standard samples that contained 10^1^ to 10^6^ copies of the genes. The quantity of mRNA of each gene was normalized to that of *PPIA*, and relative expression levels compared to that in IMR90 were shown. The experiment was triplicated and mean and standard error were calculated and shown. The PCR primers and conditions are shown in Supporting Table S7.

**Quantitative real-time ChIP-PCR and MeCIP-PCR.** ChIP and MeDIP samples were amplified by real-time PCR using SYBR Green and iCycler Thermal Cycler (Bio-Rad Laboratories) and quantified by drawing the standard curve using 20, 2, 0.2, and 0.02 ng/μL sonicated genomic DNA of MEF. The quantity of ChIP’ed DNA of an analyzed region was normalized to that of Actb in each sample of MEFp2, mock, RasG12 and RasV12. The PCR primers and conditions are shown in Supporting Table S8.

**Bisulfite treatment and bisulfite sequencing.** DNA methylation status was analyzed by bisulfite sequencing as previously described [7]. Briefly, 500ng of genomic DNA of MEFp2 and RasV12 cells underwent sonication in 30 seconds by Bioruptor (COSMO BIO, Tokyo, Japan). Fragmented DNA was denatured in 0.3 N NaOH then subjected to 15 cycles of 30 seconds at 95 °C and 15 minute incubation in 3.6 M sodium bisulfite and 0.6 mM hydroquinone at 50 °C. The samples were desalted with the Wizard DNA Clean-Up system (Promega, Madison, WI), desulfonated in 0.3 N NaOH at room temperature for 5 minutes, then purified by ethaol precipitation. The bisulfite-treated DNA was finally suspended in 20 μL of distilled water. For bisulfite sequencing, 1 μl was used as a template for PCR with the primers common for methylated and unmethylated DNA sequences. The primers and PCR conditions are available at Supporting Table S9. PCR products were cloned into pGEM-T Easy vector (Promega), and 9-10 clones each were cycle-sequenced using T7 and Sp6 primers.

**References**

1. Oshima H, Itadani H, Kotani H, Taketo MM, Oshima M (2009) Induction of prostaglandin E2 pathway promotes gastric hamartoma development with suppression of bone morphogenetic protein signaling. Cancer Res 69: 2729-2733.

2. Hayashi H, Nagae G, Tsutsumi S, Kaneshiro K, Kozaki T, et al. (2007) High-resolution mapping of DNA methylation in human genome using oligonucleotide tiling array. Hum Genet 120: 701-711.

3. Yagi K, Akagi K, Hayashi H, Nagae G, Tsuji S, et al. (2010) Three DNA methylation epigenotypes in human colorectal cancer. Clin Cancer Res 16: 21-33.

4. Deng YB, Nagae G, Midorikawa Y, Yagi K, Tsutsumi S, et al. (2010) Identification of genes preferentially methylated in hepatitis C virus-related hepatocellular carcinoma. Cancer Sci 101: 1501-1510.

5. Li L (2009) GADEM: a genetic algorithm guided formation of spaced dyads coupled with an EM algorithm for motif discovery. J Comput Biol 16: 317-329.

6. Smith AD, Sumazin P, Zhang MQ (2005) Identifying tissue-selective transcription factor binding sites in vertebrate promoters. Proc Natl Acad Sci U S A 102: 1560-1565.

7. Kaneda A, Kaminishi M, Yanagihara K, Sugimura T, Ushijima T (2002) Identification of silencing of nine genes in human gastric cancers. Cancer Res 62: 6645-6650.
